# Critical appraisal of patient-reported and clinician-reported outcome measures assessing functional outcomes in lower extremity bone sarcoma patients—a COSMIN-based systematic review

**DOI:** 10.1186/s41687-026-01099-w

**Published:** 2026-06-06

**Authors:** Kiki J. Blom, Leonie G. Tigelaar, Willem P. Bekkering, Jos A. M. Bramer, Hendrik W. B. Schreuder, Sherron Furtado, Emanuela Palmerini, Nathalie Gaspar, Sandra J. Strauss, Michiel A. J. Van de Sande, Heleen Maurice-Stam, Johannes H. M. Merks, Lianne M. Haveman

**Affiliations:** 1 Princess Máxima Center for Pediatric Oncology, Utrecht, the Netherlands; 2 Department of Orthopedic Surgery, Amsterdam University Medical Center, Location AMC, Amsterdam, the Netherlands; 3 Department of Orthopedic Surgery and Sports Medicine, Amsterdam Movement Sciences, Cancer Center Amsterdam, Amsterdam University Medical Centers, University of Amsterdam, Amsterdam, the Netherlands; 4 Department of Orthopedic Surgery, Radboud University Medical Center, Nijmegen, the Netherlands; 5 Royal National Orthopaedic Hospital (RNOH) NHS Trust, Stanmore, UK; 6 Osteoncology, Bone and Soft Tissue Tumors and Innovative Therapies, IRCCS Istituto Ortopedico Rizzoli, Bologna, Italy; 7 Department of Oncology for Child and Adolescent, Gustave Roussy Cancer Campus, Villejuif, France; 8 Department of Oncology, University College London Cancer Institute, London, UK; 9 Department of Orthopedic Surgery, Leiden University Medical Center, Leiden, the Netherlands; 10 Division of Imaging and Oncology, University Medical Center Utrecht, Utrecht University, Utrecht, the Netherlands

**Keywords:** COSMIN, Functional status, International classification of functioning, Disability and health, Measurement properties, Neoplasms, Bone tissue, Outcome assessment, Health care

## Abstract

**Purpose:**

Local therapy for bone sarcoma often significantly impacts patients’ physical abilities and participation in daily life, which underscores the need for accurate assessment of these outcomes to guide optimal care. This study aims to 1) evaluate the measurement properties of eight patient- and clinician-reported outcome measures identified in a recent systematic review (2024) as being used more than once in this population, and 2) recommend the most suitable instruments for evaluation in clinic and research.

**Methods:**

We searched MEDLINE and Embase in July 2024 for studies assessing the development and/or measurement properties of the original version of the Baeck equestionnaire, Functional Mobility Assessment, Karnofsky Performance Status, Mankin score, MusculoSkeletal Tumor Society 1987 and 1993-Lower extremity (LE)scores, Reintegration to Normal Living (RNL) Index and Toronto Extremity Salvage Score (TESS) – LE. Full text availability was required. Measurement instruments were evaluated using COSMIN guidelines (July 2024 – January 2025).

**Results:**

Seventy-three articles evaluating measurement properties of one of the eight instruments were included. The Baecke questionnaire, RNL Index and TESS-LE had clearly defined constructs of which only the RNL Index and TESS-LE demonstrated sufficient overall content validity (low- and moderate-quality evidence). The remaining instruments lacked clear construct definitions, resulting in indeterminate or insufficient content validity, and therefore cannot be recommended for use. Structural validity of the TESS-LE was inconsistent leading to indeterminate internal consistency, currently preventing its recommendation. Additionally, the evidence supporting sufficient measurement properties of the RNL Index was too limited to justify its use.

**Conclusion:**

A clearly defined construct is the cornerstone of a measurement instrument and was lacking in most cases. The TESS-LE and RNL Index show promise due to their sufficient content validity, but require further refinement before they can be recommended as standard instruments to assess physical abilities and participation in daily life in bone sarcoma patients. Advancing measurement of these outcomes requires comprehensive validation and refinement of existing instruments to overcome their current limitations, or the development of new, reliable, and valid instruments tailored to this population.

**Registration:**

Study protocol registration in the PROSPERO database:CRD42022353668.

**Supplementary Information:**

The online version contains supplementary material available at 10.1186/s41687-026-01099-w.

## Abstract (plain language summary)

Bone sarcoma is a type of cancer that affects the bones. Surgery is an important part of treatment, and can have a big impact on a person’s ability to move and perform daily activities. It is important to measure these abilities, i.e. functional outcomes, accurately to help doctors provide the best care and support for patients.

In this study, we assessed eight commonly used tools that measure how well bone sarcoma patients can function physically and take part in everyday activities during and after treatment. We wanted to find out if these tools are reliable and suitable for these patients. Our review showed that most of these tools do not clearly explain what they are measuring, making their results hard to trust. Only two tools, the Toronto Extremity Salvage Score-Lower extremity (TESS-LE) and the Reintegration to Normal Living (RNL) Index, had a clear focus and were useful to some extent, but they still need improvements to be fully reliable.

Our findings show that better tools are needed to measure how well patients function during and after treatment for a bone sarcoma. Future research should work on improving current tools or developing new ones. This will help healthcare providers make more informed decisions and improve patient care.

**Supplementary Information:**

The online version contains supplementary material available at 10.1186/s41687-026-01099-w.

## Introduction

Bone sarcomas are rare malignancies, with European standardized incidence rates in the Netherlands of approximately 0.25 per 100,000 for osteosarcoma and 0.15 per 100,000 for Ewing sarcoma [1]. Standard treatment typically consists of induction chemotherapy, followed by local therapy and subsequent consolidation chemotherapy. Local therapy, including invasive surgery and/or radiotherapy, is crucial for bone sarcoma treatment but often has life-changing consequences, particularly affecting patients’ physical abilities [2–4]. Accurate assessment of functional outcomes, defined as patients’ physical abilities and participation in daily life, is essential to guide preoperative counseling, postoperative care, rehabilitation and follow-up. Various measurement instruments exist to evaluate these outcomes, encompassing different administration modes, such as performance-based outcome measures (PerBOMs), clinician-reported outcome measures (ClinROMs), and patient-reported outcome measures (PROMs), and addressing different functional outcome domains according to the World Health’s Organization’s (WHO) International Classification of Functioning, disability and health (ICF).

In a previous systematic review, we identified 42 unique instruments for assessing functional outcomes in pelvic or lower extremity bone sarcoma patients [5]. Among these, PROMs and ClinROMs are mostly included for outcome evaluation in clinic and clinical trials. Eight PROMs and ClinROMs were used more than once and were applicable to both pelvic and lower extremity bone sarcoma patients: the MusculoSkeletal Tumor Society 1987 and 1993-Lower extremity scores (MSTS87 score, MSTS93 score-LE), the Toronto Extremity Salvage Score – lower extremity (TESS-LE), the Karnofsky Performance Status (KPS), the Mankin score, the Functional Mobility Assessment (FMA), the Baecke questionnaire, and the Reintegration to Normal Living (RNL) Index [5]. Most instruments assess the ICF’s activity domain, except for the RNL Index which addresses the participation domain.

Selecting the most appropriate measurement instrument for a specific population and context requires critical appraisal of its measurement properties. To date, no review has comprehensively assessed all measurement properties of functional outcome instruments used in bone sarcoma patients. To standardize the process of measurement instrument evaluation, the COnsensus-based Standards for the selection of health Measurement INstruments (COSMIN) framework provides a structured approach for evaluating and selecting appropriate instruments [6, 7].

This systematic review aims to: 1) evaluate, according to the COSMIN guidelines, the measurement properties of the eight PROMs and ClinROMs assessing physical abilities and participation in daily life in patients with lower extremity and pelvic bone sarcoma as identified in the previous review, and 2) recommend the most suitable instruments for evaluation in clinic and research.

The findings will guide clinicians and researchers in selecting optimal instruments, thereby enhancing the quality of assessments and supporting better-informed decision-making. This review will also contribute to standardizing assessments in future studies, including upcoming trials within the FOSTER consortium and the EuroEwing Consortium (EEC). By encouraging the adoption of high-quality instruments, potentially streamlining the range of tools used, this review will facilitate cross-study and international comparisons, advancing the global understanding of functional outcomes in bone sarcoma patients.

## Methods

The study protocol was registered in PROSPERO (CRD42022353668).

### Eligibility criteria

Studies were eligible if they evaluated the development and/or measurement properties of one of the eight PROMs and ClinROMs that measure functional outcomes in patients with lower extremity or pelvic bone sarcoma, as identified in the previous review [5]. As COSMIN evaluations are resource-intensive, we limited our scope to instruments used more than once to ensure feasibility. Eligible studies were peer-reviewed articles available in English. Studies were excluded if they aimed to validate another instrument, did not include the instrument’s original version, or lacked full text availability. Reference lists of included articles were screened for completeness.

### Study identification and selection

On July 1, 2024, we conducted seven systematic literature searches in MEDLINE (via PubMed) and Embase (via http://www.embase.com): one combined search for the MSTS87 score and MSTS93 score-LE, and one for each remaining instrument [8, 9]. The searches incorporated the instrument’s full name and a measurement property filter by Terwee et al. [10]. Detailed search strategies are available in Online Resource 1. Two reviewers (KJB and LGT) independently screened titles/abstracts and full texts, resolving discrepancies through discussion.

### Evaluation of measurement instruments

Measurement instruments were evaluated according to the COSMIN framework. In line with COSMIN terminology, we use the terms patient-reported outcome measures (PROMs), clinician-reported outcome measures (ClinROMs), and performance-based outcome measures (PerBOMs), which conceptually overlap with other commonly used nomenclature, such as PROs (patient-reported outcomes), ObsROs (observer-reported outcomes), and PerfOs (performance outcomes) [11].

**Fig. 1:**
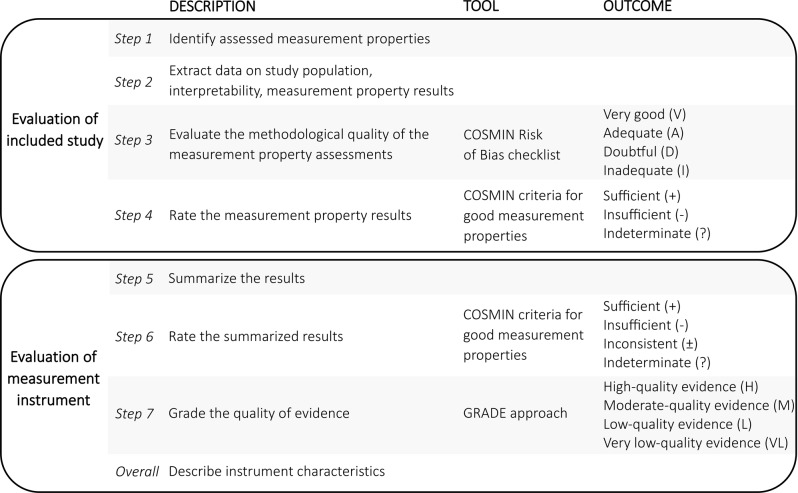
Steps for evaluating a measurement instrument using the COSMIN framework [6]. COSMIN = COnsensus-based Standards for the selection of health Measurement INstruments. GRADE = Grading of Recommendations, Assessment, Development and Evaluation

Definitions used by the framework to evaluate measurement instruments are provided in Online Resource 2 and include development, content validity, structural validity, internal consistency, cross-cultural validity/measurement invariance, reliability, measurement error, criterion validity, construct validity, responsiveness, and interpretability [6]. Evaluation followed seven steps (Fig. 1). The first four steps were repeatedly applied to the included studies assessing one of the measurement instruments. Subsequently, steps five to seven were performed for the measurement instrument. Measurement instrument characteristics were extracted throughout the process.

#### Step 1. Identified assessed measurement properties

First, the measurement properties assessed in the included study were identified.

#### Step 2. Data extraction: study population, interpretability and measurement property results

Next, data was extracted from the study on:

1. Details regarding the study population.
2. Information related to the interpretability of the instrument.
3. Results of the measurement property assessments.

#### Step 3. Evaluate the methodological quality of the measurement property assessments

The COSMIN Risk of Bias checklist was used to evaluate the methodological quality of the instrument’s development and measurement property assessments [12]. This checklist evaluates methodological criteria specific to the instrument’s design and to each measurement property under review. Criteria are rated as‘very good’, ‘adequate’, ‘doubtful’, or ‘inadequate’, and overall methodological quality rating is determined by the lowest-rated criterion (the “worst score counts” principle) to ensure that any methodological limitation is accounted for.

First, all instruments were rated for clarity of their design, including the presence of a clear description of the construct, origin of the construct (e.g. underlying theory, conceptual framework or disease model), target population and context of use (Online Resource 3). This design evaluation is a prerequisite for judgements during content validity appraisal. Second, the methodological quality of the measurement property assessments was evaluated.

#### Step 4. Rate the measurement property results

Measurement property results were rated according to the COSMIN’s criteria for good measurement properties as sufficient (+), insufficient (-) or indeterminate (?) [7]. In addition to results from content validity studies, the reviewers also conducted their own assessment of the instrument’s relevance, comprehensiveness and comprehensibility, as recommended by COSMIN. COSMIN’s criteria for this reviewer-based rating of content validity are provided in Online Resource 4. Ratings of construct validity and responsiveness were guided by a priori hypotheses as established prior to conducting the review to ensure rating consistency (Online Resource 5).

#### Step 5. Summarize the results

Measurement property results of the included studies were summarized. Results from methodologically inadequate assessments (see step 3) or with an indeterminate rating (see step 4) were included only if no higher-quality evidence was available. Otherwise, such evidence was ignored. For content validity, only results of studies of at least doubtful quality were summarized. If there were no or only inadequate content validity studies performed, final results were based on reviewers’ ratings. In cases of inconsistent results across studies, explanations were explored by examining differences in populations or methodological quality. If the inconsistency could be explained, results were summarized per subset of studies.

#### Step 6. Rate the summarized results

The summarized results were rated according to the COSMIN’s criteria for good measurement properties: sufficient (+), insufficient (-), inconsistent (±) or indeterminate (?). The summarized result was only rated inconsistent if the inconsistency could not be explained (see step 5).

#### Step 7. Grade the quality of evidence

In the final step, we graded the quality of the summarized results using the Grading of Recommendations Assessment, Development and Evaluation (GRADE) framework. This process assigns four quality of evidence levels: ‘high’, ‘moderate’, ‘low’, or ‘very low’ (Online Resource 6) [6]. A GRADE level was not assigned to indeterminate (?) results. When content validity results were based on reviewers’ ratings, quality of evidence was ‘very low’.

Steps one to four, including reviewer-based content validity ratings, were conducted independently by two reviewers (KJB and LGT) and subsequently compared to ensure accuracy. Any discrepancies were resolved through discussion. Steps five to seven were conducted collaboratively.

### Formulation of recommendations

Per COSMIN guidelines, measurement instruments can be recommended if high-quality evidence supports that all measurement properties are sufficient [6]. Instruments with high-quality evidence of any insufficient measurement property will not be recommended for use. In cases of inconclusive or insufficient evidence, further research is required. For instruments with sufficient measurement properties, feasibility and interpretability are considered in relation to the intended context of use (clinical practice and research).

We prioritized measurement properties following COSMIN standards [6]. Content validity was given the highest priority, because relevant, comprehensive, and comprehensible items are foundational to a good instrument. This requires a clear definition of the measured construct, target population, and context of use. Structural validity and internal consistency followed. When these properties were comparable, reliability, construct validity, responsiveness, and interpretability were considered to refine recommendations.

## Results

### Study selection

The results of the seven searches are documented in PRISMA flow diagrams (Online Resource 7). After removing duplicates, 8188 articles were screened by title/abstract. Of these, 120 articles proceeded to full-text review, with 65 included in the final analysis. An additional eight articles were identified through reference lists screening. These were mainly missed by the searches because the instrument’s name was not mentioned in the title/abstract. In total, 73 articles were included.

### Characteristics of measurement instruments

Of the eight instruments, five are ClinROMs and three are PROMs (Table 1). The MSTS87 score, MSTS93 score-LE, TESS-LE, and FMA are specific to bone sarcoma patients, while the others were developed for the general population or other conditions [14, 18]. Overall, the PROMs evaluated in this review tended to include more items (11–30 items) and required slightly longer completion times (5–10 minutes) than the included ClinROMs, which contained 1–20 items and generally required under five minutes. The FMA was an exception, with a completion time of up to 20 minutes. Most measurement instruments were originally developed in English, except for the Baecke questionnaire (Dutch), and the RNL Index (English and French) [13, 17]. The TESS-LE and MSTS93 score-LE are the most widely translated, into 16 and 11 languages, respectively.

**Table 1:** Characteristics of included measurement instruments of functional outcome

Instrument	Construct(s)	Target population	Mode of administration	Recall period	(Sub)scale(s), number of items	Response options	Range of scores/scoring	Completion time	Original language	Available translations
Baecke questionnaire [13]	Habitual physical activity	General population	PROM	Unknown	Work Index: 8 items Sport Index: 4 items Leisure-time Index: 4 items	5-point scale	Subscale: 1–5 Total score: 3–15	5 min	Dutch	English Brazilian-Portuguese JapanesePersian
Functional Mobility Assessment [14]	Functional mobility	Lower extremity sarcoma	ClinROM	Pain item: 1 week	6 categories 14 items	0 (worst) to 5 (best)	Total score: 0–70	20 min	English	None
Karnofsky Performance Status [15]	Performance status	Cancer patients	ClinROM	Unknown	1 item	11 levels	100–0 (normal to dead)	<1 min	English	Turkish
Mankin score [16]	Function	Patients after allograft transplantation	ClinROM	Unknown	1 item	Excellent-Good-Fair-Failure	Excellent to Failure	<1 min	English	Unknown
MusculoSkeletal Tumor Society 1987 score [8]	Functional results	Patients with limb-salvage procedures after tumor resection or amputation	ClinROM	Unknown	7 items	6-point Likert scale (0–5)	Total score: 0–35	5 min	English	None
MusculoSkeletal Tumor Society 1993 score-Lower extremity [9]	Functional results	Patients with limb-salvage procedures after tumor resection or amputation	ClinROM	Unknown	6 items	6-point Likert scale (0–5)	Total score: 0–30 converted to 0–100%	2 min	English	Brazilian Portuguese, Chinese, Danish, Finnish, French, German, Greek, Italian, Japanese, Turkish, Romanian
Reintegration to Normal Living Index [17]	Reintegration to normal living	Patients following disabling illness	PROM	Unknown	Daily Functioning: 8 items Perception of Self: 3 items	VAS (10 points)^a^	Total score: 0–110 converted to 0–100	Max 10 min	English and French	Cantonese, IGBO language
Toronto Extremity Salvage Score - Lower extremity [18]	Physical disability	Patients with bone and soft tissue sarcoma of the lower extremity	PROM	Previous week	30 items	5-point Likert scale + NA option	Total score: 0–100	±10 min	English	Arabic, Brazilian Portuguese, Chinese, Danish, Dutch, Finnish, French, German, Greek, Italian, Japanese, Korean, Mexican Spanish, Norwegian, Swedish

ClinROM = clinician-reported outcome measure; NA = not applicable; PROM = patient-reported outcome measure

^a^ Anchored by phrases 'does not describe my situation' and 'fully describes my situation

### Interpretability

Floor and ceiling effects were assessed for the FMA, MSTS87, MSTS93-LE and TESS-LE. While the FMA showed no such effects, considerable ceiling effects were observed for the MSTS87 (33%), MSTS93-LE (5–33%) and TESS-LE (0.2–26%). Additional interpretability details of all instruments, including score distributions (also within subgroups), percentages of missing items or total scores, changes over time, minimal important change (MIC) or difference (MID), and response shift, are available in Online Resource 8.

### Evaluation of measurement properties

The TESS-LE, Baecke questionnaire, KPS and MSTS93 score-LE were most extensively evaluated [15]. Since no pilot or content validity studies were available for the FMA, KPS, Mankin score, and MSTS87 score, their content validity results are based on reviewers’ ratings [16]. The measurement properties evaluations are discussed below, organized by instrument. A concise summary is presented in Tables 2 and 3, with detailed descriptions in Online Resources 9 (study population characteristics of included articles), 10 (summary of the measurement property results per instrument), 11 (detailed content validity results per included article), and 12 (extensive results of other measurement properties per included article).

**Table 2:** Methodological quality of development and content validity studies

	Design						Pilot study				Total development	Content validity					
	General design requirements				Concept elicitation study	Total design	General design	Comprehen-sibility	Comprehen-siveness	Total pilot study		Asking patients				Asking experts	
**Instrument**	**Clear construct**	**Clear origin of construct**	**Clear target population**	**Clear context of use**			**Pilot study performed in sample representing the target population**					**Relevance**	**Comprehen-siveness**	**Comprehen-sibility**		**Relevance**	**Comprehen-siveness**
Baecke [13, 19]	V	D	I	I		I								I			
FMA [14]	I	D	V	V		I											
KPS [15]	I	D	I	V		I											
Mankin [16]	I	D	V	D		I											
MSTS87 [8, 9]	I	D	V	V		I											
MSTS93-LE [9, 20–23]	I	D	V	V		I								D		D	D
RNL Index [17, 24]	V	V	V	V	D	D					D	D	D	D			
TESS-LE [18, 20, 22, 25–31]	V	V	V	V	D	D	V		D	D	D	D	D	D		D	D

Empty cells indicate that no pilot or content validity studies were performed

V = very good methodological quality; A = adequate methodological quality; D = doubtful methodological quality; I = inadequate methodological quality

Baecke = Baecke questionnaire; FMA = Functional Mobility Assessment; KPS = Karnofsky Performance Status; Mankin = Mankin score; MSTS87 = MusculoSkeletal Tumor Society 1987 score; MSTS93-LE = MusculoSkeletal Tumor Society 1993 score-Lower extremity; RNL Index = Reintegration to Normal Living Index; TESS-LE = Toronto Extremity Salvage Score – Lower extremity

**Table 3:** Summary of findings per measurement instrument: ratings of summarized results and quality of the evidence

Instrument	Content validity						Reliability				Hypotheses testing for construct validity		Responsiveness			
	Relevance	Comprehensiveness	Comprehensibility	Structural validity	Internal consistency	Cross-cultural validity/Measurement invariance	Test-retest	Inter-rater	Measurement error	Criterion validity	Other instruments	Subgroups	Gold standard	Other instruments	Subgroups	Before-after intervention
Baecke-WI [13, 32–43]	?	+ (VL)	+ (VL)	+ (M)	- (M)		+ (L)				+ (M)	+ (VL)				
Baecke-SI [13, 33–44]	?	+ (VL)	+ (VL)	+ (M)	- (M)		+ (L)				± (M)	+ (VL)				
Baecke-LI [13, 32–44]	?	- (VL)	+ (VL)	+ (M)	- (M)		+ (L)^a^ - (VL)^b^ + (VL)^c^ - (VL)^d^				± (M)	+ (VL)				
Baecke-TI [19, 33, 35–39, 41–43, 45, 46]	?	- (VL)	+ (VL)				+ (L)				± (M)	+ (VL)				- (M)
FMA [14, 47]	?	?	± (VL)		?			+ (VL)			+ (M)	+ (H)				?
KPS [48–65]	?	- (VL)	- (VL)				+ (L)	+ (M)^e^ ± (M)^f^			± (H)	+ (H)				
Mankin [16]	?	- (VL)	- (VL)													
MSTS87 [66, 67]	?	?	+ (VL)	?	?		+ (M)		?		+ (M)					?
MSTS93-LE [9, 20–23, 66, 68–72]	?	?	+ (M)	+ (H)	+ (H)		+ (M)	+ (H)	?		± (H)	+ (VL)				?
RNL-Total score [17, 24, 73, 74]	+ (L)	+ (L)	+ (L)	NA	NA					+ (H)	± (M)					?
RNL-Daily functioning [17, 24, 73]	NA	NA	NA	NA	NA					+ (H)	+ (L)					?
RNL-Perception of self [17, 24, 73]	NA	NA	NA	NA	NA					+ (H)	+ (L)					?
TESS-LE [18, 20, 22, 25–31, 67, 75–78]	+ (VL)	+ (M)	+ (M)	± (VL)	?	?	+ (H)		+ (L)		± (H)	+ (M)				± (M)

+ = measurement property sufficient; - = measurement property insufficient;? = measurement property indeterminate

H = high quality of evidence; *M* = moderate quality of evidence; L = low quality of evidence; VL = very low quality of evidence; NA = not applicable

Baecke = Baecke questionnaire; FMA = Functional Mobility Assessment; KPS = Karnofsky Performance Status; LI = Leisure-time Index; Mankin = Mankin score; MSTS87 = MusculoSkeletal Tumor Society 1987 score; MSTS93-LE = MusculoSkeletal Tumor Society 1993 score-Lower extremity; RNL = Reintegration to Normal Living Index; SI = Sports Index; TESS = Toronto Extremity Salvage Score – Lower extremity; TI = Total Index; WI = Work Index.

^a^ In the general population

^b^ In patients with chronic low back pain

^c^ In women with hip disorders

^d^ In adults with HIV/AIDS

^e^ Oncologist versus oncologist

^f^ Raters with distinct medical background

### Baecke questionnaire

The Baecke questionnaire is a PROM assessing habitual physical activity across three indices: occupational physical activity (Work Index), sports during leisure time (Sports Index), and physical activity during leisure time excluding sports (Leisure-time Index). Although developed in young adults (19 to 31 years old), its target population is not clearly defined [13]. The three factor structure was confirmed through confirmatory factor analysis, though a total score (Total Index) is commonly used without structural validation [40]. A study in cardiac disease patients identified a two-factor structure, but since this structure is not used in practice, we focused on the original three-factor structure [32]. The Baecke questionnaire has been mainly studied in healthy adults, with some research in patients with chronic low back pain, hip disorders, HIV/AIDS, and cardiac disease (Online Resource 9).

#### Work Index (WI)

The WI’s relevance could not be determined due to the unclear target population and context of use, whereas comprehensiveness and comprehensibility were sufficient. Internal consistency was insufficient (moderate-quality evidence), but test-retest reliability was sufficient (low-quality evidence). Construct validity was sufficient based on comparison with other instruments (moderate-quality evidence) and subgroup comparisons (very low-quality evidence).

#### Sports Index (SI)

Similar to the WI, SI’s relevance was indeterminate, comprehensiveness and comprehensibility were sufficient, internal consistency was insufficient (moderate-quality evidence), and test-retest reliability sufficient (low-quality evidence). Construct validity was sufficient when comparing subgroups (very low-quality evidence) but inconsistent when comparing with other instruments.

#### Leisure-time Index (LI)

In line with the WI and SI, LI’s relevance was indeterminate. Since we found key concepts missing, comprehensiveness was insufficient. Items and response options were appropriate resulting in sufficient comprehensibility. Internal consistency was insufficient (moderate-quality evidence). Test-retest reliability varied: sufficient in healthy populations and women with hip disorders but insufficient in patients with chronic low back pain and adults with HIV/AIDS. Evidence supporting test-retest reliability ranged from low in healthy populations to very low in other subgroups. Construct validity was sufficient for subgroup comparisons (very low-quality evidence) but inconsistent with other instruments (moderate-quality evidence).

#### Total Index (TI)

Following the ratings of the individual indices, relevance of the TI was indeterminate, comprehensiveness insufficient, and comprehensibility sufficient. Test-rest reliability was sufficient, although supported by low-quality evidence. Construct validity assessed through subgroup comparisons was sufficient (very low-quality evidence), but inconsistent when compared with other instruments. One study reported insufficient results for responsiveness (moderate-quality evidence).

### Functional Mobility Assessment (FMA)

The FMA is a ClinROM evaluating functional mobility in lower extremity sarcoma survivors [14]. It combines independent PerBOMs and ClinROMs into a total score. The development process is poorly documented, and the construct is unclearly defined. Two studies evaluated its measurement properties in sarcoma patients, with one also including individuals with non-cancerous conditions [14, 47]. Additionally, one study established reference values in a healthy population stratified by gender and age [79].

Relevance and comprehensiveness could not be determined due to the unclear construct, and item wording was insufficient, leading to inconsistent comprehensibility. Internal consistency was indeterminate because structural validity was not assessed. Reliability was rated as good, although quality of evidence was very low. Construct validity was sufficient, with high- and moderate-quality evidence supporting correlations with other instruments and subgroup differentiation, respectively. Responsiveness was indeterminate, as no hypotheses could be formulated from the existing literature or current knowledge to assess the results.

### Karnofsky Performance Status (KPS)

The KPS is a ClinROM developed to assess the clinical effects of therapy in cancer patients by measuring performance status [15]. Most studies involved adult and/or elderly cancer patients, although a few included non-cancerous diseases such as HIV (Online Resource 9).

Documentation on the KPS development is sparse, and its construct is unclearly defined, making relevance undeterminable. Its single-item format led to insufficient comprehensiveness. Comprehensibility was also insufficient due to inadequate wording of the item and response options. Test-retest reliability showed sufficient consistency, although evidence quality was low. Inter-rater reliability was sufficient among oncologists but inconsistent across raters from different professional backgrounds. Construct validity was sufficient when assessed by subgroup comparisons (high-quality evidence) but inconsistent when compared with other instruments (high-quality evidence).

### Mankin score

The Mankin score is a disease-specific ClinROM designed to assess function after allograft transplantation [16]. Relevance could not be determined due to an unclear construct. Comprehensiveness was insufficient because it includes only one item, and comprehensibility was insufficient due to unclear definition of the item and response options with multiple components.

### MusculoSkeletal Tumor Society (MSTS) 1987 score

The MSTS87 score evaluates functional results of limb-sparing procedures and amputation after tumor resection [8]. Studies evaluating its measurement properties include adult patients with soft tissue and bone tumors treated with (limb-sparing) surgery (Online Resource 9). Due to poorly documented development and unclearly defined construct, relevance and comprehensiveness were indeterminate. Imprecise wording of items and response options led to insufficient comprehensibility. Since the instrument’s unidimensionality could not be confirmed, internal consistency was indeterminate. Test-retest reliability was sufficient, based on one adequate study. Measurement error was indeterminate as the standard error of measurement (SEM) relied on Cronbach’s alpha. Construct validity was sufficient supported by comparisons with other instruments. Responsiveness could not be determined due to uninterpretable data.

### MusculoSkeletal Tumor Society (MSTS) 1993 score – Lower Extremity (LE)

The MSTS93 score is a revision of the MSTS87 score, replacing gradations of motion, strength, stability and deformity with items specific to the upper and lower extremities resulting in two extremity-specific versions. Multiple studies have assessed the measurement properties of the MSTS93 score-LE, primarily in patients with bone and/or soft tissue tumors treated with surgery, mostly limb-sparing (Online Resource 9). Content validity in Brazilian-Portuguese and Finnish studies showed sufficient comprehensibility supported by moderate-quality evidence [20, 23]. French and Turkish versions also studied content validity but did not report results [21, 22]. Due to the unclear construct, relevance and comprehensiveness could not be determined.

The instrument’s unidimensionality was confirmed, with sufficient internal consistency (both high-quality evidence). Test-retest and inter-rater reliability were sufficient based on moderate- and high-quality evidence, respectively. Measurement error was indeterminate as SEM relied on Cronbach’s alpha. Construct validity yielded inconsistent results by comparisons with other instruments but sufficient results by subgroup comparisons, supported by very low-quality evidence. Responsiveness could not be determined due to uninterpretable data.

### Reintegration to Normal Living (RNL) index

The RNL Index is a PROM designed to measure reintegration to normal living, a concept closely linked to functional status [17, 73]. It evaluates the impact of disease and treatment on patients’ lives following disabling illnesses. The RNL Index consist of two subscales: Daily Functioning and Perception of Self, which together contribute to a total score. These subscales are not intended to measure distinct constructs independently but collectively contribute to the overall construct. Consequently, content validity was assessed for the RNL Index as a whole, and evaluations of structural validity and internal consistency are not applicable. However, other measurement properties can be appropriately assessed at the subscale level and are reported when available.

The studies assessing the RNL Index included adult patients with various conditions, such as cancer, myocardial infarction, stroke, post-polio syndrome, severe osteoarthritis and leprosy (Online Resource 9).

#### Daily functioning

The IGBO version of the Daily Functioning subscale demonstrated high correlations with the original English version, indicating sufficient criterion validity supported by high-quality evidence. Construct validity was sufficient when compared to other instruments, albeit based on low-quality evidence. Responsiveness was indeterminate due to the lack of established hypotheses to rate the results.

#### Perception of self

The IGBO version of the Perception of Self subscale demonstrated strong correlations with the original English version, ensuring sufficient criterion validity with high-quality evidence. Construct validity by comparison to other instruments was also sufficient, although supported by low-quality evidence. As with the Daily Functioning subscale, responsiveness could not be determined due to the absence of established hypotheses.

#### Total score

One content validity study of the IGBO version showed sufficient relevance, comprehensiveness and comprehensibility. Since no content validity studies were found for the English version, content validity results were based on reviewers’ ratings, which also showed sufficient relevance, comprehensiveness and comprehensibility.

Comparing the total score of the IGBO version to the English original showed strong correlations, indicating sufficient criterion validity. Construct validity by comparison to other instruments yielded inconsistent results. Although responsiveness was assessed, results could not be interpreted and rated indeterminate.

### Toronto Extremity Salvage Score (TESS) – Lower Extremity (LE)

The TESS-LE is a disease-specific PROM designed to assess physical disability in patients with bone and soft tissue sarcomas [25]. Physical disability is defined by activity limitations and restrictions in mobility, self-care, and daily tasks, as outlined by the International Classification of Impairments, Disabilities and Handicaps (ICIDH) [80].

Studies evaluating its measurement properties often included patients with benign aggressive and malignant sarcomas treated with tumor surgery, limb salvage techniques and/or amputation (Online Resource 9).

The TESS-LE’s content is relevant, comprehensive, and comprehensible, resulting in sufficient content validity. Structural validity was only assessed inadequately yielding inconsistent factor structures and leading to indeterminate internal consistency. Test-retest reliability, measurement error and construct validity (via subgroup comparisons) were sufficient. Construct validity based on comparisons with other instruments showed inconsistent results: correlations with instruments measuring the same construct were as expected, but correlations with instruments measuring different constructs were often too strong. Responsiveness results were also inconsistent, primarily due to variability in postoperative improvements observed between three and six months.

### Recommendations

The FMA, KPS, Mankin score, MSTS87 score and MSTS93 score-LE lack clear construct descriptions, resulting in indeterminate overall content validity. The Baecke questionnaire, RNL Index, and TESS-LE have clearly defined constructs, with only the TESS-LE and RNL Index demonstrating sufficient overall content validity. However, inconsistent structural validity and therefore indeterminate internal consistency of the TESS-LE limit its recommendation for use. Likewise, limited evidence for other measurement properties of the RNL Index introduces too much uncertainty regarding its quality to recommend its use.

## Discussion

This study systematically evaluated the measurement properties of eight instruments used to assess physical abilities and participation in daily life, in patients with lower extremity and pelvic bone sarcoma, following the COSMIN guidelines. Despite the need for reliable and valid tools, none of the reviewed instruments met the standards for recommendation.

A previous review by Pakulis et al. (2005) evaluated several PROMs and ClinROMs measuring functional outcomes for adolescents with bone sarcoma, focusing on their development, content validity, and feasibility [81]. They concluded that none could be recommended, emphasizing the need for further research. Over two decades later, our review reveals that this gap persists. Another review by Furtado et al. also evaluated measurement properties of functional outcome instruments in bone sarcoma patients but focused exclusively on PerBOMs, restricting comparison with the instruments we reviewed [82].

Functional outcomes is a broad concept encompassing various constructs, as reflected by the WHO’s ICF and the diverse instruments evaluated. Clear construct definition is essential during instrument development to ensure suitability for intended use. Among the reviewed instruments, only the Baecke questionnaire, RNL Index and TESS-LE had clearly defined constructs, with sufficient overall content validity limited to the TESS-LE and RNL Index. Most reviewed instruments were developed over 20 years ago when standards for instrument design were less stringent. As foundational elements of older instruments are rarely revisited, their limitations may remain undetected, potentially reducing their relevance to current standards and contexts, or allowing flawed instruments to persist in use. This review demonstrated their limitations, emphasizing the need to reconsider their use.

Beyond content validity, structural validity and internal consistency are important to consider. For the TESS-LE, structural validity results were inconsistent across studies, which might partially be due to insufficient sample sizes in factor analyses. None of the included studies met the COSMIN-recommended minimum of five patients per item (150 participants for the TESS-LE), resulting in inadequate methodological quality of all assessments. While this threshold can be difficult to reach given the rarity of sarcomas, achieving sufficient sample size is important for reliable structural validity results. In addition to sample size limitations, variability in study populations, such as differences in sarcoma types, follow-up time, and age groups, may also have contributed to the inconsistent findings. Given the superior content validity of the TESS-LE compared to other instruments, further research should prioritize refining its structure using a sufficiently large and representative sample. Two approaches may be considered depending on the authors’ preferences, context and aims: (1) conduct exploratory factor analysis to identify a new structure, revise items or define subscales, and then confirm this with confirmatory factor analysis; or (2) revise the instrument based on current findings and context-specific considerations, followed by confirmatory analysis.

The RNL Index also demonstrated sufficient content validity and is conceptually relevant for evaluating reintegration following disabling illness. However, its applicability remains uncertain due to limited and low-quality evidence for other essential measurement properties. As structural validity and internal consistency are not applicable to the RNL Index, other measurement properties as reliability, construct validity and responsiveness are important for assessing its performance. These were either not assessed, only partially evaluated, or rated as indeterminate due to methodological shortcomings, limiting confidence in the instrument’s overall performance.

In addition to measurement properties, interpretability is important to consider when selecting instruments for clinical and research use. The observed ceiling effects in the MSTS87, MSTS93-LE, and TESS-LE may reduce their sensitivity to distinguish between high-functioning patients and limit their responsiveness to detect improvements in this subgroup.

During the evaluation of measurement properties, we encountered several challenges. Content validity studies were sparse, possibly due to insufficient recognition of its importance. As a result, content validity assessments frequently relied on reviewer ratings, which are inherently suboptimal. In several cases, the appropriateness of items could not be judged because the instrument’s design, specifically the construct, target population, or context of use, was insufficiently defined. Without this clarity, determining the instrument’s relevance, comprehensiveness and comprehensibility was not feasible. Future studies could strengthen the content validity of existing instruments by conducting research to clarify or explicitly define their intended constructs where these remain insufficiently described. For instruments with many translations, such as the MSTS93 score-LE and TESS-LE, cross-cultural validity is important but underexplored. Construct validity testing also often yielded inconsistent or indeterminate results, likely partly due to the challenge of formulating appropriate hypotheses to support interpretation of the results. While such hypotheses do not necessarily need to be pre-defined by the original study, they must be reasonably derivable from existing theory or literature. When this is not possible, construct validity cannot be meaningfully assessed. Additionally, while instruments typically correlated as expected with similar constructs, correlations were often unexpectedly high with distinct constructs, further complicating interpretation. To improve construct validity assessments, future studies should ensure that testable and interpretable hypotheses can be formulated to support the quality and applicability of the resulting evidence.

Certain methodological decisions were made to ensure consistency in this review. The FMA was included for completeness, despite being a combined clinician-reported and performance-based measure. Additionally, alternative versions of some instruments (e.g., with different response scales or item modifications) were excluded, as these represent distinct instruments requiring separate evaluation.

PROMs and ClinROMs capture constructs from patient’s and clinician’s perspectives and may therefore yield different results. Both types of instruments were included in this review because they are commonly used in clinical practice and trials involving patients with bone sarcoma. While PROMs are increasingly preferred for outcome assessment, ClinROMs may remain appropriate in certain contexts [83]. We therefore present the measurement properties of both to support informed selection of outcome measures across different settings.

This review has several limitations. First, we included only PROMs and ClinROMs from the initial systematic review that appeared more than once in literature, potentially excluding other relevant functional outcome instruments, such as quality-of-life subscales, newly developed instruments like the Sarcoma Assessment Measure, Patient-Reported Outcomes Measurement Information System (PROMIS) item banks or PerBOMs [84–86]. Secondly, measurement properties were sometimes poorly reported, requiring judgment by the research team, particularly for content validity, hypotheses testing, and responsiveness. Therefore, other researchers might draw differing conclusions. To ensure transparency, we documented all evaluations in tables and online resources.

## Conclusion

The Baecke questionnaire, FMA, KPS, Mankin score, MSTS87, and MSTS93 score-LE lack sufficient content validity, limiting their ability to reliably capture functional outcomes in bone sarcoma patients. The TESS-LE and RNL Index demonstrated better-defined constructs and sufficient content validity, but still require refinement. Specifically, TESS-LE’s structural validity is inconsistent, while the RNL Index lacks sufficient evidence to support its measurement properties.

Moving forward, selecting functional outcome measures for trials and clinical practice requires careful consideration. For instruments with insufficient content validity, dedicated studies to establish or strengthen content validity are important. When evidence for other measurement properties is limited, available instruments could be used in clinical trials to further assess and refine these properties. Clinical trials, particularly in rare diseases such as bone sarcoma, may offer valuable opportunities to generate high-quality evidence due to larger, well-characterized cohorts. This approach has been adopted in the upcoming FOSTER trial, which includes the TESS for functional outcome assessment. It also applies to functional outcome instruments with demonstrated sufficient measurement properties in other populations but not yet used in bone sarcoma patients. Including these instruments in trials allows for additional validation in this population. Finally, developing new, bone sarcoma-specific instruments offers an opportunity to address existing gaps and provide more precise assessments.

Each approach has implications for ongoing and future research, influencing study design or interpretation of outcomes. Above all, they highlight the need for careful selection of measurement instruments to ensure meaningful and reliable functional outcome assessments in bone sarcoma patients [87, 88].

## Electronic supplementary material

Below is the link to the electronic supplementary material.

Supplementary material 1

## Data Availability

The COSMIN guidelines and template data collection forms can be found at www.cosmin.nl. Detailed risk of bias assessments are available from the reviewers upon reasonable request. All other extracted data are provided in the online resources.

## Abbreviations

ClinROM: Clinician-reported outcome measure
COSMIN: COnsensus-based Standards for the selection of health Measurement INstruments
EEC: EuroEwing Consortium
FMA: Functional Mobility Assessment
GRADE: Grading of Recommendations Assessment, Development and Evaluation
ICF: International Classification of Functioning, disability and health
ICIDH: International Classification of Impairments, Disabilities and Handicaps
KPS: Karnofsky Performance Status
MSTS: MusculoSkeletal Tumor Society
LE: Lower extremity
LI: Leisure-time Index
PerBOM: Performance-based outcome measure
PROM: Patient-reported outcome measure
PROMIS: Patient-Reported Outcomes Measurement Information System
RNL: Reintegration to Normal Living
SEM: Standard error of measurement
SI: Sports Index
TESS: Toronto Extremity Salvage Score
TI: Total Index
WHO: World Health Organization
WI: Work Index

## Quality ratings

V: Very good methodological quality
A: Adequate methodological quality
D: Doubtful methodological quality
I: Insufficient methodological quality
H: High quality of evidence
M: Moderate quality of evidence
L: Low quality of evidence
VL: Very low quality of evidence
