# Supplementary material for: Critical appraisal of patient-reported and clinician-reported outcome measures assessing functional outcomes in lower extremity bone sarcoma patients—a COSMIN-based systematic review
Source: J Patient Rep Outcomes. 2026 Jun 6;10:142. doi: 10.1186/s41687-026-01099-w (PMC13469031; doi:10.1186/s41687-026-01099-w)
Supplement: Supplementary file 1 [file 41687_2026_1099_MOESM1_ESM.docx]

**Critical appraisal of patient-reported and clinician-reported outcome measures assessing functional outcome in lower extremity bone sarcoma patients—A COSMIN-based systematic review**

**Table of contents**

Online Resource 1.	Search strategies	p. 2-3

Online Resource 2.	Description of measurement properties	p. 4-5

Online Resource 3.	Standards for evaluating the quality of the instrument’s design	p. 6

Online Resource 4.	Description of the criteria for rating content validity by	p. 7

reviewers

Online Resource 5.	A priori hypotheses for rating comparisons between	p. 8

measurement instruments

Online Resource 6.	Modified GRADE framework	p. 9

Online Resource 7.	PRISMA flow diagrams	p. 10-13

Online Resource 8.	Interpretability of measurement instruments	p. 14-23

Online Resource 9.	Study populations of included articles	p. 24-35

Online Resource 10.	Summary of measurement properties results per	p. 36-48

measurement instrument – Extended

Online Resource 11.	Results content validity per study	p. 49-57

Online Resource 12.	Results other measurement properties per study	p. 58-89

[1][2–4]. [5]. [6, 7]. [8, 9] [10].[11] . [12]. [13, 14]. [15, 16]. [17] [18] [16, 19] [9, 20–23], [15, 24], [13, 20, 22, 25–31] [16, 32–43] [16, 33–44] [19, 33, 35–39, 41–43, 45, 46] [14, 47] [48–65] [66, 67] [9, 20–23, 66, 68–72] [15, 24, 73, 74] [13, 20, 22, 25–31, 67, 75–78] [79]. [80]. [81]. [82].[83] [84–86]. [87], [88]

**Online Resource 1**

**Search strategies**

| **MEDLINE** | **#1^*^ AND #2 NOT #3** |
| --- | --- |
| #1 | Baecke[tiab] |
|  | Functional Mobility Assessment[tiab] |
|  | Karnofsky Performance Scale[tiab] OR Karnofsky Performance Score[tiab] OR Karnofsky Performance Status[tiab] OR Karnofsky Performance Index[tiab] OR Karnofsky Performance Rating[tiab] |
|  | Mankin scor*[tiab] OR Mankin's scor*[tiab] OR Mankin scal*[tiab] OR Mankin's scal*[tiab] OR Mankin system*[tiab] OR Mankin's system*[tiab] OR Mankin rating*[tiab] OR Mankin's rating*[tiab] |
|  | (Musculoskeletal Tumor Society[tiab] OR Musculoskeletal Tumour Society[tiab]) AND (scor*[tiab] OR rating*[tiab] OR scale*[tiab] OR system*[tiab]) |
|  | Reintegration to Normal Liv*[tiab] OR Reintegration to Normal Life*[tiab] |
|  | Toronto extremity salvage score[tiab] OR Toronto extremity salvage scale[tiab] |
| #2 [10] | (instrumentation[sh] OR methods[sh] OR “Validation Study”[pt] OR “Comparative Study”[pt] OR “psychometrics”[MeSH] OR psychometr*[tiab] OR clinimetr*[tw] OR clinometr*[tw] OR “outcome assessment, health care”[MeSH] OR “outcome assessment”[tiab] OR “outcome measure*”[tw] OR “observer variation”[MeSH] OR “observer variation”[tiab] OR “Health Status Indicators”[Mesh] OR “reproducibility of results”[MeSH] OR reproducib*[tiab] OR “discriminant analysis”[MeSH] OR reliab*[tiab] OR unreliab*[tiab] OR valid*[tiab] OR “coefficient of variation”[tiab] OR coefficient[tiab] OR homogeneity[tiab] OR homogeneous[tiab] OR “internal consistency”[tiab] OR (cronbach*[tiab] AND (alpha[tiab] OR alphas[tiab])) OR (item[tiab] AND (correlation*[tiab] OR selection*[tiab] OR reduction*[tiab])) OR agreement[tw] OR precision[tw] OR imprecision[tw] OR “precise values”[tw] OR test-retest[tiab] OR (test[tiab] AND retest[tiab]) OR (reliab*[tiab] AND (test[tiab] OR retest[tiab])) OR stability[tiab] OR interrater[tiab] OR inter-rater[tiab] OR intrarater[tiab] OR intra-rater[tiab] OR intertester[tiab] OR inter-tester[tiab] OR intratester[tiab] OR intra-tester[tiab] OR interobserver[tiab] OR inter-observer[tiab] OR intraobserver[tiab] OR intra-observer[tiab] OR intertechnician[tiab] OR inter-technician[tiab] OR intratechnician[tiab] OR intra-technician[tiab] OR interexaminer[tiab] OR inter-examiner[tiab] OR intraexaminer[tiab] OR intra-examiner[tiab] OR interassay[tiab] OR inter-assay[tiab] OR intraassay[tiab] OR intra-assay[tiab] OR interindividual[tiab] OR inter-individual[tiab] OR intraindividual[tiab] OR intra-individual[tiab] OR interparticipant[tiab] OR inter-participant[tiab] OR intraparticipant[tiab] OR intra-participant[tiab] OR kappa[tiab] OR kappa’s[tiab] OR kappas[tiab] OR repeatab*[tw] OR ((replicab*[tw] OR repeated[tw]) AND (measure[tw] OR measures[tw] OR findings[tw] OR result[tw] OR results[tw] OR test[tw] OR tests[tw])) OR generaliza*[tiab] OR generalisa*[tiab] OR concordance[tiab] OR (intraclass[tiab] AND correlation*[tiab]) OR discriminative[tiab] OR “known group”[tiab] OR “factor analysis”[tiab] OR “factor analyses”[tiab] OR “factor structure”[tiab] OR “factor structures”[tiab] OR dimension*[tiab] OR subscale*[tiab] OR (multitrait[tiab] AND scaling[tiab] AND (analysis[tiab] OR analyses[tiab])) OR “item discriminant”[tiab] OR “interscale correlation*”[tiab] OR error[tiab] OR errors[tiab] OR “individual variability”[tiab] OR “interval variability”[tiab] OR “rate variability”[tiab] OR (variability[tiab] AND (analysis[tiab] OR values[tiab])) OR (uncertainty[tiab] AND (measurement[tiab] OR measuring[tiab])) OR “standard error of measurement”[tiab] OR sensitiv*[tiab] OR responsive*[tiab] OR (limit[tiab] AND detection[tiab]) OR “minimal detectable concentration”[tiab] OR interpretab*[tiab] OR ((minimal[tiab] OR minimally[tiab] OR clinical[tiab] OR clinically[tiab]) AND (important[tiab] OR significant[tiab] OR detectable[tiab]) AND (change[tiab] OR difference[tiab])) OR (small*[tiab] AND (real[tiab] OR detectable[tiab]) AND (change[tiab] OR difference[tiab])) OR “meaningful change”[tiab] OR “ceiling effect”[tiab] OR “floor effect”[tiab] OR “Item response model”[tiab] OR IRT[tiab] OR Rasch[tiab] OR “Differential item functioning”[tiab] OR DIF[tiab] OR “computer adaptive testing”[tiab] OR “item bank”[tiab] OR “cross-cultural equivalence”[tiab]) |
| #3 [10] | (“address”[Publication Type] OR “biography”[Publication Type] OR “case reports”[Publication Type] OR “comment”[Publication Type] OR “directory”[Publication Type] OR “editorial”[Publication Type] OR “festschrift”[Publication Type] OR “interview”[Publication Type] OR “lecture”[Publication Type] OR “legal case”[Publication Type] OR “legislation”[Publication Type] OR “letter”[Publication Type] OR “news”[Publication Type] OR “newspaper article”[Publication Type] OR “patient education handout”[Publication Type] OR “popular work”[Publication Type] OR “congress”[Publication Type] OR “consensus development conference”[Publication Type] OR “consensus development conference, nih”[Publication Type] OR “practice guideline”[Publication Type]) NOT (“animals”[MeSH Terms] NOT “humans”[MeSH Terms]) |
| **Embase** | **#1^*^ AND #2 NOT #3** |
| #1 | Baecke:ti,ab,kw |
|  | ‘Functional mobility assessment’:ti,ab,kw |
|  | ‘Karnofsky Performance Scale’:ti,ab,kw OR ‘Karnofsky Performance Score’:ti,ab,kw OR ‘Karnofsky Performance Status’:ti,ab,kw OR ‘Karnofsky Performance Index’:ti,ab,kw OR ‘Karnofsky Performance Rating’:ti,ab,kw |
|  | ('mankin*' NEAR/2 ('scor*' OR 'scal*' OR 'system*' OR 'rating*')):ti,ab,kw |
|  | ((‘Musculoskeletal Tumor Society’ OR ‘Musculoskeletal Tumour Society’) NEAR/4 (‘scor*’ OR ‘rating*’ OR ‘scale*’ OR ‘system*’)):ti,ab,kw |
|  | ‘Reintegration to Normal Liv*’:ti,ab,kw OR ‘Reintegration to Normal Life*’:ti,ab,kw |
|  | ‘Toronto extremity salvage score*’:ti,ab,kw OR ‘Toronto extremity salvage scale*’:ti,ab,kw |
| #2 [10] | ‘intermethod comparison’/exp OR ‘data collection method’/exp OR ‘validation study’/exp OR ‘feasibility study’/exp OR ‘pilot study’/exp OR ‘psychometry’/exp OR ‘reproducibility’/exp OR reproducib*:ab,ti OR ‘audit’:ab,ti OR psychometr*:ab,ti OR clinimetr*:ab,ti OR clinometr*:ab,ti OR ‘observer variation’/exp OR ‘observer variation’:ab,ti OR ‘discriminant analysis’/exp OR ‘validity’/exp OR reliab*:ab,ti OR valid*:ab,ti OR ‘coefficient’:ab,ti OR ‘internal consistency’:ab,ti OR (cronbach*:ab,ti AND (‘alpha’:ab,ti OR ‘alphas’:ab,ti)) OR ‘item correlation’:ab,ti OR ‘item correlations’:ab,ti OR ‘item selection’:ab,ti OR ‘item selections’:ab,ti OR ‘item reduction’:ab,ti OR ‘item reductions’:ab,ti OR ‘agreement’:ab,ti OR ‘precision’:ab,ti OR ‘imprecision’:ab,ti OR ‘precise values’:ab,ti OR ‘test-retest’:ab,ti OR (‘test’:ab,ti AND ‘retest’:ab,ti) OR (reliab*:ab,ti AND (‘test’:ab,ti OR ‘retest’:ab,ti)) OR ‘stability’:ab,ti OR ‘interrater’:ab,ti OR ‘inter-rater’:ab,ti OR ‘intrarater’:ab,ti OR ‘intra-rater’:ab,ti OR ‘intertester’:ab,ti OR ‘inter-tester’:ab,ti OR ‘intratester’:ab,ti OR ‘intratester’:ab,ti OR ‘interobeserver’:ab,ti OR ‘inter-observer’:ab,ti OR ‘intraobserver’:ab,ti OR ‘intraobserver’:ab,ti OR ‘intertechnician’:ab,ti OR ‘inter-technician’:ab,ti OR ‘intratechnician’:ab,ti OR ‘intratechnician’:ab,ti OR ‘interexaminer’:ab,ti OR ‘inter-examiner’:ab,ti OR ‘intraexaminer’:ab,ti OR ‘intraexaminer’:ab,ti OR ‘interassay’:ab,ti OR ‘inter-assay’:ab,ti OR ‘intraassay’:ab,ti OR ‘intra-assay’:ab,ti OR ‘interindividual’:ab,ti OR ‘inter-individual’:ab,ti OR ‘intraindividual’:ab,ti OR ‘intra-individual’:ab,ti OR ‘interparticipant’:ab,ti OR ‘inter-participant’:ab,ti OR ‘intraparticipant’:ab,ti OR ‘intraparticipant’:ab,ti OR ‘kappa’:ab,ti OR ‘kappas’:ab,ti OR ‘coefficient of variation’:ab,ti OR repeatab*:ab,ti OR (replicab*:ab,ti OR ‘repeated’:ab,ti AND (‘measure’:ab,ti OR ‘measures’:ab,ti OR ‘findings’:ab,ti OR ‘result’:ab,ti OR ‘results’:ab,ti OR ‘test’:ab,ti OR ‘tests’:ab,ti)) OR generaliza*:ab,ti OR generalisa*:ab,ti OR ‘concordance’:ab,ti OR (‘intraclass’:ab,ti AND correlation*:ab,ti) OR ‘discriminative’:ab,ti OR ‘known group’:ab,ti OR ‘factor analysis’:ab,ti OR ‘factor analyses’:ab,ti OR ‘factor structure’:ab,ti OR ‘factor structures’:ab,ti OR ‘dimensionality’:ab,ti OR subscale*:ab,ti OR ‘multitrait scaling analysis’:ab,ti OR ‘multitrait scaling analyses’:ab,ti OR ‘item discriminant’:ab,ti OR ‘interscale correlation’:ab,ti OR ‘interscale correlations’:ab,ti OR (‘error’:ab,ti OR ‘errors’:ab,ti AND (measure*:ab,ti OR correlat*:ab,ti OR evaluat*:ab,ti OR ‘accuracy’:ab,ti OR ‘accurate’:ab,ti OR ‘precision’:ab,ti OR ‘mean’:ab,ti)) OR ‘individual variability’:ab,ti OR ‘interval variability’:ab,ti OR ‘rate variability’:ab,ti OR ‘variability analysis’:ab,ti OR (‘uncertainty’:ab,ti AND (‘measurement’:ab,ti OR ‘measuring’:ab,ti)) OR ‘standard error of measurement’:ab,ti OR sensitiv*:ab,ti OR responsive*:ab,ti OR (‘limit’:ab,ti AND ‘detection’:ab,ti) OR ‘minimal detectable concentration’:ab,ti OR interpretab*:ab,ti OR (small*:ab,ti AND (‘real’:ab,ti OR ‘detectable’:ab,ti) AND (‘change’:ab,ti OR ‘difference’:ab,ti)) OR ‘meaningful change’:ab,ti OR ‘minimal important change’:ab,ti OR ‘minimal important difference’:ab,ti OR ‘minimally important change’:ab,ti OR ‘minimally important difference’:ab,ti OR ‘minimal detectable change’:ab,ti OR ‘minimal detectable difference’:ab,ti OR ‘minimally detectable change’:ab,ti OR ‘minimally detectable difference’:ab,ti OR ‘minimal real change’:ab,ti OR ‘minimal real difference’:ab,ti OR ‘minimally real change’:ab,ti OR ‘minimally real difference’:ab,ti OR ‘ceiling effect’:ab,ti OR ‘floor effect’:ab,ti OR ‘item response model’:ab,ti OR ‘irt’:ab,ti OR ‘rasch’:ab,ti OR ‘differential item functioning’:ab,ti OR ‘dif’:ab,ti OR ‘computer adaptive testing’:ab,ti OR ‘item bank’:ab,ti OR ‘cross-cultural equivalence’:ab,ti |
| #3 | ('note':it OR 'editorial':it OR 'letter':it OR 'conference review':it) NOT ('animal'/exp NOT 'human'/exp) |

MEDLINE covers 1946 to present. Embase covers 1947 to present.

^*^Searches were performed separately per measurement instrument.

**Online Resource 2**

**Description of development, measurement properties, and interpretability**

| **Measurement property** | **Description** [7] |
| --- | --- |
| *Development* | The foundation of a good measurement instrument is built at the development stage. This involves defining the construct of interest, generating items that accurately represent the construct, and ensuring these items are appropriate for the target population. Key activities include concept elicitation, item generation, and pilot testing. Input from patients and/or professionals is incorporated to confirm that the items are relevant, clear, and understandable. The goal of the development stage is to establish a draft or final version of the instrument. |
| *Content validity* | Content validity assesses how well the items reflect the construct they are intended to measure. Key aspects include:   - Relevance: The items must relate directly to the construct. - Comprehensiveness: The instrument should cover all important dimensions of the construct. - Comprehensibility: Items must be easy to understand for the target population or reporter.   Content validity studies are typically conducted after the final version of the instrument is established. These studies may be performed by the original developers or independent researchers and often involve new patient or professional samples to assess the quality of the finalized instrument. |
| *Structural validity* | Structural validity is the extent to which the scores of a measurement instrument accurately reflect the dimensional structure of the construct. Essentially, it ensures that the way the items in the instrument are organized and scored makes sense based on the theory behind the construct. |
| *Internal consistency* | Internal consistency reflects the extent to which the items within a measurement instrument are correlated, suggesting that they measure the same underlying construct. Internal consistency is closely related to structural validity. Once structural validity establishes that items form coherent subscales or factors aligned with the construct's theoretical structure, internal consistency assesses how well these items work together within each dimension. Therefore, internal consistency can only be accurately assessed after establishing the instrument’s structural validity. |
| *Cross-cultural validity/ measurement invariance* | Cross-cultural validity/measurement invariance ensures that a measurement instrument functions similarly across different (cultural) groups. It means that the instrument measures the same construct in the same way, regardless of (cultural) differences, allowing for valid comparisons between groups. |
| *Reliability* | Reliability refers to the consistency of the measurement, meaning that an instrument produces stable and repeatable results under consistent conditions. This includes:   - Test-retest reliability: The degree to which the same patient- or clinician-reporter produces consistent scores at different times.   Inter-rater reliability: The degree to which different clinician-reporters produce consistent scores about the same patient at the same time. |
| *Measurement error* | **Measurement error** is the difference between an observed score and the true score, caused by inconsistencies such as environmental factors or random variations, reducing the accuracy and reliability of a measurement. |
| *Criterion validity* | Criterion validity assesses how well a measurement instrument correlates with a "gold standard" that measures the same construct. Strong criterion validity indicates that the instrument accurately assesses the construct it is intended to measure. |
| *Construct validity* | **Construct validity** evaluates whether an instrument accurately measures the theoretical construct it is intended to measure. This is done by examining the instrument’s relationships with other variables based on formulated hypotheses. Key methods include comparing the instrument's outcomes with those from similar tools, or examining differences across relevant subgroups (e.g., by gender, age, or disease severity) as predicted by theory. When these relationships align with expectations, it supports the instrument's construct validity and its effectiveness in capturing the intended construct. |
| *Responsiveness* | Responsiveness comprises the ability of an instrument to detect meaningful changes over time, particularly in response to interventions or changes in the patient's condition. Responsiveness can be assessed through various comparisons, including:   - Comparison to a gold standard: Evaluating how well the instrument reflects changes recognized by an established measure. - Comparison with other instruments: Analyzing whether the instrument can detect changes similarly to other relevant measures. - Comparison between subgroups: Examining differences in responsiveness among different groups, such as age or disease severity.   Before and after intervention: Measuring changes in scores before and after a specific treatment or intervention to assess the instrument's effectiveness in capturing improvements or declines in health status. |
| *Interpretability* | The degree to which one can assign meaningful, clinically or practically relevant interpretations to the results of the measurement instrument. |

**Online Resource 3**

**Standards for evaluating the quality of the instrument’s design** [7]

| *General design requirements* | **Very good (V)** | **Adequate (A)** | **Doubtful (D)** | **Inadequate (I)** |
| --- | --- | --- | --- | --- |
| 1. Is a clear description provided of the construct to be measured? | Construct clearly described |  |  | Construct not clearly described |
| 2. Is the origin of the construct clear: was a theory, conceptual framework or disease model used or clear rationale provided to define the construct to be measured? | Origin of the construct clear |  | Origin of the construct not clear |  |
| 3. Is a clear description provided of the target population for which the PROM was developed? | Target population clearly described |  |  | Target population not clearly described |
| 4. Is a clear description provided of the context of use | Context of use clearly described |  | Context of use not clearly described |  |

**Online Resource 4**

**Description of the criteria for rating content validity by reviewers** [7]

| **Criteria** |  | **Description** [7] |
| --- | --- | --- |
| *Relevance* | 1 | Reviewers consider at least 85% of the items relevant for the construct of interest. |
|  | 2 | Reviewers consider at least 85% of the items relevant for the population of interest. |
|  | 3 | Reviewers consider at least 85% of the items relevant for the context of use of interest. |
|  | 4 | Reviewers consider at least 85% of the response options appropriate for the construct, population, and context of use of interest. |
|  | 5 | Reviewers consider the recall period appropriate for the construct, population, and context of use of interest. |
| *Comprehensiveness* | 1 | Reviewers consider the PROM comprehensive for the construct, population and context of use of interest. |
| *Comprehensibility* | 1 | Reviewers consider at least 85% of the items and response options appropriately worded. |
|  | 2 | Reviewers consider at least 85% of the response options matching the questions. |

Ratings can be sufficient (+), insufficient (‐) or indeterminate (?)

**Online Resource 5**

**A priori hypotheses for rating construct validity and responsiveness when evaluated by comparisons between measurement instruments**

|  | **Relation between constructs** | **Correlation coefficient** |
| --- | --- | --- |
| Target AND comparator instrument (bone) sarcoma specific | Same construct | ≥ 0.7 |
|  | Largely related construct | 0.5 - 0.7 |
|  | Moderately related construct | 0.3 - 0.6 |
|  | Unrelated construct | < 0.30 |
| Target OR comparator instrument (bone) sarcoma specific | Same construct | ≥ 0.6 |
|  | Largely related construct | 0.5 - 0.7 |
|  | Moderately related construct | 0.3 - 0.6 |
|  | Unrelated construct | < 0.30 |
| Target AND comparator instrument NOT (bone) sarcoma specific | Same construct | ≥ 0.6 |
|  | Largely related construct | 0.5 - 0.7 |
|  | Moderately related construct | 0.3 - 0.6 |
|  | Unrelated construct | < 0.30 |

*Note: These thresholds serve as indicative ranges to evaluate whether observed correlations align with the type of relationship hypothesized (e.g., same construct, related construct, unrelated construct). They are not meant to serve as classification rules for assigning relationships based solely on correlation values. Overlap between ranges reflects inherent uncertainty in the subjective process of defining construct relationships.*

**Online Resource 6**

**GRADE approach for grading the quality of evidence according to COSMIN guidelines** [7]

| **Quality of evidence** | **Lower if** |
| --- | --- |
| High | *Risk of bias*  -1 Serious  -2 Very serious  -3 Extremely serious  *Inconsistency*  -1 Serious  -2 Very serious  *Imprecision*  -1 Serious  -2 Very serious  *Indirectness*  -1 Serious  -2 Very serious |
| Moderate |  |
| Low |  |
| Very low |  |

**Downgrading for risk of bias**

| **Risk of bias** | **Downgrading for Risk of Bias** |
| --- | --- |
| No | There are multiple studies of at least adequate quality, or there is one  study of very good quality available |
| Serious | There are multiple studies of doubtful quality available, or there is  only one study of adequate quality |
| Very serious | There are multiple studies of inadequate quality, or there is only one  study of doubtful quality available |
| Extremely serious | There is only one study of inadequate quality available |

**Online Resource 7**

**PRISMA flow diagram Baecke questionnaire**

**PRISMA flow diagram Functional Mobility Assessment**

**PRISMA flow diagram Karnofsky Performance Status**

**PRISMA flow diagram Mankin score**

**PRISMA flow diagram MusculoSkeletal Tumor Society score**

**PRISMA flow diagram Reintegration to Normal Living Index**

**PRISMA flow diagram Toronto Extremity Salvage Score – Lower Extremity**

**Online Resource 8**

**Interpretability of the Baecke questionnaire**

|  | **Distribution of scores in the study population** | **Percentage of missing items and percentage of missing total scores** | **Floor and ceiling effects** | Scores available for relevant (sub)groups and scores over time | **MIC or MID** | **Information on**  **response shift** |
| --- | --- | --- | --- | --- | --- | --- |
| Albanes et al. [45] | TI: mean = 7.5, SD = 0.4 |  |  |  |  |  |
| Baecke et al. [16] |  |  |  | Males vs females - WI: mean = 2.6 vs 2.9, SEM = 0.1 vs 0.0 - SI: mean = 2.8 vs 2.4, SEM = 0.1 vs 0.1 - LI: mean = 2.8 vs 3.1, SEM = 0.1 vs 0.0 |  |  |
| Burns et al. [32] | WI: mean = 2.7, SD = 0.7 LI: mean = 2.7, SD = 0.7 |  |  | Males vs females - WI: mean = 2.8 vs 2.7, SD = 0.7 vs 0.6 - LI: mean = 2.7 vs 2.3, SD = 0.7 vs 0.5 |  |  |
| Carvalho et al. [33] | WI: mean = 2.3, SD = 0.9 SI: mean = 1.8, SD = 0.9 LI: mean = 2.1, SD = 0.6 TI: mean = 6.2, SD = 1.5 |  |  |  |  |  |
| Florindo et al. [44] | SI: mean = 4.0, SD = 0.5, range = 2.8-4.5 LI: mean = 3.4, SD = 0.5, range = 2.3-4.3 |  |  |  |  |  |
| Florindo et al. [35] | WI: mean = 2.6, SD = 0.5 SI: mean = 2.3, SD = 0.7 LI: mean = 2.6, SD = 0.6 TI: mean = 7.3, SD = 1.2 |  |  |  |  |  |
| Jacobs et al. [36] |  |  |  | Males vs females - WI: mean = 1.7 vs 1.8, SD = 0.4 vs 0.4 - SI: mean = 3.1 vs 2.9, SD = 0.9 vs 0.9 - LI: mean = 2.8 vs 2.8, SD = 0.5 vs 0.6 - TI: mean = 7.6 vs 7.5, SD = 1.3 vs 1.5 |  |  |

LI = Leisure-time Index; MIC = minimal important change; MID = minimal important difference; SD = standard deviation; SEM = standard error of the mean; SI = Sports Index; TI = Total Index; WI = Work Index.

**Interpretability of the Functional Mobility Assessment**

|  | **Distribution of scores in the study population** | **Percentage of missing items and percentage of missing total scores** | **Floor and ceiling effects** | Scores available for relevant (sub)groups and scores over time | **MIC or MID** | **Information on response shift** |
| --- | --- | --- | --- | --- | --- | --- |
| Marchese et al. [14] | Lower extremity sarcoma: mean = 48.6, SD = 8.2  Healthy volunteers:  mean = 55.9, SD = 4.3 |  | No floor or ceiling effects | 6 months postop: mean = 38.5, SD = 10.7  12 months postop: mean = 44.7, SD = 7.7  18 months postop: mean = 43.5, SD = 3.9 |  |  |
| Marchese et al. [79] | Healthy participants:  mean = 59, SD = 3 |  |  | Female: mean = 58.8, range = 46-68  Male: mean = 59.7, range = 47-68  Reference values stratified by gender and  age (10-21 years old): see article. |  |  |
| Pierce et al. [47] | Amputation group:  mean = 50.5, SD = 6.0  Control group:  mean = 57.8, SD = 3.9 |  |  |  |  |  |

MIC = minimal important change; MID = minimal important difference; postop = postoperatively; SEM = standard error of the mean; SD = standard deviation.

**Interpretability of the Karnofsky Performance Status**

|  | **Distribution of scores in the study population** | **Percentage of missing items and percentage of missing total scores** | **Floor and ceiling effects** | Scores available for relevant (sub)groups and scores over time | **MIC or MID** | **Information on response shift** |
| --- | --- | --- | --- | --- | --- | --- |
| Celtek et al. [49] | Mean = 64.6, SD = 15.3 |  |  | Male: mean = 66.3, SD = 14.8 |  |  |
| Conill et al. [50] | - |  |  | Female: mean = 62.2, SD = 16.0 |  |  |
| Crooks et al. [51] | Mean = 73, SD = 17 |  |  | - |  |  |
| Grieco et al. [53] | - |  |  | - |  |  |
| Liem et al. [55] | Median = 80, range = 20-100 |  |  | Normal subjects: mean = 99.0 |  |  |
| Stedman et al. [56] | Mean = 78.9, SD = 12, 95%CI = 78.8-79.1 |  |  | Outpatients: mean = 75.0 |  |  |
| Myers et al. [58] |  |  |  | Chronic pain patients: mean = 62.0 |  |  |
| O'Dell et al. [60] | Mean = 88.6, SD = 11.2, range = 40-100 |  |  | Dialysis patients: mean = 51.0 |  |  |
| O'Dell et al. [59] | Mean = 82.0, SD = 9.1, range = 40-100 |  |  | Stroke patients: mean = 39.5 |  |  |
| Roila et al. [61] | Mean = 75.2-79.5^*^, SD = 19.0-21.6^*^ |  |  |  |  |  |
| Schag et al. [62] | Range = 30-100 |  |  | - |  |  |
| Taylor et al. [63] | Mean = 71.7-76.2^*^ |  |  | Outpatient palliative care clinic:  Median = 70, range = 10-100 |  |  |
| Yates et al. [64] | Mean = 69.7-70.2^*^, range = 30-100 |  |  | Inpatient oncology unit:  Median = 50, range = 10-90 |  |  |

MIC = minimal important change; MID = minimal important difference; SEM = standard error of the mean; SD = standard deviation.

^*^Dependent on rater

**Interpretability of the Mankin score**

|  | **Distribution of scores in the study population** | **Percentage of missing items and percentage of missing total scores** | **Floor and ceiling effects** | Scores available for relevant (sub)groups and scores over time | **MIC or MID** | **Information on response shift** |
| --- | --- | --- | --- | --- | --- | --- |
| Mankin et al. [18] | 28% excellent, 46% good, 4% fair, 22% failure |  |  | Osteoarticular graft:  24% excellent, 49% good, 5% fair, 22% failure  Intercalary graft:  50% excellent, 34% good, 2% fair, 13% failure  Allo-prosthesis:  24% excellent, 53% good, 2% fair, 20% failure  Allo-arthrodesis:  3% excellent, 51% good, 6% fair, 41% failure |  |  |

MIC = minimal important change; MID = minimal important difference.

**Interpretability of the MusculoSkeletal Tumor Society 1987 score**

|  | **Distribution of scores in the study population** | **Percentage of missing items and percentage of missing total scores** | **Floor and ceiling effects** | Scores available for relevant (sub)groups and scores over time | **MIC or MID** | **Information on response shift** |
| --- | --- | --- | --- | --- | --- | --- |
| Bolia et al. [67] | Mean = 62.8, SD = 21.5 |  |  |  |  |  |
| Davis et al. [66] |  |  | Floor effect: 2%  Ceiling effect: 33% |  |  |  |

MIC = minimal important change; MID = minimal important difference; SD = standard deviation.

**Interpretability of the MusculoSkeletal Tumor Society 1993 score-Lower extremity**

|  | **Distribution of scores in the study population** | **Percentage of missing items and percentage of missing total scores** | **Floor and ceiling effects** | Scores available for relevant (sub)groups and scores over time | **MIC or MID** | **Information on response shift** |
| --- | --- | --- | --- | --- | --- | --- |
| Davis et al. [66] |  |  | Floor effect: 2% | - | - |  |
| Enneking et al. [9]1993 | Mean = 73% |  | Ceiling effect: 33% | - | - |  |
| Gazendam et al. [87] |  |  |  | Preop:  mean = 56.5, SD = 30.3 | MID = 12 points  (sens = 0.60, spec = 0.76) |  |
| Iwata et al. [68] | Mean = 82, SD = 20.4,  range = 13-100 | 0% | - | 3 months postop:  mean = 60.7, SD = 22.3 | - |  |
| Kask et al. [20] | Mean = 76, SD = 3.1,  median = 80, IQR = 4.7–3.2 |  | Floor effect: 0% | 6 months postop:  mean = 71.9, SD = 21.5 | - |  |
| Mallet et al. [21] | Mean = 21.18, SD = 6.57 | 0% | Ceiling effect: 23% | 12 months postop:  mean = 78.2, SD = 20.0 | - |  |
| Rebolledo et al. [23] | Mean = 71%, SD = 21.8, range = 16.7-100% | 0% | Floor effect: 0% |  | - |  |
| Rizzo et al. [70] | - | 0% | Ceiling effect: 5% |  | - |  |
| Saebye et al. [71] | Median = 87%, IQR = 73-97% | - | Not reported |  | - |  |
| Xu et al. [72] | Mean = 21.5, SD = 7.1,  range = 6-30 | 0% | Floor effect: 0% |  | - |  |

IQR = interquartile range; MIC = minimal important change; MID = minimal important difference; postop = postoperatively; preop = preoperatively; SD = standard deviation; sens = sensitivity; spec = specificity.

**Interpretability of the Reintegration to Normal Living Index**

|  | **Distribution of scores in the study population** | **Percentage of missing items and percentage of missing total scores** | **Floor and ceiling effects** | Scores available for relevant (sub)groups and scores over time | **MIC or MID** | **Information on response shift** |
| --- | --- | --- | --- | --- | --- | --- |
| Merz et al. [74] | Total score: mean = 75.3,  SD = 19.9, range = 20-100 |  |  |  |  |  |
| Okoye et al. [24] | Total score: mean = 58.6,  SD = 21.3 |  |  |  | Total score:  MDD = 5.83  Daily functioning:  MDD = 6.47  Perception of self:  MDD = 5.83 |  |
| Wood-Dauphinee et al. [15]  Wood-Dauphinee et al. [73] | Sample 1*: mean = 90.2,  SD = 22.9  Sample 2 (at discharge)*: mean = 83.9, SD = 14.7  Sample 2 (at 3 months FU)*: mean = 90.4, SD = 73.1  Sample 3*: mean = 87.8,  SD = 17.4, range = 10-100 |  |  | Shortly after hospital discharge:  mean = 83.4, SD = 14.7  3 months after hospital discharge:  mean = 90.4, SD = 13.1 |  |  |

FU = follow-up; MIC = minimal important change; MID = minimal important difference; MMD = minimal detectable difference; postop = postoperatively; preop = preoperatively; SD = standard deviation.

*See Online Resource 4 for study populations

**Interpretability of the Toronto Extremity Salvage Score - Lower extremity**

|  | **Distribution of scores in the study population** | **Percentage of missing items and percentage of missing total scores** | **Floor and ceiling effects** | Scores available for relevant (sub)groups and scores over time | **MIC or MID** | **Information on response shift** |
| --- | --- | --- | --- | --- | --- | --- |
| Bolia et al. [67] | Mean = 69.7, SD = 14.5 |  |  |  |  |  |
| Davis et al. [25] |  |  |  | 6 months postop:  mean = 73.6, SD = 16.7  12 months postop:  mean = 79.2, SD = 15.7 |  |  |
| Davis et al. [13] |  |  |  | Total population-preop:  mean = 81.6, SD = 20.7,  range = 22-100  Total population-6 weeks postop:  mean = 60.9, SD = 20.9,  range = 24-100  Total population-3 months postop:  mean = 75.2, SD = 19.6,  range = 34-100  Total population-6 months postop:  mean = 82.0, SD = 16.7,  range = 30-100  Soft tissue tumor-preop:  mean = 88.6, SD = 17.1,  range = 22-100  Soft tissue tumor-6 weeks postop:  mean = 64.5 SD = 22.3,  range = 24-100  Soft tissue tumor-3 months postop:  mean = 79.6, SD = 20.8,  range = 34-100  Soft tissue tumor-6 months postop:  mean = 83.3, SD = 17.0,  range = 30-100  Bone tumor-preop:  mean = 74.5, SD = 21.6,  range = 24-100  Bone tumor-6 weeks postop:  mean = 57.4, SD = 19.1,  range = 25-100  Bone tumor-3 months postop:  mean = 69.7, SD = 19.2,  range = 38-100  Bone tumor-6 months postop:  mean = 80.8, SD = 16.5,  range = 30-100 |  |  |
| Davis et al. [25] |  |  | Floor effect: 0%  Ceiling effect: 0.2-3.7% | Preop: mean = 78.9, SD = 23.1  6 weeks postop:  mean = 66.5, SD = 23.9  3 months postop:  mean = 73.9, SD = 22.5  6 months postop:  mean = 80.8, SD = 18.8  12 months postop:  mean = 85.5, SD = 15.7 |  |  |
| Gazendam et al. [87] |  |  |  | Preop: mean = 63.6, SD = 27.9  3 months postop:  mean = 67.4, SD = 21.5  6 months postop:  mean = 75.3, SD = 19.4  12 months postop:  mean = 80.9, SD = 17.8 | MID = 11 points  (sens = 0.66, spec = 0.64) |  |
| Janssen et al. [75] | Median = 75, IQR = 51-89,  range = 2.9-100 | Missing items: 7% | Floor effect: 0%  Ceiling effect: 2% |  |  |  |
| Kask et al. [20] | Mean = 88, SD = 12.8,  median = 89, IQR = 99.6-77.8 |  | Floor effect: 0%  Ceiling effect: 26% |  |  |  |
| Kask et al. [76] |  |  | Floor effect: 0%  Ceiling effect: 21% |  |  |  |
| Kim et al. [26] | Mean = 80, SD = 20.3,  range = 30-100 |  |  | Soft tissue sarcoma: mean = 86.2,  SD = 17.9, range = 33-100  Bone sarcoma: mean = 72.9,  SD = 20.7, range = 31-98  Limb salvage surgery: mean = 80.0,  SD = 20.6, range = 31-100 |  |  |
| Ocaktan et al. [22] | Mean = 78.8, SD = 15.8 |  |  |  |  |  |
| Ogura et al. [77] | Mean = 86.7, SD = 16.5,  range = 26.7-100 | Missing items: 0.1% | Floor effect: 0%  Ceiling effect: 16.7% |  |  |  |
| Ogura et al. [88] |  |  |  | 3 months post op: mean = 75.8,  SD = 19.9, range = 19.8-100  6 months post op: mean = 80.5,  SD = 21.4, range = 18.3-100  12 months post op: mean = 84.0,  SD = 17.3, range = 19.8-100 | MCID at 3 vs 6 months postop:  4.4 (by ROC analysis),  4.3 (by AIC analysis) MCID at 3 vs 12 months postop:  10.9 (by ROC analysis), 10.6-11.6 (by AIC analysis) |  |
| Rossi et al. [27] |  | Missing items: 0% |  | Preoperatively:  mean = 79.1, range = 14.8-100  3 months post op:  mean = 71.8, range = 9.5-100  3.5 months post op:  mean = 72.6, range = 20.5-100  6 months post op:  mean = 84.2, range = 26.3-100 |  |  |
| Saraiva et al. [29] | Mean = 89.6, SD = 10.2,  range = 56-100 |  |  | Amputation: mean = 94.4,  SD = 8.4, range = 76-100  Prosthesis: mean = 87.0,  SD = 10.6, range = 56-100 |  |  |
| Srisawat et al. [30] | Mean = 88.0, SD = 14.4 |  |  |  |  |  |
| Trost et al. [31] | Mean = 77.6, SD = 19.5,  median = 81.8, range = 19.4-100 |  |  |  |  |  |
| Willeumier et al. [78] | Mean = 77.5, SD = 19.8;  median = 80.2, range = 13.3-100 |  | Floor effect: 0%  Ceiling effect: 10% |  |  |  |

IQR = interquartile range; MCID = minimal clinically important difference; MIC = minimal important change; MID = minimal important difference; postop = postoperatively; preop = preoperatively; SD = standard deviation; sens = sensitivity; spec = specificity.

**Online Resource 9**

**Study populations of included articles: Baecke questionnaire**

|  | **Population** | | | | **Disease characteristics** | | | **Instrument administration** | | |
| --- | --- | --- | --- | --- | --- | --- | --- | --- | --- | --- |
| **Ref** | **N** | **Subjects** | **Age**  *Mean in years*  *(SD, range)* | **Sex**  *% female* | **Disease** | **Treatment** | **Time since diagnosis / surgery** | **Setting** | **Country** | **Language** |
| Albanes et al. [45] | 21 | Healthy men | Median 36  (28-55) | 0% | N/A | N/A | N/A | Human study facility of a research center | USA | English |
| Arakawa Martins et al. [19] | 25 | Older adults | 66-100 | 68% | N/A | N/A | N/A | Residential aged care facility | Australia | English |
| Baecke et al. [16] | 306 | Inhabitants | 20-32 | 55% | N/A | N/A | N/A | Home | The Netherlands | Dutch |
| Burns et al. [32] | 213 | Patients | 58.4 (10.3) | 14% | Cardiac disease | Coronary artery bypass surgery (part of the patients) | Hospitalized for mean 11.2 (SD 4.5) days | 3 hospitals (inpatient) | USA | English |
| Carvalho et al. [33] | 73 | Adults with chronic low back pain | 37.2  (12.2, 18-60) | 32% | Non-specific chronic low back pain | Physical therapy | Pain ≥3 months duration | Physical therapy clinic | Brazil | Brazilian-Portuguese |
| Florindo et al. [44] | 21 | Male students | 32.6  (3.2, 27-37) | 0% | N/A | N/A | N/A | School | Brazil | Brazilian-Portuguese |
| Florindo et al. [34] | 326 | Healthy men | 62.5  (7.9, 50-69) | 0% | N/A | N/A | N/A | Hospital | Brazil | Brazilian-Portuguese |
| Florindo et al. [35] | 53 | Adults with HIV/AIDS | 37.2  (26.0-49.5) | ? | HIV/AIDS | ? | ? | Outpatient clinic | Brazil | Brazilian-Portuguese |
| Jacobs et al. [36] | 78 | Healhy adults from a local university community | 20-59 Males: 37.2 (10.0) Females: 37.4 (9.7) | 64% | N/A | N/A | N/A | ? (multiple sites) | USA | English |
| Morelhão et al. [46] | 106 | Adults with chronic low back pain | 40  (11.6, 18-60) | 56% | Non-specific chronic low back pain | Physical therapy (group exercise program) | Pain ≥3 months duration | Local outpatient physical therapy clinic | Brazil | Brazilian-Portuguese |
| Ono et al. [37] | 61 | Women with hip disorders | 53.3 (11.3) | 100% | 28 Unilateral osteoarthritis 17 Unilateral total hip arthroplasty 16 Osteoarthritis and total hip arthroplasty (bilaterally affected) | 33 Total hip arthroplasty | >6 months since surgery* | Hospital (outpatient clinic) | Japan | Japanese |
| Philippaerts et al. [38] | 90 | Men | 30, 35, 40 | 0% | N/A | N/A | N/A | Home | Belgium | Dutch |
| Philippaerts et al. [39] | 19 | Healthy men | 40 | 0% | N/A | N/A | N/A | Investigator site & home | Belgium | Dutch |
| Philippaerts et al. [43] | 166 | Healthy men | 40 | 0% | N/A | N/A | N/A | Investigator site | Belgium | Dutch |
| Rocha et al. [40] | 241 | Healthy adults | 27.7 (9.6) | 53.9% | N/A | N/A | N/A | Online | Brazil | Brazilian-Portuguese |
| Sadeghisani et al. [41] | 126 | Healthy adults | Males: 25.2 (4.9) Females: 25.9 (5.8) | 48% | N/A | N/A | N/A | ? | Iran | Persian |
| Tebar et al. [42] | 251 | Healthy adults with distinct educational levels | 42.4 (17.0) Median 42.0 (IQR 27.0-55.0) | 55% | N/A | N/A | N/A | Home | Brazil | Brazilian-Portuguese |

? = unknown; N/A = not applicable; SD = standard deviation.

*For total hip arthroplasty patients

**Study populations of included articles: Functional Mobility Assessment**

|  | **Population** | | | | **Disease characteristics** | | | **Instrument administration** | | |
| --- | --- | --- | --- | --- | --- | --- | --- | --- | --- | --- |
| **Ref** | **N** | **Subjects** | **Age**  *Mean in years*  *(SD, range)* | **Sex**  *% female* | **Disease** | **Treatment** | **Time since diagnosis / surgery** | **Setting** | **Country** | **Language** |
| Marchese et al. [14] | 114 | 1. 94 patients 2. 20 healthy | 1. 20.1  (5.7, 0.4–42.4) 2. 32.3  (9.6, 16–49) | 1. 47.9% 2. 80% | 1. Sarcoma 2. None | 1. Amputation/ rotationplasty/ limb-sparing surgery 2. None | 1. Age at surgery 14.2 (4.0, 5.5–23.3) years 2. N/A | Hospital | USA | English |
| Marchese et al. [79] | 503 | Healthy | 10-21 | 52% | N/A | N/A | N/A | 2 middle schools, 2 high schools, and 1 college | USA | English |
| Pierce et al. [47] | 27 | 1. 25 patients 2. 12 control | 1. 12.36  (3.1 9-19) 2. 10.25  (1.4, 9-13) | 1. 44% 2. 67% | 1. Congenital/trauma/  osteosarcoma/infection /other 2. None | 1. Amputation 2. None | 1. Age at amputation 3.9 (4.2) years 2. N/A | 1. Outdoor adventure camp 2. Researcher's academic institution | USA | English |

N/A = not applicable; SD = standard deviation.

**Study populations of included articles: Karnofsky Performance Status**

|  | **Population** | | | | **Disease characteristics** | | | **Instrument administration** | | |
| --- | --- | --- | --- | --- | --- | --- | --- | --- | --- | --- |
| **Ref** | **N** | **Subjects** | **Age**  *Mean in years*  *(SD, range)* | **Sex**  *% female* | **Disease** | **Treatment** | **Time since diagnosis / surgery** | **Setting** | **Country** | **Language** |
| Borja et al. [48] | 27 | Patients | 57.7 (32-76) | 37% | Cancer | ? | ? | Palliative care and general oncology wards | Canada | English |
| Celtek et al. [49] | 80 | Patients | 61.61  (13.3, 18-90) | 40% | Cancer | Follow-up treatment | ? | Palliative Care Outpatient Clinic | Turkey | Turkish |
| Conill et al. [50] | 100 | Patients | 59 (23-91) | 50% | Cancer | Radiation therapy | ? | Radiotherapy clinic | Spain | ? |
| Crooks et al. [51] | 134 | Elderly veteran patients | 82 | 10% | Variety of diseases | Geriatric care | ? | Geriatric Outpatient Clinic | USA | English |
| Frappaz et al. [52] | 6 | Clinical case vignettes | (25-80) | 17% | Brain tumors | None or surgery | ? | Survey by email | France | ? |
| Grieco et al. [53] | Experiment 1: 30 Experiment 2: 100 | Experiment 1: clinical patients Experiment 2: normal subjects and clinical patients | Experiment 1: mean 45-74 Experiment 2: mean 39-50, (21-71) | Experiment 1: 10% Experiment 2: 34% | Experiment 1: psychiatric outpatients, alcohol rehabilitation inpatients, general medical inpatients, nursing home residents, terminally ill patients  Experiment 2: stroke patients, dialysis patients, chronic pain patients, medical and psychiatric outpatients | Experiment 1+2: ? | Experiment 1+2: ? | Experiment 1+2: ? | USA | English |
| Hutchinson et al. [54] | Group 1: 29 Group 2: 31 | Group 1: Emergency room patients Group 2: Hemodialysis patients | Group 1+2: ? | Group 1+2: ? | Group 1+2: ? | Group 1: ? Group 2: Hemodialysis | Group 1+2: ? | Group 1: Emergency room Group 2: Hemodialysis ward | Canada | English |
| Karnofsky et al. [17] | 35 | Patients | 57 (42-75) | 3% | Cancer | Nitrogen mustards | ? | Hospital | USA | English |
| Liem et al. [55] | 117 | Patients | ? | ? | Cancer | Radiation therapy | ? | Department of radiation oncology | USA | English |
| Mor et al. [57] | 17 | Narrative patient descriptions | ? | ? | ? | ? | ? | ? | USA | English |
| Myers et al. [58] | 134 | Patients | Median = 67 (21-102) | 52% | Malignant and nonmalignant diagnoses | ? | ? | Outpatient palliative care clinic Inpatient oncology unit Inpatient palliative care unit | Canada | English |
| O'Dell et al. [60] | 77 | Patients | 35.6 (8.8) | 0% | HIV seropositive | ? | ? | Private internal medicine practice | USA | English |
| O'Dell et al. [59] | 160 | Patients | 44.8 (8.8, 28-69) | 0% | HIV seropositive | ? | ? | Inpatient and outpatient clinics | USA | English |
| Roila et al. [61] | 209 | Patients | ? | 67% | Cancer | ? | ? | Medical Oncology Division | Italy | ? |
| Schag et al. [62] | 75 | Patients | ? | ? | Cancer | ? | ? | Veterans Administration Medical Center Private medical oncology practice | USA | English |
| Stedman et al. [56] | 8197 | Patients listed for kidney transplantation | ≥18 | 37% | ? | NA | ? | Data system | USA | English |
| Taylor et al. [63] | 100 | Patients | 56.4 (17-87) | 43% | Cancer | Chemotherapy ± radiotherapy, surgery or t.b.d | ? | Oncology ward | Australia | English |
| Wood et al. [65] | 37 | Patients | ? | ? | End-stage cancer | ? | ? | Cancer Center | USA | English |
| Yates et al. [64] | Interrater reliability: 52 Tet-retest reliability: 50 Hypotheses testing: 52 | Patients | Interrater reliability: 55 (22-82) Test-retest reliability: 59 (28-85) Hypotheses testing: 59  (37-85) | Interrater reliability: 44% Test-retest reliability: 50% Hypotheses testing: 58% | Advanced cancer | ? | ? | Cancer Center | USA | English |

? = unknown; SD = standard deviation; t.b.d = to be determined.

**Study populations of included articles: Mankin score**

|  | **Population** | | | | **Disease characteristics** | | | **Instrument administration** | | |
| --- | --- | --- | --- | --- | --- | --- | --- | --- | --- | --- |
| **Ref** | **N** | **Subjects** | **Age**  *Mean in years*  *(SD, range)* | **Sex**  *% female* | **Disease** | **Treatment** | **Time since diagnosis / surgery** | **Setting** | **Country** | **Language** |
| Mankin et al. [18] | 718 | Patients | ? | ? | Malign and benign tumors, and nontumorous conditions | Allograft transplantation | Mean = 6, range = 2-20.4 years | Orthopedic Oncology Service | USA | English |

? = unknown; SD = standard deviation.

**Study populations of included articles: MusculoSkeletal Tumor Society 1987 score**

|  | **Population** | | | | **Disease characteristics** | | | **Instrument administration** | | |
| --- | --- | --- | --- | --- | --- | --- | --- | --- | --- | --- |
| **Ref** | **N** | **Subjects** | **Age**  *Mean in years*  *(SD, range)* | **Sex**  *% female* | **Disease** | **Treatment** | **Time since diagnosis / surgery** | **Setting** | **Country** | **Language** |
| Bolia et al. [67] | 100 | Patients | 43 (19) | 22% | Lower extremity soft tissue or bone sarcoma | Surgery | Mean (SD): 4 (1.2) years after surgery | Postoperative follow-up appointment (outpatient clinic) | Greece | Greek |
| Davis et al. [66] | 97 | Patients | 42.7  (19.8, 13-82) | 44% | Malignant and benign aggressive bone and soft tissue tumors in lower extremity | Limb preservation ± chemotherapy and radiotherapy | Preoperatively, and 6 weeks, 3, 6 and 12 months postoperatively | ? | Canada | English |

? = unknown; SD = standard deviation.

**Study populations of included articles: MusculoSkeletal Tumor Society 1993 score – Lower extremity**

|  | **Population** | | | | **Disease characteristics** | | | **Instrument administration** | | |
| --- | --- | --- | --- | --- | --- | --- | --- | --- | --- | --- |
| **Ref** | **N** | **Subjects** | **Age**  *Mean in years*  *(SD, range)* | **Sex**  *% female* | **Disease** | **Treatment** | **Time since diagnosis / surgery** | **Setting** | **Country** | **Language** |
| Enneking et al. [9] | 60 | Patients | ? | ? | Tumor in lower extremity | Limb sparing surgery | ? | 3 institutions* | USA | English |
| Davis et al. [66] | 97 | Patients | 42.7  (19.8, 13-82) | 44% | Malignant and benign aggressive bone and soft tissue tumors in lower extremity | Limb preservation ± chemotherapy + radiotherapy | Preoperatively, 6 weeks, 3, 6, 12 months postoperatively | ? | Canada | English |
| Gazendam et al. [87] | 535 | Patients | ? | ? | Lower extremity bone tumors | Surgical excision + endoprosthetic reconstruction | Preoperatively, 3, 6, 12 months postoperatively | Multicenter | Multicenter | Multiple |
| Iwata et al. [68] | 100 | Patients | 48.6  (17.9, 14-82) | 53% | Intermediate or malignant bone or soft tissue tumors located in the lower extremity or pelvic girdle | Surgery | Mean (SD, range): 47.3 (50.1, 7–229) months after surgery | Clinic | Japan | Japanese |
| Kask et al. [20] | 19 | Patients | 62.3 (25-90) | 37% | Soft tissue sarcoma in lower limb | Surgery ± chemotherapy or radiotherapy | Mean = 2 years 11 months; median = 1 year 11 months; range = 6 months -12 years 10 months | Outpatient clinic of Comprehensive Cancer Center | Finland | Finnish |
| Lee et al. [69] | 41 | Patients | ? | ? | Malignant musculoskeletal tumors in lower extremity | Surgery | ? | Hospital | Korea | ? |
| Mallet et al. [21] | 1. Face validity study: 4 2. Validation study: 188 | 1+2. Patients | 1+2. (≥15) | 1+2. ? | 1+2. Malignant musculoskeletal tumor | 1+2. Surgery | 1+2. ≥0.5 months after surgery | Outpatient clinic of 3 reference centers specialized in sarcoma management | France | French |
| Ocaktan et al. [22] | 1. Pilot study: 20 2. Validation study: 23 | 1+2. Patients | 1+2. ? | 1+2. ? | 1+2. Bone sarcoma, soft tissue sarcoma, or benign aggressive tumors in the lower extremity | 1. ? 2. Limb sparing surgery | 1+2. ≥21 months after definitive treatment | Outpatient clinic | Turkey | Turkish |
| Rebolledo et al. [23] | 1. Pilot study: 10 2. Validation study: 67 | 1+2. Patients | 1. (≥12)  2. 30 (13.3) | 1. ? 2. 40% | 1. Primary sarcoma of the lower limb 2. Malignant lower extremity bone tumors | 1 + 2. Limb sparing surgery or amputation ± chemotherapy ± radiotherapy | 1. ≥6 months after surgery 2. Mean (SD, range) follow-up: 5 (4.7, 0.5-22) years after surgery | Sarcoma center | Brazil | Brazilian Portuguese |
| Rizzo et al. [70] | 59 | Patients | 32  (17, ≥18) | ? | Bone or soft-tissue localization of neoplastic disease in lower limb | Surgery ± systemic therapy | Mean (SD, range): 40 (20, ≥12) months after surgery | Follow-up visit at outpatient clinic | Italy | Italian |
| Saebye et al. [71] | 162 | Patients | ? | ? | Bone sarcoma, soft tissue sarcoma, or aggressive benign tumors in the lower extremity | Surgery | ? | Outpatient clinic | Denmark | Danish |
| Xu et al. [72] | 98 | Patients | 50.8  (13.4, 18-6) | 40% | Bone or soft-tissue sarcoma in the lower extremity | Neoadjuvant chemotherapy + tumor resection (± amputation, prosthesis, biological reconstruction) | Mean (SD, range): 2.5 (1.4; 1-5) years after surgery | Follow-up visit | China | Mandarin |

? = unknown

*Not specified

**Study populations of included articles: Reintegration to Normal Living Index**

|  | **Population** | | | | **Disease characteristics** | | | **Instrument administration** | | |
| --- | --- | --- | --- | --- | --- | --- | --- | --- | --- | --- |
| **Ref** | **N** | **Subjects** | **Age**  *Mean in years*  *(SD, range)* | **Sex**  *% female* | **Disease** | **Treatment** | **Time since diagnosis / surgery** | **Setting** | **Country** | **Language** |
| Merz et al. [74] | 928 | Patients | 62.5 (14.2, 20-97) | 46% | Mild to severe stroke | ? | 6 months past stroke | Phone interview | USA | English |
| Okoye et al. [24] | 1. Content validity: 30 2. Measurement properties: 102 | Individuals with mobility disability | 1. 47.9 (24.4) 2. 46.9 (20.9) | 1. 40% 2. 38.2% | Amputation, stroke, spinal cord injury, severe osteoarthritis, rheumatoid arthritis, post-polio syndrome, osteogenesis imperfecta, leprosy, neuropathy, fracture, foot drop | ? | 1+2. ≥3 months 2. Median = 96 months | 3 tertiary hospitals, 2 rehabilitation centers | Nigeria | IGBO language, English |
| Wood-Dauphinee et al. [15, 73] | 1. Development: 126 2. Sample 1: 80 3. Sample 2: 70 4. Sample 3: 194 | 1. Patients, relatives of patients, physicians, social workers, physical therapists, occupational therapists, psychologists, clergymen, lay individuals  2. Patients 3. Patients 4. Patients | 1. ? 2. ? 3. Broad age range* 4. Broad age range* | 1. ? 2. ? 3. Equally divided by sex* 4. 68.6% | 1. Cancer, myocardial infarction, other chronic diseases 2. ? 3. Myocardial infarction, cancer 4. Cancer: lung, breast, other | ? | 1. ? 2. ? 3. Shortly after discharge from hospital & after 3 months 4. 12 months after diagnosis | 1. ? 2. 2 acute-care hospitals, 1 rehabilitation center 3. ? 4. Hospital | Canada | English, French |

? = unknown; SD = standard deviation.

*Not specified

**Study populations of included articles: Toronto Extremity Salvage Score**

|  | **Population** | | | | **Disease characteristics** | | | **Instrument administration** | | |
| --- | --- | --- | --- | --- | --- | --- | --- | --- | --- | --- |
| **Ref** | **N** | **Subjects** | **Age**  *Mean in years*  *(SD, range)* | **Sex**  *% female* | **Disease** | **Treatment** | **Time since diagnosis / surgery** | **Setting** | **Country** | **Language** |
| Bolia et al. [67] | 100 | Patients | 43 (19) | 22% | Bone or soft tissue sarcoma | Surgery | Mean (SD): 4 (1.2) years after surgery | Outpatient clinic or by phone | Greece | Greek |
| Davis et al. [25] | 31 | Patients | ? | ? | Benign and malignant bone and soft tissue tumors | ? | ? | ? | Canada | English |
| Davis et al. [13] | 1. Sample 1: 66 2. Sample 2: 101 | Patients | 1. 41  (16, 13-75) 2. 40 (16) | 1. 47% 2. 50% | Benign and malignant bone and soft tissue tumors | Limb sparing surgery ± radiation ± chemotherapy | 1. Mean (SD, range): 29 (16, 7-69) months after surgery 2. Similar to sample 1 | 1. Email 2. ? | Canada | English |
| Davis et al. [25] | 1. Development study: 94 2. Validation study: 97 | Patients | 42.7  (19.8, 13-82) | 45.3% | Primary malignant or benign aggressive tumor | Limb sparing surgery ± adjuvant irradiation, chemotherapy | Preoperatively, 6 weeks, 3 months, 6 months, 12 months postoperatively | Outpatient clinic | Canada | English |
| Gazendam et al. [87] | 551 | Patients | ? | ? | Bone tumors | Surgical excision + endoprosthetic reconstruction | Preoperatively, 3, 6, 12 months postoperatively | Multicenter | Multicenter | Multiple |
| Janssen et al. [75] | 100 | Patients | Median (IQR): 63 (54-70) | 59% | Bone metastases and primary bone tumors | Surgery ± radiotherapy | ? | Outpatient clinic | USA | English |
| Kask et al. [20] | 19 | Patients | 62.3 (25-90) | 37% | Soft tissue sarcoma | Surgery ± chemotherapy ± radiotherapy | Mean (range): 2.9 (0.5-12.9); median 1.9 years after surgery | Email | Finland | Finnish |
| Kask et al. [76] | 136 | Patients | 65.6 (14.4) | 51% | Soft tissue tumors | Surgery ± radiotherapy | Median (range): 5.1 (0.5-16.4) years after surgery | Email | Finland | Finnish |
| Kim et al. [26] | 92 | Patients | 46.7 (18.4) | 41% | Bone or soft tissue sarcoma | Limb-salvage surgery, amputation | Mean (SD): 46.3 (40.1) months after surgery | Outpatient clinic | Korea | Korean |
| Ocaktan et al. [22] | 23 | Patients | ? | ? | Malignant bone and soft tissue sarcoma or benign aggressive bone tumor | Limb-salvage surgery | ≥18 months after surgery | Outpatient clinic | Turkey | Turkish |
| Ogura et al. [77] | 102 | Patients | 48.3  (17.8, 14-8) | 52% | Intermediate or malignant bone or soft tissue tumors | Limb-salvage, ablative surgery | Mean (SD, range): 54.0 (51.3, 7-229) months after surgery | Outpatient clinic | Japan | Japanese |
| Ogura et al. [88] | 85 | Patients | 53.1  (17.8, 12-8) | 41% | Intermediate or malignant bone or soft tissue tumors | Limb-salvage surgery, amputation | 3 months, 6 months, 12 months postop | Outpatient clinic | Japan | Japanese |
| Rossi et al. [27] | 1. Cognitive debriefing: 30 2. Validation: 63 | Patients | 1. Older than 18 years 2. Older than 18 years | 1. ? 2. ? | Primary malignant or benign aggressive tumor of bone and soft tissue | Limb-salvage surgery ± chemotherapy ± radiotherapy | 1. ≥12 months after surgery 2. Preoperatively, 3, 3.5, 6 months postoperatively | 1. Outpatient clinic 2. Outpatient clinic | Italy | Italian |
| Saebye et al. [28] | 16 | Patients | ? | ? | Sarcoma or aggressive benign tumor | Limb-sparing surgery | ? | Hospital | Denmark | Danish |
| Saraiva et al. [29] | 48 | Patients | 24 | 48% | Osteosarcoma | Amputation, limb- salvage surgery + chemotherapy | Median (range): 10 (1-23) years after surgery | Hospital or email | Brazil | Brazilian-Portuguese |
| Srisawat et al. [30] | 70 | Patients | 40.1 (19.1) | 57.1% | Primary bone and soft tissue sarcoma | Amputation, limb- salvage surgery | ≥12 months after surgery | Outpatient clinic | Thailand | Thai |
| Trost et al. [31] | 32 | Patients | 18 years and older | ? | Sarcoma | Limb-salvage surgery | ≥3 months after surgery | Outpatient clinic | Germany | German |
| Willeumier et al. [78] | 50 | Patients | 48.9  (18.6-74.9) | 53% | Aggressive benign or malignant bone or soft tissue tumors | Limb salvage surgery | Mean (range): 3.5 (0.03-18.8) years after surgery | Outpatient clinic | The Netherlands | Dutch |

? = unknown; IQR = interquartile range; SD = standard deviation.

**Online Resource 10**

**Summary of measurement properties results: Baecke questionnaire – Work Index**

|  | **SUMMARY /**  **POOLED RESULT** | **OVERALL RATING** | **QUALITY OF EVIDENCE** |
| --- | --- | --- | --- |
| Content validity | - | Indeterminate | No level of evidence, rating is indeterminate |
| Relevance | - | Indeterminate | No level of evidence, rating is indeterminate |
| Comprehensiveness | - | Sufficient | Very low: result based on reviewers' rating |
| Comprehensibility | - | Sufficient | Very low: result based on reviewers' rating |
| Structural validity | Structural validity confirmed | Sufficient | Moderate: one very good study,  studies not in (bone) sarcoma population |
| Internal consistency | Cronbach's α = 0.52;  n = 326 | Insufficient | Moderate: one very good study,  study not in (bone) sarcoma population^a^ |
| Cross-cultural validity/ Measurement invariance | - | - | - |
| Reliability |  |  |  |
| a = Test-retest reliability | a. ICC = 0.84-0.95; n = 224 | a. Sufficient | a. Low: one adequate study,  studies not in (bone) sarcoma population^a,b^ |
| b = Inter-rater reliability | - | - | - |
| Measurement error | - | - | - |
| Criterion validity | - | - | - |
| Hypotheses testing for construct validity |  |  |  |
| a = Comparison with other instruments | a. 15 out of 20 hypotheses confirmed; n = 999 | a. Sufficient | a. Moderate: multiple very good studies,  studies not in (bone) sarcoma population |
| b = Comparison between subgroups | b. 3 out of 3 hypotheses confirmed; n = 60 | b. Sufficient | b. Very low: one doubtful study, n = 60,  studies not in (bone) sarcoma population^a^ |
| Responsiveness |  |  |  |
| a = Comparison to gold standard | - | - | - |
| b = Comparison with other instruments | - | - | - |
| c = Comparison between subgroups | - | - | - |
| d = Before and after intervention | - | - | - |

^a^ Results of inadequate study/studies were ignored.

^b^ Results of indeterminate study/studies were ignored.

ICC = intraclass correlation coefficient.

**Summary of measurement properties results: Baecke questionnaire – Sports Index**

|  | **SUMMARY /**  **POOLED RESULT** | **OVERALL RATING** | **QUALITY OF EVIDENCE** |
| --- | --- | --- | --- |
| Content validity | - | Indeterminate | No level of evidence, rating is indeterminate |
| Relevance | - | Indeterminate | No level of evidence, rating is indeterminate |
| Comprehensiveness | - | Sufficient | Very low: result based on reviewers' rating |
| Comprehensibility | - | Sufficient | Very low: result based on reviewers' rating |
| Structural validity | Structural validity confirmed | Sufficient | Moderate: one very good study,  studies not in (bone) sarcoma population |
| Internal consistency | Cronbach's α = 0.52;  n = 326 | Insufficient | Moderate: one very good study,  study not in (bone) sarcoma population^a^ |
| Cross-cultural validity/ Measurement invariance | - | - | - |
| Reliability |  |  |  |
| a = Test-retest reliability | a. ICC = 0.69-0.93; n = 245 | a. Sufficient | a. Low: one adequate study,  studies not in (bone) sarcoma population^a,b^ |
| b = Inter-rater reliability | - | - | - |
| Measurement error | - | - | - |
| Criterion validity | - | - | - |
| Hypotheses testing for construct validity |  |  |  |
| a = Comparison with other instruments | a. 13 out of 20 hypotheses confirmed; n = 1061 | a. Inconsistent | a. Moderate: multiple very good studies, studies not in bone sarcoma population^a^ |
| b = Comparison between subgroups | b. 3 out of 3 hypotheses confirmed; n = 60 | b. Sufficient | b. Very low: one doubtful study, n = 60, studies not in bone sarcoma population^a^ |
| Responsiveness |  |  |  |
| a = Comparison to gold standard | - | - | - |
| b = Comparison with other instruments | - | - | - |
| c = Comparison between subgroups | - | - | - |
| d = Before and after intervention | - | - | - |

^a^ Results of inadequate study/studies were ignored.

^b^ Results of indeterminate study/studies were ignored.

ICC = intraclass correlation coefficient.

**Summary of measurement properties results: Baecke questionnaire – Leisure-time Index**

|  | **SUMMARY /**  **POOLED RESULT** | **OVERALL RATING** | **QUALITY OF EVIDENCE** |
| --- | --- | --- | --- |
| Content validity | - | Indeterminate | No level of evidence, rating is indeterminate |
| Relevance | - | Indeterminate | No level of evidence, rating is indeterminate |
| Comprehensiveness | - | Insufficient | Very low: result based on reviewers' rating |
| Comprehensibility | - | Sufficient | Very low: result based on reviewers' rating |
| Structural validity | Structural validity confirmed | Sufficient | Moderate: one very good study,  studies not in (bone) sarcoma population |
| Internal consistency | Cronbach's α = 0.62;  n = 326 | Insufficient | Moderate: one very good study,  study not in (bone) sarcoma population^a^ |
| Cross-cultural validity/ Measurement invariance | - | - | - |
| Reliability |  |  |  |
| a = Test-retest reliability | *General population:* |  |  |
|  | a. ICC = 0.77-0.87; n = 143 | a. Sufficient | a. Low: one adequate study,  studies not in bone sarcoma population^a,b^ |
|  | *Chronic low back pain:* |  |  |
|  | a. ICC = 0.61; n = 73 | a. Insufficient | a. Very low: one doubtful study, n = 73,  study not in bone sarcoma population |
|  | *Women with hip disorders:* |  |  |
|  | a. ICC = 0.78; n = 52 | a. Sufficient | a. Very low: one inadequate study, n = 52,  study not in bone sarcoma population |
|  | *Adults with HIV/AIDS:* |  |  |
|  | a. ICC = 0.44; n = 29 | a. Insufficient | a. Very low: one doubtful study, n = 29,  study not in bone sarcoma population |
| b = Inter-rater reliability | - | - | - |
| Measurement error | - | - | - |
| Criterion validity | - | - | - |
| Hypotheses testing for construct validity |  |  |  |
| a = Comparison with other instruments | a. 8 out of 20 hypotheses confirmed; n = 1061 | a. Inconsistent | a. Moderate: multiple very good studies, studies not in bone sarcoma population^a^ |
| b = Comparison between subgroups | b. 3 out of 3 hypotheses confirmed; n = 60 | b. Sufficient | b. Very low: one doubtful study, n = 60, studies not in bone sarcoma population^a^ |
| Responsiveness |  |  |  |
| a = Comparison to gold standard | - | - | - |
| b = Comparison with other instruments | - | - | - |
| c = Comparison between subgroups | - | - | - |
| d = Before and after intervention | - | - | - |

^a^ Results of inadequate study/studies were ignored.

^b^ Results of indeterminate study/studies were ignored.

ICC = intraclass correlation coefficient.

**Summary of measurement properties results: Baecke questionnaire – Total index**

|  | **SUMMARY /**  **POOLED RESULT** | **OVERALL RATING** | **QUALITY OF EVIDENCE** |
| --- | --- | --- | --- |
| Content validity | - | Indeterminate | No level of evidence, rating is indeterminate |
| Relevance | - | Indeterminate | No level of evidence, rating is indeterminate |
| Comprehensiveness | - | Insufficient | Very low: result based on reviewers' rating |
| Comprehensibility | - | Sufficient | Very low: result based on reviewers' rating |
| Structural validity | - | - | - |
| Internal consistency | - | - | ^-^ |
| Cross-cultural validity/ Measurement invariance | - | - | - |
| Reliability |  |  |  |
| a = Test-retest reliability | a. ICC = 0.72-0.88; n = 274 | a. Sufficient | a. Low: one adequate study,  studies not in bone sarcoma population^a,b^ |
| b = Inter-rater reliability | - | - | - |
| Measurement error | - | - | - |
| Criterion validity | - | - | - |
| Hypotheses testing for construct validity |  |  |  |
| a = Comparison with other instruments | a. 16 out of 31 hypotheses confirmed; n = 1494 | a. Inconsistent | a. Moderate: multiple very good studies,  studies not in bone sarcoma population^a^ |
| b = Comparison between subgroups | b. 3 out of 3 hypotheses confirmed; n = 60 | b. Sufficient | b. Very low: one doubtful study, n = 60,  studies not in bone sarcoma population^a^ |
| Responsiveness |  |  |  |
| a = Comparison to gold standard | - | - | - |
| b = Comparison with other instruments | - | - | - |
| c = Comparison between subgroups | - | - | - |
| d = Before and after intervention | d. 0 out of 1 hypotheses confirmed; n = 106 | d. Insufficient | d. Moderate: one very good study,  study not in bone sarcoma population |

^a^ Results of inadequate study/studies were ignored.

^b^ Results of indeterminate study/studies were ignored.

ICC = intraclass correlation coefficient.

**Summary of measurement properties results: Functional Mobility Assessment**

|  | **SUMMARY /**  **POOLED RESULT** | **OVERALL RATING** | **QUALITY OF EVIDENCE** |
| --- | --- | --- | --- |
| Content validity | - | Indeterminate | No level of evidence, rating is indeterminate |
| Relevance | - | Indeterminate | No level of evidence, rating is indeterminate |
| Comprehensiveness | - | Indeterminate | No level of evidence, rating is indeterminate |
| Comprehensibility | - | Inconsistent | Very low: result based on reviewers' rating |
| Structural validity | - | - | - |
| Internal consistency | Cronbach’s α = 0.75;  n = 23 | Indeterminate | No level of evidence, rating is indeterminate |
| Cross-cultural validity/ Measurement invariance | - | - | - |
| Reliability |  |  |  |
| a = Test-retest reliability | - | - | - |
| b = Inter-rater reliability | b. ICC ≥ 0.97; n = 23 | b. Sufficient | b. Very low: one inadequate study |
| Measurement error | - | - | - |
| Criterion validity | - | - | - |
| Hypotheses testing for construct validity |  |  |  |
| a = Comparison with other instruments | a. 3 out of 4 hypotheses confirmed; n = 188 | a. Sufficient | a. Moderate: one adequate study^a^ |
| b = Comparison between subgroups | b. 2 out of 2 hypotheses confirmed; n = 151 | b. Sufficient | b. High: one very good study |
| Responsiveness |  |  |  |
| a = Comparison to gold standard | - | - | - |
| b = Comparison with other instruments | - | - | - |
| c = Comparison between subgroups | - | - | - |
| d = Before and after intervention | d. No hypotheses defined; n = 6 | d. Indeterminate | d. No level of evidence, rating is indeterminate |

^a^ Results of inadequate study/studies were ignored.

ICC = intraclass correlation coefficient.

**Summary of measurement properties results: Karnofsky Performance Status**

|  | **SUMMARY /**  **POOLED RESULT** | **OVERALL RATING** | **QUALITY OF EVIDENCE** |
| --- | --- | --- | --- |
| Content validity | - | Indeterminate | No level of evidence, rating is indeterminate |
| Relevance | - | Indeterminate | No level of evidence, rating is indeterminate |
| Comprehensiveness | - | Insufficient | Very low: result based on reviewers’ rating |
| Comprehensibility | - | Insufficient | Very low: result based on reviewers’ rating |
| Structural validity | - | - | - |
| Internal consistency | - | - | - |
| Cross-cultural validity/ Measurement invariance | - | - | - |
| Reliability |  |  |  |
| a = Test-retest reliability | a. κ = 0.959-0.993; n = 209 | a. Sufficient | a. Low: one doubtful study^a^ |
| b = Inter-rater reliability | *Oncologist vs oncologist* |  |  |
|  | b1. κ = 0.921; n = 209 | b1. Sufficient | b1. Moderate: one adequate study^a,b^ |
|  | *Raters with distinct medical backgrounds* | | |
|  | b2. κ = 0.58-0.71; n = 134 | b2. Inconsistent | b2. Moderate: one adequate study^a,b^ |
| Measurement error | - | - | - |
| Criterion validity | - | - | - |
| Hypotheses testing for construct validity |  |  |  |
| a = Comparison with other instruments | a. 12 out of 17 hypotheses confirmed; n = 1936 | a. Inconsistent | a. High: multiple very good studies^a^ |
| b = Comparison between subgroups | b. 6 out of 6 hypotheses confirmed; n = 314 | b. Sufficient | b. High: one very good study^a,b^ |
| Responsiveness |  |  |  |
| a = Comparison to gold standard | - | - | - |
| b = Comparison with other instruments | - | - | - |
| c = Comparison between subgroups | - | - | - |
| d = Before and after intervention | - | - | - |

^a^ Results of inadequate study/studies were ignored.

^b^ Results of indeterminate study/studies were ignored.

ICC = intraclass correlation coefficient.

**Summary of measurement properties results: Mankin score**

|  | **SUMMARY /**  **POOLED RESULT** | **OVERALL RATING** | **QUALITY OF EVIDENCE** |
| --- | --- | --- | --- |
| Content validity | - | Indeterminate | No level of evidence, rating is indeterminate |
| Relevance | - | Indeterminate | No level of evidence, rating is indeterminate |
| Comprehensiveness | - | Insufficient | Very low: result based on reviewers' rating |
| Comprehensibility | - | Insufficient | Very low: result based on reviewers' rating |
| Structural validity | - | - | - |
| Internal consistency | - | - | - |
| Cross-cultural validity/ Measurement invariance | - | - | - |
| Reliability |  |  |  |
| a = Test-retest reliability | - | - | - |
| b = Inter-rater reliability |  |  |  |
| Measurement error | - | - | - |
| Criterion validity | - | - | - |
| Hypotheses testing for construct validity |  |  |  |
| a = Comparison with other instruments | - | - | ^-^ |
| b = Comparison between subgroups | - | - | - |
| Responsiveness |  |  |  |
| a = Comparison to gold standard | - | - | - |
| b = Comparison with other instruments | - | - | - |
| c = Comparison between subgroups | - | - | - |
| d = Before and after intervention | - | - | - |

**Summary of measurement properties results: MusculoSkeletal Tumor Society 1987 score**

|  | **SUMMARY /**  **POOLED RESULT** | **OVERALL RATING** | **QUALITY OF EVIDENCE** |
| --- | --- | --- | --- |
| Content validity | - | Indeterminate | No level of evidence, rating is indeterminate |
| Relevance | - | Indeterminate | No level of evidence, rating is indeterminate |
| Comprehensiveness | - | Indeterminate | No level of evidence, rating is indeterminate |
| Comprehensibility | - | Insufficient | Very low: result based on reviewers’ rating |
| Structural validity | Unidimensionality unconfirmed | Indeterminate | No level of evidence, rating is indeterminate |
| Internal consistency | Cronbach's α = 0.76-0.81; n = 197 | Indeterminate | No level of evidence, rating is indeterminate |
| Cross-cultural validity/ Measurement invariance | - | - | - |
| Reliability |  |  |  |
| a = Test-retest reliability | a. ICC = 0.997; n = 100 | a. Sufficient | a. Moderate: one adequate study |
| b = Inter-rater reliability | - | - | - |
| Measurement error | SEM = 6.4 - 9.3; n = 97 | Indeterminate | No level of evidence, rating is indeterminate |
| Criterion validity | - | - | - |
| Hypotheses testing for construct validity |  |  |  |
| a = Comparison with other instruments | a. 2 out of 2 hypotheses confirmed; n = 197 | a. Sufficient | a. Moderate: one adequate study |
| b = Comparison between subgroups | - | - | - |
| Responsiveness |  |  |  |
| a = Comparison to gold standard | - | - | - |
| b = Comparison with other instruments | - | - | - |
| c = Comparison between subgroups | - | - | - |
| d = Before and after intervention | d. Data provided uninterpretable | d. Indeterminate | d. No level of evidence, rating is indeterminate |

ICC = intraclass correlation coefficient; SEM = standard error of measurement.

**Summary of measurement properties results: MusculoSkeletal Tumor Society 1993 score – Lower extremity**

|  | **SUMMARY /**  **POOLED RESULT** | **OVERALL RATING** | **QUALITY OF EVIDENCE** |
| --- | --- | --- | --- |
| Content validity | - | Indeterminate | No level of evidence, rating is indeterminate |
| Relevance | - | Indeterminate | No level of evidence, rating is indeterminate |
| Comprehensiveness | - | Indeterminate | No level of evidence, rating is indeterminate |
| Comprehensibility | - | Sufficient^c^ | Moderate: multiple doubtful studies |
| Structural validity | Unidimensionality confirmed | Sufficient | High: one very good study^a,b^ |
| Internal consistency | Cronbach's α = 0.79-0.98; n = 771 | Sufficient | High: multiple very good studies |
| Cross-cultural validity/ Measurement invariance | - | - | - |
| Reliability |  |  |  |
| a = Test-retest reliability | a. ICC = 0.91-0.98; n = 257 | a. Sufficient | a. Moderate: one adequate study^a^ |
| b = Inter-rater reliability | b. ICC = 0.88-0.98; n = 332 | b. Sufficient | b. High: multiple adequate studies |
| Measurement error | SEM = 2.5 - 3.1; n = 97 | Indeterminate | No level of evidence, rating is indeterminate |
| Criterion validity | - | - | - |
| Hypotheses testing for construct validity |  |  |  |
| a = Comparison with other instruments | a. 12 out of 20 hypotheses confirmed; n = 1037 | a. Inconsistent | a. High: multiple very good studies |
| b = Comparison between subgroups | b. 1 out of 1 hypothesis confirmed; n = 98 | b. Sufficient | b. Very low: one doubtful study; n = 98 |
| Responsiveness |  |  |  |
| a = Comparison to gold standard | - | - | - |
| b = Comparison with other instruments | - | - | - |
| c = Comparison between subgroups | - | - | - |
| d = Before and after intervention | d. Data provided uninterpretable | d. Indeterminate | d. No level of evidence, rating is indeterminate |

^a^ Results of inadequate study/studies were ignored.

^b^ Results of indeterminate study/studies were ignored.

^c^ Brazilian Portuguese & Finnish translations.

ICC = intraclass correlation coefficient; SEM = standard error of measurement.

**Summary of measurement properties results: Reintegration to Normal Living index**

|  | **SUMMARY /**  **POOLED RESULT** | **OVERALL RATING** | **QUALITY OF EVIDENCE** |
| --- | --- | --- | --- |
| Content validity | - | Sufficient | Low: one doubtful study |
| Relevance | - | Sufficient | Low: one doubtful study |
| Comprehensiveness | - | Sufficient | Low: one doubtful study |
| Comprehensibility | - | Sufficient | Low: one doubtful study |
| Structural validity | NA | NA | NA |
| Internal consistency | NA | NA | NA |
| Cross-cultural validity/ Measurement invariance | - | - | - |
| Reliability |  |  |  |
| a = Test-retest reliability | - | - | - |
| b = Inter-rater reliability |  |  |  |
| Measurement error | - | - | - |
| Criterion validity | Criterion validity confirmed | Sufficient | High: one very good study |
| Hypotheses testing for construct validity |  |  |  |
| a = Comparison with other instruments | a. 6 out of 12 hypotheses confirmed; n = 2854 | a. Inconsistent | a. Moderate: multiple adequate studies, study not in (bone) sarcoma population^a^ |
| b = Comparison between subgroups | - | - | - |
| Responsiveness |  |  |  |
| a = Comparison to gold standard | - | - | - |
| b = Comparison with other instruments | - | - | - |
| c = Comparison between subgroups | - | - | - |
| d = Before and after intervention | d. No hypothesis defined | d. Indeterminate | d. No level of evidence, rating is indeterminate |

^a^ Results of inadequate study/studies were ignored.

**Summary of measurement properties results: Reintegration to Normal Living index – Daily Functioning**

|  | **SUMMARY /**  **POOLED RESULT** | **OVERALL RATING** | **QUALITY OF EVIDENCE** |
| --- | --- | --- | --- |
| Content validity | NA | NA | NA |
| Relevance | NA | NA | NA |
| Comprehensiveness | NA | NA | NA |
| Comprehensibility | NA | NA | NA |
| Structural validity | NA | NA | NA |
| Internal consistency | NA | NA | NA |
| Cross-cultural validity/ Measurement invariance | - | - | - |
| Reliability |  |  |  |
| a = Test-retest reliability | - | - | - |
| b = Inter-rater reliability |  |  |  |
| Measurement error | - | - | - |
| Criterion validity | Criterion validity confirmed | Sufficient | High: one very good study |
| Hypotheses testing for construct validity |  |  |  |
| a = Comparison with other instruments | a. 2 out of 2 hypotheses confirmed; n = 70 | a. Sufficient | a. Low: one adequate study, n = 70 |
| b = Comparison between subgroups | - | - | - |
| Responsiveness |  |  |  |
| a = Comparison to gold standard | - | - | - |
| b = Comparison with other instruments | - | - | - |
| c = Comparison between subgroups | - | - | - |
| d = Before and after intervention | d. No hypothesis defined | d. Indeterminate | d. No level of evidence, rating is indeterminate |

**Summary of measurement properties results: Reintegration to Normal Living index – Perception of Self**

|  | **SUMMARY /**  **POOLED RESULT** | **OVERALL RATING** | **QUALITY OF EVIDENCE** |
| --- | --- | --- | --- |
| Content validity | NA | NA | NA |
| Relevance | NA | NA | NA |
| Comprehensiveness | NA | NA | NA |
| Comprehensibility | NA | NA | NA |
| Structural validity | NA | NA | NA |
| Internal consistency | NA | NA | NA |
| Cross-cultural validity/ Measurement invariance | - | - | - |
| Reliability |  |  |  |
| a = Test-retest reliability | - | - | - |
| b = Inter-rater reliability |  |  |  |
| Measurement error | - | - | - |
| Criterion validity | Criterion validity confirmed | Sufficient | High: one very good study |
| Hypotheses testing for construct validity |  |  |  |
| a = Comparison with other instruments | a. 2 out of 2 hypotheses confirmed; n = 70 | a. Sufficient | a. Low: one adequate study, n = 70 |
| b = Comparison between subgroups | - | - | - |
| Responsiveness |  |  |  |
| a = Comparison to gold standard | - | - | - |
| b = Comparison with other instruments | - | - | - |
| c = Comparison between subgroups | - | - | - |
| d = Before and after intervention | d. No hypothesis defined | d. Indeterminate | d. No level of evidence, rating is indeterminate |

**Summary of measurement properties results: Toronto Extremity Salvage Score**

|  | **SUMMARY /**  **POOLED RESULT** | **OVERALL RATING** | **QUALITY OF EVIDENCE** |
| --- | --- | --- | --- |
| Content validity | - | Sufficient | Moderate: multiple doubtful studies |
| Relevance | - | Sufficient | Very low: doubtful concept elicitation study |
| Comprehensiveness | - | Sufficient | Moderate: multiple doubtful studies |
| Comprehensibility | - | Sufficient | Moderate: multiple doubtful studies |
| Structural validity | Unidimensionality unconfirmed | Inconsistent | Very low: multiple inadequate studies^b^ |
| Internal consistency | Cronbach's α = 0.87-0.981; n = 467 | Indeterminate | No level of evidence, rating is indeterminate |
| Cross-cultural validity/ Measurement invariance | Age and BMI not related to TESS score | Indeterminate | No level of evidence, rating is indeterminate |
| Reliability |  |  |  |
| a = Test-retest reliability | a. ICC = 0.810-1; n = 437 | a. Sufficient | a. High: multiple adequate studies^a^ |
| b = Inter-rater reliability | - | - | - |
| Measurement error | SEM = 1.68; n = 63 | Sufficient | Low: one adequate study; n=63^a^ |
| Criterion validity | - | - | - |
| Hypotheses testing for construct validity |  |  |  |
| a = Comparison with other instruments | a. 21 out of 53 hypotheses confirmed; n = 1207 | a. Inconsistent | a. High: one very good study^a^ |
| b = Comparison between subgroups | b. 3 out of 3 hypotheses confirmed; n = 246 | b. Sufficient | b. Moderate: multiple doubtful studies^a^ |
| Responsiveness |  |  |  |
| a = Comparison to gold standard | - | - | - |
| b = Comparison with other instruments | - | - | - |
| c = Comparison between subgroups | - | - | - |
| d = Before and after intervention | d. 7 out of 10 hypotheses confirmed; n = 261 | d. Inconsistent | d. Moderate: one adequate study^a^ |

^a^ Results of inadequate study/studies were ignored.

^b^ Results of indeterminate study/studies were ignored.

BMI = body mass index; ICC = intraclass correlation coefficient; SEM = standard error of measurement; TESS = Toronto Extremity Salvage Score.

**Online Resource 11**

**Results content validity per study: Baecke questionnaire**

|  | **Arakawa Martins et al.**[19] | **Rating of reviewers** | **Rating of reviewers** | **Rating of reviewers** | **Rating of reviewers** | **Overall rating** | **Overall rating** | **Overall rating** | **Overall rating** | **Quality of evidence** | **Quality of evidence** | **Quality of evidence** | **Quality of evidence** |
| --- | --- | --- | --- | --- | --- | --- | --- | --- | --- | --- | --- | --- | --- |
|  | **TI** | **WI** | **SI** | **LI** | **TI** | **WI** | **SI** | **LI** | **TI** | **WI** | **SI** | **LI** | **TI** |
| 1. Are the included items relevant for the construct of interest? |  | - | - | + | - |  |  |  |  |  |  |  |  |
| 2. Are the included items relevant for the target population of interest? |  | ? | ? | ? | ? |  |  |  |  |  |  |  |  |
| 3. Are the included items relevant for the context of use of interest? |  | ? | ? | ? | ? |  |  |  |  |  |  |  |  |
| 4. Are the response options appropriate? |  | + | + | + | + |  |  |  |  |  |  |  |  |
| 5. Is the recall period appropriate? |  | ? | ? | ? | ? |  |  |  |  |  |  |  |  |
| ***RELEVANCE RATING*** |  | *?* | *?* | *?* | *?* | *?* | *?* | *?* | *?* | *No rating of evidence, rating is indeterminate* | *No rating of evidence, rating is indeterminate* | *No rating of evidence, rating is indeterminate* | *No rating of evidence, rating is indeterminate* |
|  |  |  |  |  |  |  |  |  |  |  |  |  |  |
| 6. Are all key concepts included? |  | + | + | - | - |  |  |  |  |  |  |  |  |
| ***COMPREHENSIVENESS RATING*** |  | *+* | *+* | *-* | *-* | *+* | *+* | *-* | *-* | *Very low* | *Very low* | *Very low* | *Very low* |
|  |  |  |  |  |  |  |  |  |  |  |  |  |  |
| 7. Are the PROM instructions understood by the population of interest as intended? | + |  |  |  |  |  |  |  |  |  |  |  |  |
| 8. Are the PROM items and response options understood by the population of interest as intended? | ? |  |  |  |  |  |  |  |  |  |  |  |  |
| 9. Are the PROM items appropriately worded? |  | + | + | + | + |  |  |  |  |  |  |  |  |
| 10. Do the response options match the question? |  | + | + | + | + |  |  |  |  |  |  |  |  |
| ***COMPREHENSIBILITY RATING*** | *?* | *+* | *+* | *+* | *+* | *+* | *+* | *+* | *+* | *Very low* | *Very low* | *Very low* | *Very low* |
|  |  |  |  |  |  |  |  |  |  |  |  |  |  |
| ***CONTENT VALIDITY RATING*** |  | *?* | *?* | *?* | *?* | *?* | *?* | *?* | *?* | *No rating of evidence, rating is indeterminate* | *No rating of evidence, rating is indeterminate* | *No rating of evidence, rating is indeterminate* | *No rating of evidence, rating is indeterminate* |

+ = sufficient; - = insufficient; ? = indeterminate; ± = inconsistent.

LI = Leisure-time Index; SI = Sports Index; TI = Total Index; WI = Work Index.

**Results content validity per study: Functional Mobility Assessment**

|  | **Rating of reviewers** | **Overall rating** | **Quality of evidence** |
| --- | --- | --- | --- |
| 1. Are the included items relevant for the construct of interest? | ? |  |  |
| 2. Are the included items relevant for the target population of interest? | + |  |  |
| 3. Are the included items relevant for the context of use of interest? | + |  |  |
| 4. Are the response options appropriate? | - |  |  |
| 5. Is the recall period appropriate? | ? |  |  |
| ***RELEVANCE RATING*** | *?* | *?* | *Very low* |
|  |  |  |  |
| 6. Are all key concepts included? | ? |  |  |
| ***COMPREHENSIVENESS RATING*** | *?* | *?* | *Very low* |
|  |  |  |  |
| 7. Are the PROM instructions understood by the population of interest as intended? |  |  |  |
| 8. Are the PROM items and response options understood by the population of interest as intended? |  |  |  |
| 9. Are the PROM items appropriately worded? | - |  |  |
| 10. Do the response options match the question? | + |  |  |
| ***COMPREHENSIBILITY RATING*** | *±* | *±* | *Very low* |
|  |  |  |  |
| ***CONTENT VALIDITY RATING*** | *?* | *?* | *No rating of evidence, rating is indeterminate* |

+ = sufficient; - = insufficient; ? = indeterminate; ± = inconsistent.

**Results content validity per study: Karnofsky Performance Status**

|  | **Rating of reviewers** | **Overall rating** | **Quality of evidence** |
| --- | --- | --- | --- |
| 1. Are the included items relevant for the construct of interest? | ? |  |  |
| 2. Are the included items relevant for the target population of interest? | ? |  |  |
| 3. Are the included items relevant for the context of use of interest? | + |  |  |
| 4. Are the response options appropriate? | - |  |  |
| 5. Is the recall period appropriate? | ? |  |  |
| ***RELEVANCE RATING*** | *?* | *?* | *Very low* |
|  |  |  |  |
| 6. Are all key concepts included? | - |  |  |
| ***COMPREHENSIVENESS RATING*** | *-* | *-* | *Very low* |
|  |  |  |  |
| 7. Are the PROM instructions understood by the population of interest as intended? |  |  |  |
| 8. Are the PROM items and response options understood by the population of interest as intended? |  |  |  |
| 9. Are the PROM items appropriately worded? | - |  |  |
| 10. Do the response options match the question? | - |  |  |
| ***COMPREHENSIBILITY RATING*** | *-* | *-* | *Very low* |
|  |  |  |  |
| ***CONTENT VALIDITY RATING*** | *?* | *?* | *No rating of evidence, rating is indeterminate* |

+ = sufficient; - = insufficient; ? = indeterminate; ± = inconsistent.

**Results content validity per study: Mankin score**

|  | **Rating of reviewers** | **Overall rating** | **Quality of evidence** |
| --- | --- | --- | --- |
| 1. Are the included items relevant for the construct of interest? | ? |  |  |
| 2. Are the included items relevant for the target population of interest? | + |  |  |
| 3. Are the included items relevant for the context of use of interest? | ? |  |  |
| 4. Are the response options appropriate? | - |  |  |
| 5. Is the recall period appropriate? | ? |  |  |
| ***RELEVANCE RATING*** | *?* | *-* | *Very low* |
|  |  |  |  |
| 6. Are all key concepts included? | - |  |  |
| ***COMPREHENSIVENESS RATING*** | *-* | *-* | *Very low* |
|  |  |  |  |
| 7. Are the PROM instructions understood by the population of interest as intended? |  |  |  |
| 8. Are the PROM items and response options understood by the population of interest as intended? |  |  |  |
| 9. Are the PROM items appropriately worded? | - |  |  |
| 10. Do the response options match the question? | - |  |  |
| ***COMPREHENSIBILITY RATING*** | *-* | *-* | *Very low* |
|  |  |  |  |
| ***CONTENT VALIDITY RATING*** | *?* | *?* | *No rating of evidence, rating is indeterminate* |

+ = sufficient; - = insufficient; ? = indeterminate; ± = inconsistent.

**Results content validity per study: MusculoSkeletal Tumor Society 1987 score**

|  | **Rating of reviewers** | **Overall rating** | **Quality of evidence** |
| --- | --- | --- | --- |
| 1. Are the included items relevant for the construct of interest? | ? |  |  |
| 2. Are the included items relevant for the target population of interest? | + |  |  |
| 3. Are the included items relevant for the context of use of interest? | + |  |  |
| 4. Are the response options appropriate? | - |  |  |
| 5. Is the recall period appropriate? | ? |  |  |
| ***RELEVANCE RATING*** | *?* | *?* | *Very low* |
|  |  |  |  |
| 6. Are all key concepts included? | ? |  |  |
| ***COMPREHENSIVENESS RATING*** | *?* | *?* | *Very low* |
|  |  |  |  |
| 7. Are the PROM instructions understood by the population of interest as intended? |  |  |  |
| 8. Are the PROM items and response options understood by the population of interest as intended? |  |  |  |
| 9. Are the PROM items appropriately worded? | - |  |  |
| 10. Do the response options match the question? | - |  |  |
| ***COMPREHENSIBILITY RATING*** | *-* | *-* | *Very low* |
|  |  |  |  |
| ***CONTENT VALIDITY RATING*** | *?* | *?* | *No level of evidence, rating is indeterminate* |

+ = sufficient; - = insufficient; ? = indeterminate; ± = inconsistent.

**Results content validity per study: MusculoSkeletal Tumor Society 1993 score – Lower extremity**

|  | **Kask et al.** [20] | **Mallet et al.** [21] | **Ocaktan et al.** [22] | **Rebolledo et al.** [23] | **Rating of reviewers** | **Overall rating** | **Quality of evidence** |
| --- | --- | --- | --- | --- | --- | --- | --- |
| 1. Are the included items relevant for the construct of interest? |  | ? |  |  | ? |  |  |
| 2. Are the included items relevant for the target population of interest? |  | ? |  |  | + |  |  |
| 3. Are the included items relevant for the context of use of interest? |  | ? |  |  | + |  |  |
| 4. Are the response options appropriate? |  | ? |  |  | - |  |  |
| 5. Is the recall period appropriate? |  | ? |  |  | ? |  |  |
| ***RELEVANCE RATING*** |  | *?* |  |  | *?* | *?* | *Very low* |
|  |  |  |  |  |  |  |  |
| 6. Are all key concepts included? |  | ? |  |  | ? |  |  |
| ***COMPREHENSIVENESS RATING*** |  | *?* |  |  | *?* | *?* | *Very low* |
|  |  |  |  |  |  |  |  |
| 7. Are the PROM instructions understood by the population of interest as intended? | + | ? | ? | ? |  |  |  |
| 8. Are the PROM items and response options understood by the population of interest as intended? | + | ? | ? | + |  |  |  |
| 9. Are the PROM items appropriately worded? |  |  |  |  | - |  |  |
| 10. Do the response options match the question? |  |  |  |  | - |  |  |
| ***COMPREHENSIBILITY RATING*** | *+* | *?* | *?* | *+* | *-* | *+* | *Moderate* |
|  |  |  |  |  |  |  |  |
| ***CONTENT VALIDITY RATING*** |  | *?* |  |  | *?* | *?* | *No level of evidence, rating is indeterminate* |

+ = sufficient; - = insufficient; ? = indeterminate; ± = inconsistent.

**Results content validity per study: Reintegration to Normal Living Index**

|  | **Okoye et al.** [24] | **Rating of reviewers** | **Overall rating** | **Quality of evidence** |
| --- | --- | --- | --- | --- |
| 1. Are the included items relevant for the construct of interest? | + | + |  |  |
| 2. Are the included items relevant for the target population of interest? | + | + |  |  |
| 3. Are the included items relevant for the context of use of interest? | + | + |  |  |
| 4. Are the response options appropriate? | + | + |  |  |
| 5. Is the recall period appropriate? | ? | ? |  |  |
| ***RELEVANCE RATING*** | *IGBO: +* | *English: +* | *+* | *Low* |
|  |  |  |  |  |
| 6. Are all key concepts included? | + | + |  |  |
| ***COMPREHENSIVENESS RATING*** | *IGBO: +* | *English: +* | *+* | *Low* |
|  |  |  |  |  |
| 7. Are the PROM instructions understood by the population of interest as intended? | + |  |  |  |
| 8. Are the PROM items and response options understood by the population of interest as intended? | + |  |  |  |
| 9. Are the PROM items appropriately worded? |  | + |  |  |
| 10. Do the response options match the question? |  | + |  |  |
| ***COMPREHENSIBILITY RATING*** | *IGBO: +* | *English: +* | *+* | *Low* |
|  |  |  |  |  |
| ***CONTENT VALIDITY RATING*** | *IGBO: +* | *English: +* | *+* | *Low* |

+ = sufficient; - = insufficient; ? = indeterminate; ± = inconsistent.

**Results content validity per study: Toronto Extremity Salvage Score – Lower extremity**

|  | **Davis et al.** [13] | **Davis et al.** [25] | **Kask et al.** [20] | **Kim et al.** [26] | **Ocaktan et al.** [22] | **Rossi et al.** [27] | **Saebye et al.** [28] | **Saraiva et al.** [29] | **Srisawat et al.** [30] | **Trost et al.** [31] | **Rating of reviewers** | **Overall rating** | **Quality of evidence** |
| --- | --- | --- | --- | --- | --- | --- | --- | --- | --- | --- | --- | --- | --- |
| 1. Are the included items relevant for the construct of interest? | + |  |  |  |  |  |  |  | ? |  | + |  |  |
| 2. Are the included items relevant for the target population of interest? | + | + |  |  |  |  |  |  | ? |  | + |  |  |
| 3. Are the included items relevant for the context of use of interest? | + |  |  |  |  |  |  |  | ? |  | + |  |  |
| 4. Are the response options appropriate? | ? | ? |  |  |  |  |  |  | ? |  | + |  |  |
| 5. Is the recall period appropriate? | ? | ? |  |  |  |  |  |  | ? |  | + |  |  |
| ***RELEVANCE RATING*** | *+* | *±* |  |  |  |  |  |  | *?* |  | *+* | *+* | *Very low* |
|  |  |  |  |  |  |  |  |  |  |  |  |  |  |
| 6. Are all key concepts included? | + | + |  |  |  | + |  |  | + |  | + |  |  |
| ***COMPREHENSIVENESS RATING*** | *+* | *+* |  |  |  | *+* |  |  | *+* |  | *+* | *+* | *Moderate* |
|  |  |  |  |  |  |  |  |  |  |  |  |  |  |
| 7. Are the PROM instructions understood by the population of interest as intended? |  |  | + | ? | ? | ? | ? | ? | ? | ? |  |  |  |
| 8. Are the PROM items and response options understood by the population of interest as intended? |  |  | + | ? | ? | + | ? | + | + | + |  |  |  |
| 9. Are the PROM items appropriately worded? |  |  |  |  |  |  |  |  |  |  | + |  |  |
| 10. Do the response options match the question? |  |  |  |  |  |  |  |  |  |  | + |  |  |
| ***COMPREHENSIBILITY RATING*** |  |  | *+* | *?* | *?* | *+* | *?* | *+* | *+* | *+* | *+* | *+* | *Moderate* |
|  |  |  |  |  |  |  |  |  |  |  |  |  |  |
| ***CONTENT VALIDITY RATING*** |  |  |  |  |  |  |  |  |  |  | *+* | *+* | *Moderate* |

+ = sufficient; - = insufficient; ? = indeterminate; ± = inconsistent.

**Online Resource 12**

**Results other measurement properties per study: Baecke questionnaire**

| **BAECKE** | **Country (language^a^)** | **Structural validity** | | | **Internal consistency** | | |
| --- | --- | --- | --- | --- | --- | --- | --- |
|  |  | *n* | *Meth qual* | *Result (rating)* | *n* | *Meth qual* | *Result (rating)* |
| Albanes et al. [45] | USA (English) |  |  |  |  |  |  |
| Baecke et al. [16] | The Netherlands (Dutch) | 306 | A | Overall explained variation 55%  - Work Index (8 items): factor loadings ≥0.57 (+) - Sports Index (4 items): factor loadings ≥0.59 (+) - Leisure-time Index (4 items): factor loadings ≥0.50 (+) |  |  |  |
| Burns et al. [32] | USA (English) | 213 | A | Total explained item covariation 74%  - Work Index (8 items): factor loadings ≥0.42 (+) - Leisure Index (8 items): 3 items with factor loadings 0.30-0.37, 5 items with factor loadings ≥0.46 (-) | 213 | V | WI (8 items): α = 0.83 (+) LI (8 items): α = 0.69 (-) |
| Carvalho et al. [33] | Brazil (Brazilian-Portuguese) |  |  |  |  |  |  |
| Florindo et al. [44] | Brazil (Brazilian-Portuguese) |  |  |  |  |  |  |
| Florindo et al. [34] | Brazil (Brazilian-Portuguese) |  |  |  | 1. 326  2. 326  3. 326 | 1. V  2. V  3. V | 1. WI: α = 0.52 (-) 2. SI: α = 0.52 (-) 3. LI: α = 0.62 (-) |
| Florindo et al. [35] | Brazil (Brazilian-Portuguese) |  |  |  |  |  |  |
| Jacobs et al. [36] | USA (English) |  |  |  |  |  |  |
| Morelhão et al. [46] | Brazil (Brazilian-Portuguese) |  |  |  |  |  |  |
| Ono et al. [37] | Japan (Japanese) |  |  |  |  |  |  |
| Philippaerts et al. [38] | Belgium (Dutch) |  |  |  |  |  |  |
| Philippaerts et al. [39] | Belgium (Dutch) |  |  |  |  |  |  |
| Philippaerts et al. [43] | Belgium (Dutch) |  |  |  |  |  |  |
| Rocha et al. [40] | Brazil (Brazilian-Portuguese) | 241 | V | Work Index (8 items), Sports Index (4 items), Leisure-time Index (4 items): CFI: 0.935; TLI: 0.923; RMSEA: 0.073; SRMR: 0.077 (+) |  |  |  |
| Sadeghisani et al. [41] | Iran (Persian) |  |  |  | 126 | I | TI: α > 0.7 (+) |
| Tebar et al. [42] | Brazil (Brazilian-Portuguese) |  |  |  |  |  |  |
| **Pooled or summary result**  **(overall rating)** | | **1. 547 2. 547 3. 547** | **1. Moderate 2. Moderate 3. Moderate** | **1. WI (8 items) (+) 2. SI (4 items) (+) 3. LI (4 items) (+)** | **1. 326 2. 326 3. 326** | **1. Moderate 2. Moderate 3. Moderate** | **1. WI: α = 0.52 (-) 2. SI: α = 0.52 (-) 3. LI: α = 0.62 (-)** |

Text in red = outcomes ignored in summary result.

V = very good methodological quality; A = adequate methodological quality; D = doubtful methodological quality; I = inadequate methodological quality.

+ = sufficient; - = insufficient; ? = indeterminate; ± = inconsistent.

CFI = comparative fit index; LI = Leisure-time Index; RMSEA = root mean square error of approximation; SI = Sports Index; SRMR = standardized root mean square residual; TI = Total Index; TLI = Tucker-Lewis index; WI = Work Index.

^a^Language in which the instrument was evaluated.

| **BAECKE** | **Country (language^a^)** | **Cross-cultural validity/ measurement invariance** | | | **Reliability**  **a = test-retest; b = inter-rater** | | | **Measurement error** | | |
| --- | --- | --- | --- | --- | --- | --- | --- | --- | --- | --- |
|  |  | *n* | *Meth qual* | *Result (rating)* | *n* | *Meth qual* | *Result (rating)* | *n* | *Meth qual* | *Result (rating)* |
| Albanes et al. [45] | USA (English) |  |  |  |  |  |  |  |  |  |
| Baecke et al. [16] | The Netherlands (Dutch) |  |  |  | a1. 277 a2. 277 a3. 277 | a1. I a2. I a3. I | a1. WI: r = 0.88 (+) a2. SI: r = 0.81 (+) a3. LI: r = 0.74 (+) |  |  |  |
| Burns et al. [32] | USA (English) |  |  |  |  |  |  |  |  |  |
| Carvalho et al. [33] | Brazil (Brazilian-Portuguese) |  |  |  | a1. 73 a2. 73 a3. 73 a4. 73 | a1. D a2. D a3. D a4. D | a1. WI: ICC = 0.84 (+) a2. SI: ICC = 0.83 (+) a3. LI: ICC = 0.61 (-) a4. TI: ICC = 0.77 (+) |  |  |  |
| Florindo et al. [44] | Brazil (Brazilian-Portuguese) |  |  |  | a1. 21 a2. 21 | a1. D a2. D | a1. SI: ICC = 0.69 (-) a2. LI: ICC = 0.80 (+) |  |  |  |
| Florindo et al. [34] | Brazil (Brazilian-Portuguese) |  |  |  |  |  |  |  |  |  |
| Florindo et al. [35] | Brazil (Brazilian-Portuguese) |  |  |  | a1. 29 a2. 29 a3. 29 a4. 29 | a1. D a2. D a3. D a4. D | a1. WI: ICC = 0.85 (+) a2. SI: ICC = 0.70 (+) a3. LI: ICC = 0.44 (-) a4. TI: ICC = 0.72 (+) |  |  |  |
| Jacobs et al. [36] | USA (English) |  |  |  | a1. 76 a2. 76 a3. 76 a4. 76 | a1. D a2. D a3. D a4. D | a1. WI: Test-retest correlation coefficient = 0.78 (?) a2. SI: Test-retest correlation coefficient = 0.90 (?) a3. LI: Test-retest correlation coefficient = 0.86 (?) a4. TI: Test-retest correlation coefficient = 0.93 (?) |  |  |  |
| Morelhão et al. [46] | Brazil (Brazilian-Portuguese) |  |  |  |  |  |  |  |  |  |
| Ono et al. [37] | Japan (Japanese) |  |  |  | a1. 52 a2. 52 a3. 52 a4. 52 | a1. I a2. I a3. I a4. I | a1. WI: ICC = 0.84 (+) a2. SI: ICC = 0.83 (+) a3. LI: ICC = 0.78 (+) a4. TI: ICC = 0.87 (+) |  |  |  |
| Philippaerts et al. [38] | Belgium (Dutch) |  |  |  | a1. 90 a2. 90 a3. 90 a4. 90 | a1. A a2. A a3. A a4. A | a1. WI: ICC = 0.95 (+) a2. SI: ICC = 0.93 (+) a3. LI: ICC = 0.87 (+) a4. TI: ICC = 0.86 (+) |  |  |  |
| Philippaerts et al. [39] | Belgium (Dutch) |  |  |  |  |  |  |  |  |  |
| Philippaerts et al. [43] | Belgium (Dutch) |  |  |  |  |  |  |  |  |  |
| Rocha et al. [40] | Brazil (Brazilian-Portuguese) |  |  |  |  |  |  |  |  |  |
| Sadeghisani et al. [41] | Iran (Persian) |  |  |  | a1. 32 a2. 32 a3. 32 a4. 32 | a1. D a2. D a3. D a4. D | a1. WI: ICC = 0.95 (+) a2. SI: ICC = 0.93 (+) a3. LI: ICC = 0.77 (+) a4. TI: ICC = 0.88 (+) |  |  |  |
| Tebar et al. [42] | Brazil (Brazilian-Portuguese) |  |  |  | a1. 50 a2. ? a3. ? a4. ? | a1. D a2. I a3. I a4. I | a1. TI whole sample: ICC = 0.72 (+) a2. TI low educational level sample: ICC = 0.67 (-) a3. TI medium educational level sample: ICC = 0.75 (+) a4. TI high educational level sample: ICC = 0.97 (+) |  |  |  |
| **Pooled or summary result**  **(overall rating)** | |  |  |  | **a1. 224**  **a2. 245**  **a3. 143**  **73**  **52**  **29**  **a4. 274** | **a1. Low**  **a2. Low**  **a3. Low**  **Very low**  **Very low**  **Very low**  **a4. Low** | **a1. WI: ICC = 0.84-0.95 (+)**  **a2. SI: ICC = 0.69-0.93 (+)**  **a3. LI: General population: ICC = 0.77-0.87 (+)**  **Chronic low back pain: ICC = 0.61 (-)**  **Women with hip disorders: ICC = 0.78 (+)**  **Adults with HIV/AIDS: ICC = 0.44 (-)**  **a4. TI: ICC = 0.72-0.88 (+)** |  |  |  |

Text in red = outcomes ignored in summary result.

V = very good methodological quality; A = adequate methodological quality; D = doubtful methodological quality; I = inadequate methodological quality.

+ = sufficient; - = insufficient; ? = indeterminate; ± = inconsistent.

ICC = intraclass correlation coefficient; LI = Leisure-time Index; SI = Sports Index; TI = Total Index; WI = Work Index.

^a^Language in which the instrument was evaluated.

| **BAECKE** | **Country (language^a^)** | **Criterion validity** | | | **Hypotheses testing**  **a = comparison with other instruments**  **b = comparison between subgroups** | | | **Responsiveness**  **a = comparison to gold standard**  **b = comparison with other instruments**  **c = comparison between subgroups**  **d = before and after intervention** | | |
| --- | --- | --- | --- | --- | --- | --- | --- | --- | --- | --- |
|  |  | *n* | *Meth qual* | *Result (rating)* | *n* | *Meth qual* | *Result (rating)* | *n* | *Meth qual* | *Result (rating)* |
| Albanes et al. [45] | USA (English) |  |  |  | a1. 21 a2. 21 a3. 21 a4. 21 a5. 21 a6. 21 a7. 21 a8. 21 a9. 21 | a1. V a2. V a3. I a4. I a5. I a6. I a7. A a8. I a9. I | a1. TI vs intake: ρ = 0.38 (+) a2. TI vs REE: ρ = 0.21 (+) a3. TI vs Harvard Alumni: ρ = 0.56 (+) a4. TI vs Pennsylvania Alumni: ρ = 0.59 (+) a5. TI vs Five-City Project: ρ = 0.16 (-) a6. TI vs Framingham: ρ = 0.57 (+) a7. TI vs Minnesota Leisure: ρ = 0.36 (+) a8. TI vs Health Insurance Plan: ρ = 0.78 (+) a9. TI vs Lipid Research Clinics: ρ = 0.68 (+) |  |  |  |
| Baecke et al. [16] | The Netherlands (Dutch) |  |  |  |  |  |  |  |  |  |
| Burns et al. [32] | USA (English) |  |  |  |  |  |  |  |  |  |
| Carvalho et al. [33] | Brazil (Brazilian-Portuguese) |  |  |  | a1. 73    a2. 73 a3. 73 | a1. A    a2. A a3. V | a1. TI vs counts: ρ = 0.18 (-) TI vs VM counts: ρ = 0.26 (-) TI vs MVPA: ρ = 0.17 (-) TI vs steps: ρ = 0.27 (-) a2. TI vs pain: ρ = -0.16 (+) a3. TI vs QBPDS: ρ = -0.01 (-) |  |  |  |
| Florindo et al. [44] | Brazil (Brazilian-Portuguese) |  |  |  | a1. 21  a2. 21  a3. 21 a4. 21 a5. 21 a6. 21 | a1. I  a2. I  a3. I a4. I a5. I a6. I | a1. SI vs VO2 max: r = 0.04 (-) SI vs %DHR: r = 0.38 (+) a2. LI vs VO2 max: r = 0.24 (+) LI vs %DHR: r = 0.47 (+) a3. SI vs IPE: r = 0.56 (+) a4. LI vs IPE: r = 0.28 (+) a5. SI vs ILA: r = 0.25 (+) a6. LI vs ILA: r = 0.64 (+) |  |  |  |
| Florindo et al. [34] | Brazil (Brazilian-Portuguese) |  |  |  |  |  |  |  |  |  |
| Florindo et al. [35] | Brazil (Brazilian-Portuguese) |  |  |  | a1. 24  a2. 24  a3. 24  a4. 24  a5. 21 a6. 21 a7. 21 a8. 21 | a1. A  a2. A  a3. A  a4. A  a5. A a6. A a7. A a8. A | a1. WI vs peak VO2: r = -0.14 (+) WI vs peak workload: r = 0.02 (+) a2. SI vs peak VO2: r = 0.41 (+) SI vs peak workload: r = 0.43 (+) a3. LI vs peak VO2: r = 0.19 (+) LI vs peak workload: r = 0.13 (-) a4. TI vs peak VO2: r = 0.27 (-) TI vs peak workload: r = 0.30 (+) a5. WI vs energy expenditure: r = 0.64 (-) a6. SI vs energy expenditure: r = -0.11 (-) a7. LI vs energy expenditure: r = -0.06 (-) a8. TI vs energy expenditure: r = 0.24 (-) |  |  |  |
| Jacobs et al. [36] | USA (English) |  |  |  | a1. 73  a2. 73  a3. 73  a4. 73  a5. 64  a6. 64  a7. 64  a8. 64  a9. 71 a10. 71 a11. 71 a12. 71 a13. 64 a14. 64 a15. 64 a16. 64  a17. 73  a18. 73  a19. 73  a20. 73 | a1. D  a2. D  a3. D  a4. D  a5. V  a6. V  a7. V  a8. V  a9. V a10. V a11. V a12. V a13. V  a14. V a15. V a16. V  a17. D  a18. D  a19. D  a20. D | a1. WI vs Caltrac: Caloric expenditure:  ρ = -0.03 (-) WI vs Caltrac: Motion: ρ = 0.11 (-) a2. SI vs Caltrac: Caloric expenditure: ρ = -0.02 (-) SI vs Caltrac: Motion: ρ = 0.32 (+) a3. LI vs Caltrac: Caloric expenditure: ρ = -0.11 (-) LI vs Caltrac: Motion: ρ = 0.01 (-) a4. TI vs Caltrac: Caloric expenditure: ρ = -0.08 (-) TI vs Caltrac: Motion: ρ = 0.19 (-) a5. WI vs Workload 160 (treadmill): ρ = 0.06 (+) WI vs VO2 max (treadmill): ρ = 0.23 (+) a6. SI vs Workload 160 (treadmill): ρ = 0.57 (+) SI vs VO2 max (treadmill): ρ = 0.52 (+) a7. LI vs Workload 160 (treadmill): ρ = 0.33 (+) LI vs VO2 max (treadmill): ρ = 0.26 (+) a8. TI vs Workload 160 (treadmill): ρ = 0.51 (+) TI vs VO2 max (treadmill): ρ = 0.54 (+) a9. WI vs Hydrostatic weighing: ρ = -0.17 (+) a10. SI vs Hydrostatic weighing: ρ = -0.35 (+) a11. LI vs Hydrostatic weighing: ρ = -0.39 (+) a12. TI vs Hydrostatic weighing: ρ = -0.49 (+) a13. WI vs FEV: ρ = -0.16 (+) a14. SI vs FEV: ρ = -0.06 (-) a15. LI vs FEV: ρ = 0.02 (-) a16. TI vs FEV: ρ = -0.01 (-)  a17. WI vs 4-week history- total: ρ = 0.05 (+) WI vs 4-week history -light: ρ = -0.07 (+) WI vs 4-week history -moderate: ρ = -0.07 (+) WI vs 4-week history -heavy: ρ = 0.07 (+) WI vs 4-week history - household: ρ = 0.01 (+) a18. SI vs 4-week history -total: ρ = 0.40 (+) SI vs 4-week history -light: ρ = 0.30 (+) SI vs 4-week history -moderate: ρ = -0.03 (-) SI vs 4-week history -heavy: ρ = 0.71 (+) SI vs 4-week history -household: ρ = -0.02 (+) a19. LI vs 4-week history -total: ρ = 0.28 (-) LI vs 4-week history -light: ρ = 0.69 (+) LI vs 4-week history -moderate: ρ = -0.02 (-) LI vs 4-week history -heavy: ρ = 0.23 (-) LI vs 4-week history -household: ρ = 0.03 (+) a20. TI vs 4-week history-total: ρ = 0.37 (+) TI vs 4-week history -light: ρ = 0.50 (+) TI vs 4-week history -moderate: ρ = 0.02 (-) TI vs 4-week history -heavy: ρ = 0.54 (+) TI vs 4-week history -household: ρ = -0.03 (+) |  |  |  |
| Morelhão et al. [46] | Brazil (Brazilian-Portuguese) |  |  |  | a1. 106 a2. 106 | a1. V a2. V | a1. TI vs RMDQ: ρ = 0.09 (-) a2. TI vs QBPDS: ρ = -0.10 (-) | d. 106 | d. V | d. TI: Baseline vs 2 months FU: ES = 0.12, SRM = 0.08 (-) |
| Ono et al. [37] | Japan (Japanese) |  |  |  | a1. 61 a2. 61 a3. 61 a4. 61 | a1. V a2. V a3. V a4. V | a1. WI vs steps: ρ = 0.42 (+) a2. SI vs steps: ρ = 0.30 (+) a3. LI vs steps: ρ = 0.42 (+) a4. TI vs steps: ρ = 0.49 (+) |  |  |  |
| Philippaerts et al. [38] | Belgium (Dutch) |  |  |  | b1. 60   b2. 60  b3. 60   b4. 60 | b1. D  b2. D   b3. D   b4. D | b1. WI: Managers vs clerks: mean = 2.5 vs 2.3 (+) WI: Managers vs workmen: mean = 2.5 vs 3.3 (+) WI: Clerks vs workmen: mean = 2.3 vs 3.3 (+) b2. SI: Managers vs clerks: mean = 2.8 vs 3.0 (+) SI: Managers vs workmen: mean = 2.8 vs 2.8 (+) SI: Clerks vs workmen: mean = 3.0 vs 2.8 (+) b3. LI: Managers vs clerks: mean = 2.8 vs 2.8 (+) LI: Managers vs workmen: mean = 2.8 vs 2.6 (+) LI: Clerks vs workmen: mean = 2.8 vs 2.6 (+) b4. TI: Managers vs clerks: mean = 7.9 vs 8.0 (+) TI: Managers vs workmen: mean = 7.9 vs 8.8 (+) TI: Clerks vs workmen: mean = 8.0 vs 8.8 (+) |  |  |  |
| Philippaerts et al. [39] | Belgium (Dutch) |  |  |  | a1. 19 a2. 19 a3. 19 a4. 19 a5. 19 a6. 19 a7. 19 a8. 19 | a1. A a2. A a3. A a4. A a5. A a6. A a7. A a8. A | a1. WI vs ADMR: r = 0.37 (+) a2. WI vs PAL: r = 0.52 (+) a3. SI vs ADMR: r = 0.46 (+) a4. SI vs PAL: r = 0.55 (+) a5. LI vs ADMR: r = 0.50 (+) a6. LI vs PAL: r = 0.22 (-) a7. TI vs ADMR: r = 0.68 (+) a8. TI vs PAL: r = 0.69 (+) |  |  |  |
| Philippaerts et al. [43] | Belgium (Dutch) |  |  |  | a1. 133  a2. 134  a3. 134  a4. 134 | a1. A  a2. A a3. A  a4. V | a1. WI vs Tracmor work time: r = 0.42 (-) a2. SI vs Tracmor active leisure time: r = 0.34 (-) a3. LI vs Tracmor quiet leisure time: r = 0.28 (-)  a4. TI vs Tracmor total time: r = 0.47 (-) |  |  |  |
| Rocha et al. [40] | Brazil (Brazilian-Portuguese) |  |  |  |  |  |  |  |  |  |
| Sadeghisani et al. [41] | Iran (Persian) |  |  |  | a1. 126 a2. 126 a3. 126 a4. 126 | a1. A a2. A a3. A a4. A | a1. WI vs IPAQ: r = 0.03 (-) a2. SI vs IPAQ: r = 0.01 (-) a3. LI vs IPAQ: r = 0.04 (-) a4. TI vs IPAQ: r = 0.19 (-) |  |  |  |
| Tebar et al. [42] | Brazil (Brazilian-Portuguese) |  |  |  | a1. 251 a2. 251 a3. 251 a4. 251 b1. 251  b2. 251  b3. 251  b4. 251 | a1. A a2. A a3. A a4. V b1. I  b2. I  b3. I  b4. I | a1. WI vs MVPA: ρ = 0.17 (+) a2. SI vs MVPA: ρ = 0.26 (-) a3. LI vs MVPA: ρ = 0.25 (-) a4. TI vs MVPA: ρ = 0.33 (+) b1. WI: High vs medium vs low educational level:  ρ = -0.02 (?) b2. SI: High vs medium vs low educational level:  ρ = 0.31 (?) b3. LI: High vs medium vs low educational level:  ρ = 0.08 (?) b4. TI: High vs medium vs low educational level:  ρ = 0.18 (?) |  |  |  |
| **Pooled or summary result**  **(overall rating)** | |  |  |  | **a. 999**  **b. 60**  **a. 1061**  **b. 60**  **a. 1061**  **b. 60**  **a. 1494**  **b. 60** | **a. Moderate**  **b. Very low**  **a. Moderate**  **b. Very low**  **a. Moderate**  **b. Very low**  **a. Moderate**  **b. Very low** | **a. WI: 15+ 5- (+)**  **b. WI: 3+ (+)**  **a. SI: 13+ 7- (±)**  **b. SI: 3+ (+)**  **a. LI: 8+ 12- (±)**  **b. LI: 3+ (+)**  **a. TI: 16+ 15- (±)**  **b. TI: 3+ (+)** | **d. 106** | **d. Moderate** | **d. TI: 1- (-)** |

Text in red = outcomes ignored in summary result.

V = very good methodological quality; A = adequate methodological quality; D = doubtful methodological quality; I = inadequate methodological quality.

+ = sufficient; - = insufficient; ? = indeterminate; ± = inconsistent.

ADMR = average daily metabolic rate; DHR = decrease of the hearts rate; FEV = forced expiratory volume; FU = follow-up; ILA = index of locomotion activities; IPAQ = International Physical Activity Questionnaire; IPE = index of physical exercise; LI = Leisure-time Index; MVPA = moderate to vigorous physical activity; PAL = physical activity level; QBPDS = Quebec Back Pain Disability Scale; REE = resting energy expenditure; RMDQ = Roland Morris Disability Questionnaire; SI = Sports Index; SRM = standardized response mean; TI = Total Index; VM = vector magnitude; WI = Work Index.

^a^Language in which the instrument was evaluated.

**Results other measurement properties per study: Functional Mobility Assessment**

|  | **FMA** | **Country (language^a^)** | **Structural validity** | | | **Internal consistency** | | |
| --- | --- | --- | --- | --- | --- | --- | --- | --- |
|  |  |  | *n* | *Meth qual* | *Result (rating)* | *n* | *Meth qual* | *Result (rating)* |
| Marchese et al. [14] | | USA (English) |  |  |  | 23 | D | α = 0.75 (?) |
|  | Pierce et al. [47] | USA (English) |  |  |  |  |  |  |
|  | **Pooled or summary result**  **(overall rating)** | |  |  |  | **23** | **^b^** | **α = 0.75 (?)** |

Text in red = outcomes ignored in summary result.

V = very good methodological quality; A = adequate methodological quality; D = doubtful methodological quality; I = inadequate methodological quality.

+ = sufficient; - = insufficient; ? = indeterminate; ± = inconsistent.

^a^Language in which the instrument was evaluated.

^b^No level of evidence, rating is indeterminate

|  | **FMA** | **Country (language^a^)** | **Cross-cultural validity/ measurement invariance** | | | **Reliability**  **a = test-retest; b = inter-rater** | | | **Measurement error** | | |
| --- | --- | --- | --- | --- | --- | --- | --- | --- | --- | --- | --- |
|  |  |  | *n* | *Meth qual* | *Result (rating)* | *n* | *Meth qual* | *Result (rating)* | *n* | *Meth qual* | *Result (rating)* |
| Marchese et al. [14] | | USA (English) |  |  |  | b. 23 | b. I | b. ICC ≥ 0.97 (+) |  |  |  |
|  | Pierce et al. [47] | USA (English) |  |  |  |  |  |  |  |  |  |
|  | **Pooled or summary result**  **(overall rating)** | |  |  |  | **b. 23** | **b. Very Low** | **b. ICC ≥ 0.97 (+)** |  |  |  |

Text in red = outcomes ignored in summary result.

V = very good methodological quality; A = adequate methodological quality; D = doubtful methodological quality; I = inadequate methodological quality.

+ = sufficient; - = insufficient; ? = indeterminate; ± = inconsistent.

ICC = intraclass correlation coefficient.

^a^Language in which the instrument was evaluated.

|  | **FMA** | **Country (language^a^)** | **Criterion validity** | | | **Hypotheses testing**  **a = comparison with other instruments**  **b = comparison between subgroups** | | | **Responsiveness**  **a = comparison to gold standard**  **b = comparison with other instruments**  **c = comparison between subgroups**  **d = before and after intervention** | | |
| --- | --- | --- | --- | --- | --- | --- | --- | --- | --- | --- | --- |
|  |  |  | *n* | *Meth qual* | *Result (rating)* | *n* | *Meth qual* | *Result (rating)* | *n* | *Meth qual* | *Result (rating)* |
| Marchese et al. [14] | | USA (English) |  |  |  | a1. 94  a2. 94  a3. 94  b. 114 | a1. A  a2. I  a3. A  b. A | a1. FMA vs SF-36 total: r = 0.60 (+)  FMA vs SF-36 PCS: r = 0.64 (+)  FMA vs SF-36 MCS: r = 0.40 (-)  a2. FMA vs MSTS93-LE: r = 0.68 (+)  a3. FMA vs TESS-LE: r = 0.62 (+)  b. Lower extremity sarcoma vs healthy subjects: mean = 48.6 vs 55.9 (+) | d. 6 | d. D | d. 6 vs 12 vs 18 months postop: mean = 38.5 vs 44.7 vs 43.5 (?) |
|  | Pierce et al. [47] | USA (English) |  |  |  | b. 37 | b. V | b. Amputation vs healthy subjects:  mean = 50.45 vs 57.83 (+) |  |  |  |
|  | **Pooled or summary result**  **(overall rating)** | |  |  |  | **a. 188**  **b. 151** | **a. Moderate**  **b. High** | **a. 3+ 1- (+)**  **b. 2+ (+)** | **d. 6** | **d. Very low** | **d. ?** |

Text in red = outcomes ignored in summary result.

V = very good methodological quality; A = adequate methodological quality; D = doubtful methodological quality; I = inadequate methodological quality.

+ = sufficient; - = insufficient; ? = indeterminate; ± = inconsistent.

FMA = Functional Mobility Assessment; MCS = mental component summary; MSTS = MusculoSkeletal Tumor Society 1993 score; PCS = physical component summary; postop = postoperative; SD = standard deviation; SF-36 = 36-Item Short Form Survey; TESS-LE = Toronto Extremity Salvage Score -Lower extremity.

^a^Language in which the instrument was evaluated.

**Results other measurement properties per study: Karnofsky Performance Status**

| **KPS** | **Country (language^a^)** | **Structural validity** | | | **Internal consistency** | | |
| --- | --- | --- | --- | --- | --- | --- | --- |
|  |  | *n* | *Meth qual* | *Result (rating)* | *n* | *Meth qual* | *Result (rating)* |
| Borja et al. [48] | Canada (English) |  |  |  |  |  |  |
| Celtek et al. [49] | Turkey (Turkish) |  |  |  |  |  |  |
| Conill et al. [50] | Spain (Unknown) |  |  |  |  |  |  |
| Crooks et al. [51] | USA (English) |  |  |  |  |  |  |
| Frappaz et al. [52] | France (Unknown) |  |  |  |  |  |  |
| Grieco et al. [53] | USA (English) |  |  |  |  |  |  |
| Hutchinson et al. [54] | Canada (English) |  |  |  |  |  |  |
| Liem et al. [55] | USA (English) |  |  |  |  |  |  |
| Mor et al. [57] | USA (English) |  |  |  |  |  |  |
| Myers et al. [58] | Canada (English) |  |  |  |  |  |  |
| O'Dell et al. [60] | USA (English) |  |  |  |  |  |  |
| O'Dell et al. [59] | USA (English) |  |  |  |  |  |  |
| Roila et al. [61] | Italy (Unknown) |  |  |  |  |  |  |
| Schag et al. [62] | USA (English) |  |  |  |  |  |  |
| Stedman et al. [56] | USA (English) |  |  |  |  |  |  |
| Taylor et al. [63] | Australia (English) |  |  |  |  |  |  |
| Yates et al. [64] | USA (English) |  |  |  |  |  |  |
| Wood et al. [65] | USA (English) |  |  |  |  |  |  |
| **Pooled or summary result**  **(overall rating)** | |  |  |  |  |  |  |

Text in red = outcomes ignored in summary result.

V = very good methodological quality; A = adequate methodological quality; D = doubtful methodological quality; I = inadequate methodological quality.

+ = sufficient; - = insufficient; ? = indeterminate; ± = inconsistent.

^a^Language in which the instrument was evaluated.

|  | **KPS** | **Country (language^a^)** | **Cross-cultural validity/ measurement invariance** | | | **Reliability**  **a = test-retest; b = inter-rater** | | | **Measurement error** | | |
| --- | --- | --- | --- | --- | --- | --- | --- | --- | --- | --- | --- |
|  |  |  | *n* | *Meth qual* | *Result (rating)* | *n* | *Meth qual* | *Result (rating)* | *n* | *Meth qual* | *Result (rating)* |
|  | Borja et al. [48] | Canada (English) |  |  |  | b. 27 | b. I | b. Medical doctor vs radiation therapy student: ρ = 0.81 (?)  Medical doctor vs primary care nurse: ρ = 0.74 (?)  Medical doctor vs radiation therapist: ρ = 0.67 (?) |  |  |  |
|  | Celtek et al. [49] | Turkey (Turkish) |  |  |  |  |  |  |  |  |  |
|  | Conill et al. [50] | Spain (Unknown) |  |  |  | b. 100 | b. I | b. τ = 0.76 (+) |  |  |  |
|  | Crooks et al. [51] | USA (English) |  |  |  |  |  |  |  |  |  |
|  | Frappaz et al. [52] | France (Unknown) |  |  |  | b. 6^b^ | b. I | b. Description of several patient and clinician factors that influence ratings (?) |  |  |  |
|  | Grieco et al. [53] | USA (English) |  |  |  | b. 30 | b. I | b. ρ = 0.86 (?) |  |  |  |
|  | Hutchinson et al. [54] | Canada (English) |  |  |  | b1. 29 b2. 31 | b1. D b2. D | b1. κ = 50% (?) b2. κ = 46% (?) |  |  |  |
|  | Liem et al. [55] | USA (English) |  |  |  | b. 114 | b. I | b. κ = 0.29 (?) |  |  |  |
|  | Mor et al. [57] | USA (English) |  |  |  | b. 17^c^ | b. I | b. Interrater reliability coefficients > 0.97 (?) |  |  |  |
|  | Myers et al. [58] | Canada (English) |  |  |  | b. 134 | b. A | b. Research assistant vs medical doctor: κ = 0.71 (+) Research assistant vs research nurse: κ = 0.62 (-) Research nurse vs medical doctor: κ = 0.58 (-) |  |  |  |
|  | O'Dell et al. [60] | USA (English) |  |  |  | b. 77 | b. I | b. Primary physician vs other physician: r = 0.77 (?) Physician's assistant vs other physician: r = 0.82 (?) |  |  |  |
|  | O'Dell et al. [59] | USA (English) |  |  |  |  |  |  |  |  |  |
|  | Roila et al. [61] | Italy (Unknown) |  |  |  | a. 209 b. 209 | a. D b. A | a. 3 raters: κ = 0.959, 0.960 and 0.993 (+) b. Oncologist vs oncologist: κ = 0.921 (+) |  |  |  |
|  | Schag et al. [62] | USA (English) |  |  |  | b. 75 | b. D | b. κ = 0.53 (?) |  |  |  |
| Stedman et al. [56] | | USA (English) |  |  |  | b. 8197 | b. I | b. ICC = 0.30 (?) |  |  |  |
|  | Taylor et al. [63] | Australia (English) |  |  |  | b. 100 | b. I | b. Level of complete agreement: 63-76% (?) |  |  |  |
|  | Yates et al. [64] | USA (English) |  |  |  | a. 50 b. 52 | a. I b. I | a. r = 0.66 (?) b. r = 0.69 (?) |  |  |  |
|  | Wood et al. [65] | USA (English) |  |  |  |  |  |  |  |  |  |
|  | **Pooled or summary result**  **(overall rating)** | |  |  |  | **a. 209 b1. 209 b2. 134** | **a. Low b1. Moderate b2. Moderate** | **a. κ = 0.959-0.993 (+) b1. Oncologist vs oncologist: κ = 0.921 (+) b2. Raters with distinct medical backgrounds: κ = 0.58-0.71 (±)** |  |  |  |

Text in red = outcomes ignored in summary result.

V = very good methodological quality; A = adequate methodological quality; D = doubtful methodological quality; I = inadequate methodological quality.

+ = sufficient; - = insufficient; ? = indeterminate; ± = inconsistent.

ICC = intraclass correlation coefficient.

^a^ Language in which the instrument was evaluated.

^b^ Clinical case vignettes

^c^ Narrative patient examples

|  | **KPS** | **Country (language^a^)** | **Criterion validity** | | | **Hypotheses testing**  **a = comparison with other instruments**  **b = comparison between subgroups** | | | **Responsiveness**  **a = comparison to gold standard**  **b = comparison with other instruments**  **c = comparison between subgroups**  **d = before and after intervention** | | |
| --- | --- | --- | --- | --- | --- | --- | --- | --- | --- | --- | --- |
|  |  |  | *n* | *Meth qual* | *Result (rating)* | *n* | *Meth qual* | *Result (rating)* | *n* | *Meth qual* | *Result (rating)* |
|  | Borja et al. [48] | Canada (English) |  |  |  |  |  |  |  |  |  |
|  | Celtek et al. [49] | Turkey (Turkish) |  |  |  | a1. 80 a2. 80 b. 80 | a1. A a2. A b. V | a1. KPS vs Katz ADL total score: r = 0.895 (+) a2. KPS vs IADL scale total score: r = –0.894 (-) b. Male vs female: mean = 66.25 vs 62.19 (+) |  |  |  |
|  | Conill et al. [50] | Spain (Unknown) |  |  |  |  |  |  |  |  |  |
|  | Crooks et al. [51] | USA (English) |  |  |  | a1. 134 a2. 134 a3. 134 a4. 134 a5. 134 a6. 134 b. 134 | a1. V a2. D a3. D a4. A a5. A a6. D b. D | a1. KPS vs Katz ADL: r = 0.73 (+) a2. KPS vs IADL: r = 0.66 (+) a3. KPS vs MMSE: r = 0.48 (+) a4. KPS vs BDI: r = -0.42 (+) a5. KPS vs GDS: r = -0.28 (-) a6. KPS vs OSRRS: r = -0.06 (+) b. KPS vs age: r = -0.23 (+) KPS vs days in clinic: r = 0.11 (?) KPS vs no. visits: r = 0.07 (?) KPS vs hospitalized: r = -0.17 (?) KPS vs survival days: r = 0.30 (?) KPS vs community residing: r = 0.38 (?) KPS vs ever institutionalized: r = -0.32 (?) |  |  |  |
|  | Frappaz et al. [52] | France (Unknown) |  |  |  |  |  |  |  |  |  |
|  | Grieco et al. [53] | USA (English) |  |  |  | a1. 100  a2. 90  a3. 100  b. 100 | a1. I a2. D  a3. I  b. D | a1. KPS vs QWB: correlation coefficient = 0.84 (-) a2. KPS vs HPQ: correlation coefficient = 0.68 (-)  a3. KPS vs symptom-problem complexes: correlation coefficient = 0.54 (?)  b. Normal vs outpatient: mean = 99 vs 75 (+) Outpatient vs pain: mean = 75 vs 62 (+) Pain vs dialysis: mean = 62 vs 51 (+) Dialysis vs stroke: mean = 51 vs 39.5 (+) |  |  |  |
|  | Hutchinson et al. [54] | Canada (English) |  |  |  |  |  |  |  |  |  |
|  | Liem et al. [55] | USA (English) |  |  |  | b. 117 | b. I | b. Agreement stage I tumors vs stage IV tumors: 40% vs 41% (?) Agreement definitive cases vs palliative cases: 44% vs 41% (?) Agreement head and neck cancers vs lung cancer: 58% vs 41% (?) |  |  |  |
|  | Mor et al. [57] | USA (English) |  |  |  |  |  |  |  |  |  |
|  | Myers et al. [58] | Canada (English) |  |  |  | a1. 134 a2. 134 a3. 134 | a1. V a2. V a3. D | a1. KPS vs ECOG: ρ = -0.9139 (+) a2. KPS vs PPS: ρ = 0.9628 (+) a3. KPS vs AKPS: ρ = 0.9836 (+) |  |  |  |
|  | O'Dell et al. [60] | USA (English) |  |  |  |  |  |  |  |  |  |
|  | O'Dell et al. [59] | USA (English) |  |  |  | a1. 160 a2. 160 a3. 160 a4. 160 a5. 160   a6. 160 b1. 160 b2. 160 | a1. D a2. D a3. I a4. I a5. D  a6. I b1. I b2. I | a1. KPS vs Global health-VAS: r = 0.5266 (-) a2. KPS vs Global health-Likert: r = -0.5085 (-) a3. KPS vs Total disability score: r = -0.4364 (-) a4. KPS vs Number of symptoms: r = -0.3952 (-) a5. KPS vs MOS-HIV energy/fatigue: r = 0.5182 (+) KPS vs MOS-HIV mental health: r = 0.2423 (+) KPS vs MOS-HIV cognitive: r = 0.2113 (+) a6. ATHOS social function score: r = 0.4588 (-) b1. KPS vs CDC clinical category: r = -0.4832 (+) b2. KPS vs days unable to work: r = -0.5166 (+) KPS vs weekly hours worked: r = 0.4825 (+) |  |  |  |
|  | Roila et al. [61] | Italy (Unknown) |  |  |  |  |  |  |  |  |  |
|  | Schag et al. [62] | USA (English) |  |  |  | a. 75 | a. I | a. KPS vs CIPS self-care: r = -0.16-0.41 (-) KPS vs CIPS daily activity: r = -0.30-0.51 (-) KPS vs CIPS work: r = -0.34-0.39 (+) KPS vs CIPS evidence of disease:  r = 0.24 / -0.42-0.46 (+) |  |  |  |
| Stedman et al. [56] | | USA (English) |  |  |  |  |  |  |  |  |  |
|  | Taylor et al. [63] | Australia (English) |  |  |  |  |  |  |  |  |  |
|  | Yates et al. [64] | USA (English) |  |  |  | a. 52 | a. I | a. KPS vs desire for food: r = 0.40 (?) KPS vs sleep: r = 0.24 (?) KPS vs difficulty with balance: r = 0.61 (?) KPS vs difficulty with stairs: r = 0.63 (?) KPS vs pain level: r = -0.37 (?) KPS vs happiness: r = 0.12 (?) KPS vs positive affect: r = 0.54 (?) KPS vs negative affect: r = -0.09 (?) KPS vs satisfaction with life: r = 0.36 (?) KPS vs overall condition: r = 0.39 (?) |  |  |  |
|  | Wood et al. [65] | USA (English) |  |  |  | a1. 37 a2. 37 a3. 37 | a1. I a2. I a3. I | a1. KPS vs ambulation: r = 0.57 (+) a2. KPS vs activity: r = 0.69 (+) a3. KPS vs uptime: r = 0.52 (+) |  |  |  |
|  | **Pooled or summary result**  **(overall rating)** | |  |  |  | **a. 1936 b. 314** | **a. High**  **b. High** | **a.12+ 5- (±)**  **b. 6+ (+)** |  |  |  |

Text in red = outcomes ignored in summary result.

V = very good methodological quality; A = adequate methodological quality; D = doubtful methodological quality; I = inadequate methodological quality.

+ = sufficient; - = insufficient; ? = indeterminate; ± = inconsistent.

AKPS = Australia-modified Karnofsky Performance Status; ATHOS = AIDS Time-Oriented Health Outcome Study; BDI = Beck Depression Inventory; CDC = Centers for Disease Control; CIPS = Cancer Inventory of Problem Situations; ECOG = Eastern Cooperative Oncology Group Performance Status; GDS = Geriatric Depression Scale; HPQ = Health Perceptions Questionnaire; IADL scale = Instrumental Activities of Daily Living Scale; Katz ADL = Katz Activities of Daily Living Scale; KPS = Karnofsky Performance Status; MMSE = Mini Mental State Exam; MOS-HIV = Medical Outcomes Study HIV Health Survey; OSRRS = Older Americans Resources and Services Social Resource and Rating Scale; PPS = Palliative Performance Scale; QWB = Quality of Well-being Scale; VAS = visual analogue scale.

^a^Language in which the instrument was evaluated.

**Results other measurement properties per study: MusculoSkeletal Tumor Society 1987 score**

| **MSTS87** | **Country (language*)** | **Structural validity** | | | **Internal consistency** | | |
| --- | --- | --- | --- | --- | --- | --- | --- |
|  |  | *n* | *Meth qual* | *Result (rating)* | *n* | *Meth qual* | *Result (rating)* |
| Bolia et al. [67] | Greece (Greek) | 100 | A | Unidimensional instrument (7 items):  factor loadings >0.7; explained variation unknown (?) | 100 | V | α = 0.763 (+) |
| Davis et al. [66] | Canada (English) |  |  |  | 97 | V | α = 0.81 (+) |
| **Pooled or summary result**  **(overall rating)** | | **100** | **^b^** | **Unidimensional instrument (7 items) (?)** | **197** | **^b^** | **α = 0.76-0.81 (?)** |

Text in red = outcomes ignored in summary result.

V = very good methodological quality; A = adequate methodological quality; D = doubtful methodological quality; I = inadequate methodological quality.

+ = sufficient; - = insufficient; ? = indeterminate; ± = inconsistent.

^a^ Language in which the instrument was evaluated.

^b^ No level of evidence, rating is indeterminate

| **MSTS87** | **Country (language^a^)** | **Cross-cultural validity/ measurement invariance** | | | **Reliability**  **a = test-retest; b = inter-rater** | | | **Measurement error** | | |
| --- | --- | --- | --- | --- | --- | --- | --- | --- | --- | --- |
|  |  | *n* | *Meth qual* | *Result (rating)* | *n* | *Meth qual* | *Result (rating)* | *n* | *Meth qual* | *Result (rating)* |
| Bolia et al. [67] | Greece (Greek) |  |  |  | a. 100 | a. A | a. ICC = 0.997 (+) |  |  |  |
| Davis et al. [66] | Canada (English) |  |  |  |  |  |  | 97 | I | SEM =  6.4 - 9.3 (?) |
| **Pooled or summary result**  **(overall rating)** | |  |  |  | **a. 100** | **a. Moderate** | **a. ICC = 0.997 (+)** | **97** | **^b^** | **SEM =**  **6.4 - 9.3 (?)** |

Text in red = outcomes ignored in summary result.

V = very good methodological quality; A = adequate methodological quality; D = doubtful methodological quality; I = inadequate methodological quality.

+ = sufficient; - = insufficient; ? = indeterminate; ± = inconsistent.

ICC = intraclass correlation coefficient; SEM = standard error of the mean.

^a^ Language in which the instrument was evaluated.

^b^ No level of evidence, rating is indeterminate

| **MSTS87** | **Country (language^a^)** | **Criterion validity** | | | **Hypotheses testing**  **a = comparison with other instruments**  **b = comparison between subgroups** | | | **Responsiveness**  **a = comparison to gold standard**  **b = comparison with other instruments**  **c = comparison between subgroups**  **d = before and after intervention** | | |
| --- | --- | --- | --- | --- | --- | --- | --- | --- | --- | --- |
|  |  | *n* | *Meth qual* | *Result (rating)* | *n* | *Meth qual* | *Result (rating)* | *n* | *Meth qual* | *Result (rating)* |
| Bolia et al. [67] | Greece (Greek) |  |  |  | a. 100 | a. A | a. MSTS87 vs SF-36: ρ = 0.313 (+) |  |  |  |
| Davis et al. [66] | Canada (English) |  |  |  | a. 97 | a. D | a. MSTS87 vs TESS-LE:  correlation coefficient = 0.49-0.56 (+) | d. 97 | d. I | d. Preop vs 6 weeks, 3, 6, and 12 months postop:  ES = 0.09-0.52 (?) |
| **Pooled or summary result**  **(overall rating)** | |  |  |  | **a. 197** | **a. Moderate** | **a. 2+ (+)** | **d. 97** | **d. ^b^** | **d. Data provided uninterpretable (?)** |

Text in red = outcomes ignored in summary result.

V = very good methodological quality; A = adequate methodological quality; D = doubtful methodological quality; I = inadequate methodological quality.

+ = sufficient; - = insufficient; ? = indeterminate; ± = inconsistent.

ES = effect size; MSTS87 = MusculoSkeletal Tumor Society 1987 score; postop = postoperatively; preop = preoperatively; SF-36 = 36-Item Short Form Survey; TESS-LE = Toronto Extremity Salvage Score -Lower extremity.

^a^ Language in which the instrument was evaluated.

^b^ No level of evidence, rating is indeterminate

**Results other measurement properties per study: MusculoSkeletal Tumor Society 1993 score – Lower extremity**

| **MSTS93-LE** | **Country (language^a^)** | **Structural validity** | | | **Internal consistency** | | |
| --- | --- | --- | --- | --- | --- | --- | --- |
|  |  | *n* | *Meth qual* | *Result (rating)* | *n* | *Meth qual* | *Result (rating)* |
| Davis et al. [66] | Canada (English) |  |  |  | 97 | V | α = 0,91 (+) |
| Enneking et al. [9] | USA (English) |  |  |  |  |  |  |
| Iwata et al. [68] | Japan (Japanese) | 100 | A | Unidimensional instrument (6 items):  factor loadings ≥0.57; explained variation unknown (?) | 100 | V | α = 0.87 (+) |
| Kask et al. [20] | Finnish (Finland) |  |  |  |  |  |  |
| Lee et al. [69] | Korea (Unknown) |  |  |  | 41 | I | α = 0.88 (for the 3 lower extremity specific items) (?) |
| Mallet et al. [21] | France (French) | 188 | V | Unidimensional instrument (6 items):  CFI: 0.993; RMSEA: 0.068 (+) | 188 | V | α = 0.85 (+) |
| Ocaktan et al. [22] | Turkey (Turkish) |  |  |  |  |  |  |
| Rebolledo et al. [23] | Brazil (Brazilian Portuguese) | 67 | A | Unidimensional instrument (6 items):  EFA: factor loadings ≥0.446; explained variation 51%.  CFA: GFI: 0.95; RMSEA: 0.026; TLI: 0.99; CFI: 0.99 (+) | 67 | V | α = 0.84 (+) |
| Rizzo et al. [70] | Italy (Italian) | 59 | I | Unidimensional instrument (6 items):  scree plot shows 1 factor with eigenvalue >1 (?) | 59 | V | α = 0.98 (+) |
| Saebye et al. [71] | Denmark (Danish) |  |  |  | 162 | V | α = 0.79 (+) |
| Xu et al. [72] | China (Mandarin) | 98 | A | Unidimensional instrument (6 items):  EFA: factor loadings ≥ 0.61.  CFA: GFI: 0.93; RMSEA: 0.045; CFI: 0.91; NNFI: 0.92 (+) | 98 | V |  |
| **Pooled or summary result**  **(overall rating)** | | **353** | **High** | **Unidimensional instrument (6 items)** | **674** | **High** | **α = 0.79-0.98 (+)** |

Text in red = outcomes ignored in summary result.

V = very good methodological quality; A = adequate methodological quality; D = doubtful methodological quality; I = inadequate methodological quality.

+ = sufficient; - = insufficient; ? = indeterminate; ± = inconsistent.

CFA = confirmatory factor analysis; CFI = comparative fit index ; EFA = exploratory factor analysis; GFI = goodness-of-fit index; NNFI = non-normed fit index; RMSEA = root mean square error of approximation; TLI = Tucker-Lewis index.

^a^ Language in which the instrument was evaluated.

| **MSTS93-LE** | **Country (language^a^)** | **Cross-cultural validity/ measurement invariance** | | | **Reliability**  **a = test-retest; b = inter-rater** | | | **Measurement error** | | |
| --- | --- | --- | --- | --- | --- | --- | --- | --- | --- | --- |
|  |  | *n* | *Meth qual* | *Result (rating)* | *n* | *Meth qual* | *Result (rating)* | *n* | *Meth qual* | *Result (rating)* |
| Davis et al. [66] | Canada (English) |  |  |  |  |  |  | 97 | I | SEM =  2.5 - 3.1 (?) |
| Enneking et al. [9] | USA (English) |  |  |  | b. 60 | b. I | b. Mean range in ratings ± 3% (?) |  |  |  |
| Iwata et al. [68] | Japan (Japanese) |  |  |  | a. 100 | a. D | a. ICC = 0.92 (+) |  |  |  |
| Kask et al. [20] | Finnish (Finland) |  |  |  |  |  |  |  |  |  |
| Lee et al. [69] | Korea (Unknown) |  |  |  |  |  |  |  |  |  |
| Mallet et al. [21] | France (French) |  |  |  | b. 32 | b. A | b. ICC = 0.94 (+) |  |  |  |
| Ocaktan et al. [22] | Turkey (Turkish) |  |  |  |  |  |  |  |  |  |
| Rebolledo et al. [23] | Brazil (Brazilian Portuguese) |  |  |  | a. 21 b. 40 | a. I b. A | a. ICC = 0.92 (+) b. ICC = 0.98 (+) |  |  |  |
| Rizzo et al. [70] | Italy (Italian) |  |  |  | a. 59 | a. A | a. ICC = 0.98 (+) |  |  |  |
| Saebye et al. [71] | Denmark (Danish) |  |  |  | b. 162 | b. A | b. ICC = 0.88 (+) |  |  |  |
| Xu et al. [72] | China (Mandarin) |  |  |  | a. 98 b. 98 | a. D b. A | a. ICC = 0.91 (+) b. ICC = 0.90 (+) |  |  |  |
| **Pooled or summary result**  **(overall rating)** | |  |  |  | **a. 257**  **b. 332** | **a. Moderate**  **b. High** | **a. ICC = 0.91-0.98 (+)**  **b. ICC = 0.88-0.98 (+)** | **97** | **^b^** | **SEM =**  **2.5 - 3.1 (?)** |

Text in red = outcomes ignored in summary result.

V = very good methodological quality; A = adequate methodological quality; D = doubtful methodological quality; I = inadequate methodological quality.

+ = sufficient; - = insufficient; ? = indeterminate; ± = inconsistent.

ICC = intraclass correlation coefficient; SEM = standard error of the mean.

^a^ Language in which the instrument was evaluated.

^b^ No level of evidence, rating is indeterminate

| **MSTS93-LE** | **Country (language^a^)** | **Criterion validity** | | | **Hypotheses testing**  **a = comparison with other instruments**  **b = comparison between subgroups** | | | **Responsiveness**  **a = comparison to gold standard**  **b = comparison with other instruments**  **c = comparison between subgroups**  **d = before and after intervention** | | |
| --- | --- | --- | --- | --- | --- | --- | --- | --- | --- | --- |
|  |  | *n* | *Meth qual* | *Result (rating)* | *n* | *Meth qual* | *Result (rating)* | *n* | *Meth qual* | *Result (rating)* |
| Davis et al. [66] | Canada (English) |  |  |  | a. 97 | a. D | a. MSTS93-LE vs TESS-LE: data not shown (?) | d. 97 | d. I | d. Preop vs 6 weeks, 3, 6, and 12 months postop:  ES = 0.29-0.52 (?) |
| Enneking et al. [9] | USA (English) |  |  |  |  |  |  |  |  |  |
| Iwata et al. [68] | Japan (Japanese) |  |  |  | a1. 100 a2. 100 | a1. V a2. A | a1. MSTS93-LE vs TESS-LE: r = 0.81 (-) a2. MSTS93-LE vs SF-36 PCS: r = 0.68 (+) MSTS93-LE vs SF-36 MCS: r = 0.043 (+) MSTS93-LE vs SF-36 physical functioning: r = 0.66 (+) MSTS93-LE vs SF-36 role physical: r = 0.62 (+) MSTS93-LE vs SF-36 bodily pain: r = 0.52 (+) MSTS93-LE vs SF-36 general health: r = 0.31 (+) MSTS93-LE vs SF-36 vitality: r = 0.33 (-) MSTS93-LE vs SF-36 social functioning: r = 0.58 (-) MSTS93-LE vs SF-36 role emotional: r = 0.47 (-) MSTS93-LE vs SF-36 mental health: r = 0.25 (+) |  |  |  |
| Kask et al. [20] | Finnish (Finland) |  |  |  | a. 19 | a. V | a. MSTS93-LE vs TESS-LE: ρ = 0.588 (+) |  |  |  |
| Lee et al. [69] | Korea (Unknown) |  |  |  |  |  |  |  |  |  |
| Mallet et al. [21] | France (French) |  |  |  | a. 188 b. 160 | a. A b. D | a. MSTS93-LE vs WOMAC: r = -0.58 (+) b. MSTS93-LE vs performance status (0, 1 and 2):  mean = 23.9 vs 17.5 vs 13.1 (?) MSTS93-LE vs tumor grade (0, 1, 2, 3):  mean = 23.1 vs 23.8 vs 20.2 vs 20.1 (?) |  |  |  |
| Ocaktan et al. [22] | Turkey (Turkish) |  |  |  | a. 23 | a. A | a. MSTS93-LE vs WOMAC: r = -0.634 (+) |  |  |  |
| Rebolledo et al. [23] | Brazil (Brazilian Portuguese) |  |  |  | a. 67 b. 55 | a. V b. D | a. MSTS93-LE vs TESS-LE: r = 0.632 (+) b. No walking aid vs one crutch vs two crutches:  mean = ±27 vs ±17.5 vs ±12.5 (?) Pain med use vs no pain med use:  mean = ±11 vs ±23 (?) |  |  |  |
| Rizzo et al. [70] | Italy (Italian) |  |  |  | a1. 59 a2. 59 | a1. A a2. A | a1. MSTS93-LE vs TESS-LE: r = 0.80 (-) a1. MSTS93-LE vs SF-36: r = 0.50 (+) |  |  |  |
| Saebye et al. [71] | Denmark (Danish) |  |  |  | a1. 162 a2. 162 | a1. V a2. D | a1. MSTS93-LE vs TESS-LE: ρ = 0.83 (-) a2. MSTS93-LE vs TUG: ρ = -0.26 (-) |  |  |  |
| Xu et al. [72] | China (Mandarin) |  |  |  | a. 98 b. 98 | a. V b. D | a. MSTS93-LE vs TESS-LE: r = 0.745 (-) b. Amputation vs limb-salvage surgery:  mean = 18.8 vs 23.5 (+) Male vs female: mean = 21.1 vs 21.4 (?) Age 50+ vs age 50-: mean = 21.6 vs 20.8 (?) |  |  |  |
| **Pooled or summary result**  **(overall rating)** | |  |  |  | **a. 1037**  **b. 98** | **a. High**  **b. Very low** | **a. 12+ 8- (±)**  **b. 1+ (+)** | **d. 97** | **d. ^b^** | **d. Data provided uninterpretable (?)** |

Text in red = outcomes ignored in summary result.

V = very good methodological quality; A = adequate methodological quality; D = doubtful methodological quality; I = inadequate methodological quality.

+ = sufficient; - = insufficient; ? = indeterminate; ± = inconsistent.

ES = effect size; MCS = mental component summary; MSTS93-LE = MusculoSkeletal Tumor Society 1993 score–Lower extremity; PCS = physical component summary; postop = postoperatively; preop = preoperatively; SF-36 = 36-Item Short Form Survey; TESS-LE = Toronto Extremity Salvage Score -Lower extremity; WOMAC = Western Ontario and McMaster Universities Osteoarthritis Index.

^a^ Language in which the instrument was evaluated.

^b^ No level of evidence, rating is indeterminate

**Results other measurement properties per study: Reintegration to Normal Living index**

| **RNL INDEX** | **Country (language^a^)** | **Structural validity** | | | **Internal consistency** | | |
| --- | --- | --- | --- | --- | --- | --- | --- |
|  |  | *n* | *Meth qual* | *Result (rating)* | *n* | *Meth qual* | *Result (rating)* |
| Merz et al. [74] | USA (English) |  |  |  |  |  |  |
| Okoye et al. [24] | Nigeria (IGBO language) |  |  |  |  |  |  |
| Wood-Dauphinee et al. [15, 73] | Canada (English, French) |  |  |  |  |  |  |
| **Pooled or summary result**  **(overall rating)** | |  |  |  |  |  |  |

Text in red = outcomes ignored in summary result.

V = very good methodological quality; A = adequate methodological quality; D = doubtful methodological quality; I = inadequate methodological quality.

+ = sufficient; - = insufficient; ? = indeterminate; ± = inconsistent.

^a^ Language in which the instrument was evaluated.

| **RNL INDEX** | **Country (language^a^)** | **Cross-cultural validity/ measurement invariance** | | | **Reliability**  **a = test-retest; b = inter-rater** | | | **Measurement error** | | |
| --- | --- | --- | --- | --- | --- | --- | --- | --- | --- | --- |
|  |  | *n* | *Meth qual* | *Result (rating)* | *n* | *Meth qual* | *Result (rating)* | *n* | *Meth qual* | *Result (rating)* |
| Merz et al. [74] | USA (English) |  |  |  |  |  |  |  |  |  |
| Okoye et al. [24] | Nigeria (IGBO language) |  |  |  |  |  |  |  |  |  |
| Wood-Dauphinee et al. [15, 73] | Canada (English, French) |  |  |  |  |  |  |  |  |  |
| **Pooled or summary result**  **(overall rating)** | |  |  |  |  |  |  |  |  |  |

Text in red = outcomes ignored in summary result.

V = very good methodological quality; A = adequate methodological quality; D = doubtful methodological quality; I = inadequate methodological quality.

+ = sufficient; - = insufficient; ? = indeterminate; ± = inconsistent.

ICC = intraclass correlation coefficient.

^a^ Language in which the instrument was evaluated.

| **RNL INDEX** | **Country (language^a^)** | **Criterion validity** | | | **Hypotheses testing**  **a = comparison with other instruments**  **b = comparison between subgroups** | | | **Responsiveness**  **a = comparison to gold standard**  **b = comparison with other instruments**  **c = comparison between subgroups**  **d = before and after intervention** | | |
| --- | --- | --- | --- | --- | --- | --- | --- | --- | --- | --- |
|  |  | *n* | *Meth qual* | *Result (rating)* | *n* | *Meth qual* | *Result (rating)* | *n* | *Meth qual* | *Result (rating)* |
| Merz et al. [74] | USA (English) |  |  |  | a1. 928 a2. 928 a3. 928 | a1. A a2. A a3. A | a1. RNL total vs NIHSS total: r = -0.345 (+) a2. RNL total vs PHQ9 total: r = -0.645 (-) a3. RNL total vs SIS strength: r = 0.625 (-) RNL total vs SIS memory: r = 0.594 (+) RNL total vs SIS communication: r = 0.585 (+) RNL total vs SIS ADL: r = 0.761 (-) RNL total vs SIS mobility: r = 0.711 (-) RNL total vs SIS hand function: r = 0.647 (-) RNL total vs SIS participation: r = 0.777 (+) |  |  |  |
| Okoye et al. [24] | Nigeria (IGBO language) | 1. 102  2. 102  3. 102 | 1. V  2. V  3. V | 1. I-RNL vs E-RNL total score:  ρ = 0.95 (+)  2. I-RNL vs E-RNL daily functioning:  ρ = 0.94 (+)  3. I-RNL vs E-RNL perception of self:  ρ = 0.88 (+) |  |  |  |  |  |  |
| Wood-Dauphinee et al. [15, 73] | Canada (English, French) |  |  |  | a1. 70  a2. 70  a3. 70  a4. 194    b. 194 | a1. A  a2. A  a3. A  a4. I    b. I | a1 RNL total score vs QLI total score (initial interview): r = 0.72 (-) RNL total score vs QLI total score (follow-up interview): r = 0.549 (+) Change score total score RNL vs change score total score QLI: r = 0.560 (+) a2. RNL daily functioning vs QLI activity and daily living: r = 0.67 (+) RNL daily functioning vs QLI support and outlook:  r = 0.07 (+) a3. RNL perception of self vs QLI activity and daily living: r = 0.20 (+) RNL perception of self vs QLI support and outlook:  r = 0.36 (+) a4. RNL total score vs QLI total score: r = 0.68 (-) RNL total score vs positive well-being: r = 0.32 (?) RNL total score vs negative well-being: r = -0.41 (?) RNL total score vs general well-being: r = 0.41 (?) b. RNL total score: no relationship with living arrangements (?) RNL total score: no relationship with family status (?) RNL total score: best scores for patients who were working/going to school, lowest scores for patients too sick to work (?) RNL total score: no relationship with presence of problems in living (?) RNL total score: patients with active disease were slightly more likely to have worse scores (?) | d1. 55  d2. 55  d3. 55 | d1. I  d2. I  d3. I | d1. Total score:  change score = 6.5 (14.6);  T-value = 3.28 (?)  d2. Daily Functioning: change score = 8.5 (18.2); T-value = 3.28 (?)  d3. Perception of Self: change score = 2.2 (15.3); T-value = 1.06 (?) |
| **Pooled or summary result**  **(overall rating)** | | **1. 102**  **2. 102**  **3. 102** | **1. High**  **2. High**  **3. High** | **1. Total score (+)**  **2. Daily Functioning (8 items) (+)**  **3. Perception of Self (3 items) (+)** | **a1. 2854**  **a2. 70**  **a3. 70** | **a1. Moderate**  **a2. Low**  **a3. Low** | **a1. Total score: a. 6+ 6- (±)**  **a2. Daily Functioning (8 items): a. 2+ (+)**  **a3. Perception of Self (3 items): a. 2+ (+)** | **d. 55**  **d. 55**  **d. 55** | **d. ^b^**  **d. ^b^**  **d. ^b^** | **d. Total score (?)**  **d. Daily functioning**  **(8 items) (?)**  **d. Perception of Self**  **(3 items) (?)** |

Text in red = outcomes ignored in summary result.

V = very good methodological quality; A = adequate methodological quality; D = doubtful methodological quality; I = inadequate methodological quality.

+ = sufficient; - = insufficient; ? = indeterminate; ± = inconsistent.

ADL = Activities of Daily Living; E-RNL = English version Reintegration to Normal Living index; I-RNL = IGBO version Reintegration to Normal Living index; NIHSS = National Institutes of Health Stroke Scale; PHQ9 = Patient Health Questionnaire-9; QLI = Quality of Life Index; RNL = Reintegration to Normal Living index; SIS = Stroke Impact Scale.

^a^ Language in which the instrument was evaluated.

^b^ No level of evidence, rating is indeterminate

**Results other measurement properties per study: Toronto Extremity Salvage Score – Lower extremity**

| **TESS-LE** | **Country (language^a^)** | **Structural validity** | | | **Internal consistency** | | |
| --- | --- | --- | --- | --- | --- | --- | --- |
|  |  | *n* | *Meth qual* | *Result (rating)* | *n* | *Meth qual* | *Result (rating)* |
| Bolia et al. [67] | Greece (Greek) | 100 | I | Two factor structure (6 and 24 items):  factor loadings ≥0.5, 1 item with cross loading (?) | 100 | I | Total score: α = 0.924 (?) |
| Davis et al. [25] | Canada (English) | ? | I | One factor structure (30 items):  6 items with cross loadings, explained variance 51.70% (?) |  |  |  |
| Davis et al. [13] | Canada (English) |  |  |  | 66 | I | α = 0.94 for the 29 item TESS-LE (?) |
| Davis et al. [25] | Canada (English) | 94 | I | Six factor structure (7, 7, 5, 5, 4, and 2 Items):  9 items with cross loadings, explained variance 71.01% (-) | 94 | I | Total score: α = 0.94 (?) |
| Janssen et al. [75] | USA (English) | 100 | I | Factor score of 0.922 with the underlying trait (defined as 'physical function') (?) |  |  |  |
| Kask et al. [20] | Finland (Finnish) |  |  |  |  |  |  |
| Kask et al. [76] | Finland (Finnish) | 136 | I | Unidimensional instrument (30 items): factor loadings ≥0.515; 1 item with cross loading; explained variation 74.4% (+) | 136 | V | α = 0.97 (+) |
| Kim et al. [26] | Korea (Korean) |  |  |  | 92 | D | α = 0.978 (+) |
| Ocaktan et al. [22] | Turkey (Turkish) |  |  |  | 23 | D | α = 0.96 (+) |
| Ogura et al. [77] | Japan (Japanese) | 100 | I | Two factor structure (15 and 15 items):  factor loadings ≥0.489, 18 items with cross loadings (-) | 100 | I | Total score: α = 0.978 (?) |
| Rossi et al. [27] | Italy (Italian) |  |  |  |  |  |  |
| Saebye et al. [28] | Denmark (Danish) |  |  |  | 16 | D | α = 0.94 (+) |
| Saraiva et al. [29] | Brazil (Brazilian-Portuguese) |  |  |  | 48 | D | α = 0.87 (+) |
| Srisawat et al. [30] | Thailand (Thai) |  |  |  | 70 | D | α = 0.981 (+) |
| Trost et al. [31] | Germany (German) |  |  |  | 32 | D | α = 0.924 (+) |
| Willeumier et al. [78] | The Netherlands (Dutch) |  |  |  | 50 | D | α = 0.957 (+) |
| **Pooled or summary result**  **(overall rating)** | | **330** | **Very low** | **Distinct factor structures (±)** | **467** | **^b^** | **α = 0.87-0.981 (?)** |

Text in red = outcomes ignored in summary result.

V = very good methodological quality; A = adequate methodological quality; D = doubtful methodological quality; I = inadequate methodological quality.

+ = sufficient; - = insufficient; ? = indeterminate; ± = inconsistent.

^a^ Language in which the instrument was evaluated.

^b^ No level of evidence, rating is indeterminate

| **TESS-LE** | **Country (language^a^)** | **Cross-cultural validity/ measurement invariance** | | | **Reliability**  **a = test-retest; b = inter-rater** | | | **Measurement error** | | |
| --- | --- | --- | --- | --- | --- | --- | --- | --- | --- | --- |
|  |  | *n* | *Meth qual* | *Result (rating)* | *n* | *Meth qual* | *Result (rating)* | *n* | *Meth qual* | *Result (rating)* |
| Bolia et al. [67] | Greece (Greek) |  |  |  | a. 100 | a. D | a. ICC = 1 (+) |  |  |  |
| Davis et al. [25] | Canada (English) |  |  |  |  |  |  |  |  |  |
| Davis et al. [13] | Canada (English) |  |  |  | a. 45 | a. D | a. ICC = 0.92-0.97 (+) |  |  |  |
| Davis et al. [25] | Canada (English) |  |  |  |  |  |  |  |  |  |
| Janssen et al. [75] | USA (English) |  |  |  |  |  |  | 100 | I | SEM < 0.32 (?) |
| Kask et al. [20] | Finland (Finnish) |  |  |  |  |  |  |  |  |  |
| Kask et al. [76] | Finland (Finnish) | 1. 136 2. 136 | 1. I 2. I | 1. TESS-LE vs age: ρ = -0.23 (?) 2. TESS-LE vs BMI: ρ = -0.25 (?) | a. 136 | a. A | a. ICC = 0.95 (+) |  |  |  |
| Kim et al. [26] | Korea (Korean) |  |  |  | a. ? | a. I | a. ICC = 0.874 (+) |  |  |  |
| Ocaktan et al. [22] | Turkey (Turkish) |  |  |  | a. 23 | a. A | a. ICC = 0.96 (+) |  |  |  |
| Ogura et al. [77] | Japan (Japanese) |  |  |  | a. 100 | a. I | a. ICC = 0.941 (+) |  |  |  |
| Rossi et al. [27] | Italy (Italian) |  |  |  | a. 63 | a. A | a. ICC = 0.99 (+) | 63 | A | SEM = 1.68 (+) |
| Saebye et al. [28] | Denmark (Danish) |  |  |  | a. ? | a. I | a. ICC = 0.88 (+) |  |  |  |
| Saraiva et al. [29] | Brazil (Brazilian-Portuguese) |  |  |  |  |  |  |  |  |  |
| Srisawat et al. [30] | Thailand (Thai) |  |  |  | a. 70 | a. A | a. ICC = 0.810 (+) |  |  |  |
| Trost et al. [31] | Germany (German) |  |  |  | a. 20 | a. I | a. ICC = 0.952 (+) |  |  |  |
| Willeumier et al. [78] | The Netherlands (Dutch) |  |  |  | a. 25 | a. I | a. ICC = 0.963 (+) |  |  |  |
| **Pooled or summary result**  **(overall rating)** | | **1. 136**  **2. 136** | **1. ^b^**  **2. ^b^** | **1. Age not related to TESS-LE score (?)**  **2. BMI not related to TESS-LE score (?)** | **a. 437** | **a. High** | **a. ICC = 0.810-1 (+)** | **63** | **Low** | **SEM = 1.68 (+)** |

Text in red = outcomes ignored in summary result.

V = very good methodological quality; A = adequate methodological quality; D = doubtful methodological quality; I = inadequate methodological quality.

+ = sufficient; - = insufficient; ? = indeterminate; ± = inconsistent.

ICC = intraclass correlation coefficient; SEM = standard error of the mean.

^a^ Language in which the instrument was evaluated.

^b^ No level of evidence, rating is indeterminate

| **TESS-LE** | **Country (language^a^)** | **Criterion validity** | | | **Hypotheses testing**  **a = comparison with other instruments**  **b = comparison between subgroups** | | | **Responsiveness**  **a = comparison to gold standard**  **b = comparison with other instruments**  **c = comparison between subgroups**  **d = before and after intervention** | | |
| --- | --- | --- | --- | --- | --- | --- | --- | --- | --- | --- |
|  |  | *n* | *Meth qual* | *Result (rating)* | *n* | *Meth qual* | *Result (rating)* | *n* | *Meth qual* | *Result (rating)* |
| Bolia et al. [67] | Greece (Greek) |  |  |  | a. 100 | a. A | a. TESS-LE vs SF-36: ρ = 0.714 (-) |  |  |  |
| Davis et al. [25] | Canada (English) |  |  |  |  |  |  |  |  |  |
| Davis et al. [13] | Canada (English) |  |  |  | a. 101 b. 57 | a. I b. D | a. TESS-LE vs MSTS87: r = 0.48 (+) b. Bone vs soft tissue tumor:  mean = 74 vs 89 (+) | d. 101 | d. D | d. Preop vs 6 weeks postop:  ES = 1.01 (+) 6 weeks postop vs 3 months postop:  ES = 0.31 (-) 3 months to 6 months postop:  ES = 0.02 (-) |
| Davis et al. [25] | Canada (English) |  |  |  | a1. 97    a2. 97  a3. 97  b1. 97  b2. 97  b3. 97 | a1. A    a2. I  a3. I  b1. I  b2. I  b3. D | a1. Preop and 6-weeks, 3 months, 6 months, 12 months postop - TESS-LE vs SF-36 physical functioning:  ρ = 0.88, 0.80, 0.85, 0.89, 0.88 (5+) - TESS-LE vs SF-36 social functioning:  ρ = 0.66, 0.49, 0.65, 0.65, 0.68 (5-)  - TESS-LE vs SF-36 mental health:  ρ = 0.33, 0.33, 0.44, 0.49, 0.43 (5-) - TESS-LE vs SF-36 role physical: ρ = 0.61-0.78 (?) - TESS-LE vs SF-36 bodily pain: ρ = 0.39-0.68 (?) - TESS-LE vs SF-36 general health: ρ = 0.33-0.66 (?) - TESS-LE vs SF-36 vitality: ρ = 0.40-0.53 (?) - TESS-LE vs SF-36 social functioning:  ρ = 0.49-0.68 (?) - TESS-LE vs SF-36 role emotional: ρ = 0.31-0.59 (?) a2. Preop and 6-weeks, 3 months, 6 months, 12 months postop - TESS-LE vs MSTS87: τ = 0.49, 0.54, 0.54, 0.56, 0.50 (5+)  a3. Preop and 6-weeks, 3 months, 6 months, 12 months postop - TESS-LE vs MSTS93:  ρ = 0.68, 0.81, 0.87, 0.78, 0.89 (1+, 4-) b1. TESS-LE scores by global disability: No exact results provided (only graphical) (?) b2. TESS-LE scores by usage of walking aids:  none vs 1 cane vs 2 crutches - 6 weeks postop: mean = 82 vs 69 vs 51 (?) - 3 months postop: mean = 87 vs 62 vs 59 (?) - 6 months postop: mean = 89 vs 65 vs 61 (?) - 12 months postop: mean = 91 vs 69 vs 48 (?) b3. Bone versus soft tissue sarcoma:  mean = 72 vs 84 (+) | b1. 97  b2. 97  b3. 97  d1. 97    d2. 21  d3. 16  d4. 9  d5. 9 | b1. I  b2. I  b3. A  d1. A    d2. I  d3. I  d4. I  d5. I | b1. Relative efficiency at 6 weeks to 3 months, 3 to 6 months and 6 to 12 months of TESS-LE vs MSTS87:  1.44, 1.42, 7.00 (?) b2. Relative efficiency at 6 weeks to 3 months, 3 to 6 months and 6 to 12 months of TESS-LE vs MSTS93-LE:  1.30, 1.42, 2.17 (?) b3. Relative efficiency at 6 weeks to 3 months, 3 to 6 months and 6 to 12 months of TESS-LE vs SF-36 physical functioning: 1.01, 1.85, 2.25 (?) d1. 6 weeks vs 3 months postop:  ES = 0.65 (+) 3 months vs 6 months postop:  ES = 0.74 (+) 6 months vs 12 months postop:  ES = 0.63 (+) preop vs 12 months postop:  ES = 0.43 (+) d2. Preop and 6 weeks, 3, 6 and 12 months postop for STS of proximal thigh: mean = 85.7 vs 74.7 vs 76.3 vs 80.6 vs 83.3 (?) d3. Preop and 6 weeks, 3, 6 and 12 months postop for STS of distal thigh:  mean = 80.6 vs 69.9 vs 73.9 vs 78.5 vs 80.1 (?) d4. 6 weeks, 3, 6 and 12 months postop for bone tumor with graft: mean = 65.6 vs 82.2 vs 85.3 vs 90.8 (?) d5. 6 weeks, 3, 6 and 12 months postop for bone tumor with prosthesis: mean = 39.4 vs 49.4 vs 49.4 vs 77.4 (?) |
| Janssen et al. [75] | USA (English) |  |  |  | a1. 100  a2. 100  a3. 100  a4. 100 | a1. A  a2. A  a3. V  a4. I | a1. TESS-LE vs PROMIS Physical function (CAT):  ρ = 0.848 (+) a2. TESS-LE vs PROMIS Neuro-QoL Mobility (CAT):  ρ = 0.847 (+) a3. TESS-LE vs Lower Extremity Function Scale:  ρ = 0.917 (+) a4. TESS-LE vs MSTS93-LE: ρ = 0.868 (-) |  |  |  |
| Kask et al. [20] | Finland (Finnish) |  |  |  | a. 19 | a. I | a. TESS-LE vs MSTS93-LE: ρ = 0.588 (+) |  |  |  |
| Kask et al. [76] | Finland (Finnish) |  |  |  | a1. 74 a2. 135  a3. 136  b1. 136 | a1. I a2. A  a3. A  b1. I | a1. TESS-LE vs MSTS93-LE: ρ = 0.59 (+) a2. 15D mobility dimension: ρ = 0.76 (-) 15D general index: ρ = 0.75 (-) a3. TESS-LE vs EORTC QLQ-C30 physical function:  ρ = 0.83 (+) TESS-LE vs EORTC QLQ-C30 global QoL: ρ = 0.71 (-) b1. Male vs female: no difference in score (?) |  |  |  |
| Kim et al. [26] | Korea (Korean) |  |  |  | a1. 92 a2. 92  b. 92 | a1. I a2. A  b. D | a1. TESS-LE vs MSTS93-LE: ρ = 0.772 (-) a2. TESS-LE vs EORTC QLQ-C30 physical functioning: ρ = 0.840 (+)  TESS-LE vs EORTC QLQ-C30 role functioning:  ρ = 0.762 (+) TESS-LE vs EORTC QLQ-C30 social functioning:  ρ = 0.548 (-) TESS-LE vs EORTC QLQ-C30 quality of life:  ρ = 0.791 (-) b. Bone vs soft tissue sarcoma:  mean = 72.9 vs 86.2 (+) Bone resection done vs not done:  mean = 71.4 vs 88.6 (?) Limb salvage vs amputation:  mean = 80.0 vs 78.2 (?) |  |  |  |
| Ocaktan et al. [22] | Turkey (Turkish) |  |  |  | a. 23 | a. A | a. TESS-LE vs WOMAC: r = -0.702 (+) |  |  |  |
| Ogura et al. [77] | Japan (Japanese) |  |  |  | a1. 100 a2. 100 | a1. I a2. A | a1. TESS-LE vs MSTS93-LE: ρ = 0.811 (-) a1. TESS-LE vs SF-36 PCS: ρ = 0.785 (-) TESS-LE vs SF-36 MCS: ρ = -0.106 (+) TESS-LE vs SF-36 physical functioning: ρ = 0.781 (+) TESS-LE vs SF-36 role physical: ρ = 0.703 (+) TESS-LE vs SF-36 bodily pain: ρ = 0.466 (+) TESS-LE vs SF-36 general health: 0.214 (-) TESS-LE vs SF-36 vitality: ρ = 0.327 (-) TESS-LE vs SF-36 social functioning: ρ = 0.568 (-) TESS-LE vs SF-36 role emotional: ρ = 0.467 (-) TESS-LE vs SF-36 mental health: ρ = 0.148 (+) |  |  |  |
| Rossi et al. [27] | Italy (Italian) |  |  |  | a1. 63 a2. 63 a3. 63 | a1. I a2. A a3. A | a1. TESS-LE vs MSTS87: r = 0.66 (-) a2. TESS-LE vs EQ-5D-5L: r = 0.62 (-) a3. TESS-LE vs SF-36 physical functioning:  r = 0.76 (+) | d. 63 | d. D | d. Preop vs 3 months postop:  SRM = 0.28 (+) Preop vs 6 months postop:  SRM = 0.23 (+) 3 months postop vs 6 months postop: SRM = 0.31 (-) |
| Saebye et al. [28] | Denmark (Danish) |  |  |  | a. 16 | a. A | a. TESS-LE vs EORTC QLQ-C30: ρ = 0.90 (-) |  |  |  |
| Saraiva et al. [29] | Brazil (Brazilian-Portuguese) |  |  |  | b. 48 | b. D | b. Use of none vs 1 cane/crutch vs 2 crutches:  mean = 88.4 vs 92.4 vs 94.0 (?)  Use of none vs pain meds:  mean = 91.3 vs 77.1 (?) Amputation vs prosthesis:  mean = 94.4 vs 87.0 (?) |  |  |  |
| Srisawat et al. [30] | Thailand (Thai) |  |  |  | a. 70 | a. I | a. TESS-LE vs MSTS93: r = 0.636 (+) |  |  |  |
| Trost et al. [31] | Germany (German) |  |  |  | a. 32 | a. A | a. TESS-LE vs SF-36 PCS: ρ = 0.741 (-) TESS-LE vs SF-36 MCS: ρ = 0.570 (-) |  |  |  |
| Willeumier et al. [78] | The Netherlands (Dutch) |  |  |  | a. 50 | a. A | a. TESS-LE vs SF-36 physical functioning:  ρ = 0.737 (+) TESS-LE vs SF-36 role physical functioning:  ρ = 0.766 (+) TESS-LE vs SF-36 social functioning: ρ = 0.810 (-) TESS-LE vs SF-36 role emotional functioning:  ρ = 0.511 (-) TESS-LE vs SF-36 mental health: ρ = 0.505 (-) TESS-LE vs SF-36 vitality: ρ = 0.704 (-) TESS-LE vs SF-36 bodily pain: ρ = 0.777 (-) TESS-LE vs SF-36 general health: ρ = 0.540 (+) TESS-LE vs SF-36 PCS: ρ = 0.811 (-) TESS-LE vs SF-36 MCS: ρ = 0.429 (-) |  |  |  |
| **Pooled or summary result**  **(overall rating)** | |  |  |  | **a.1207**  **b. 246** | **a. High**  **b. Moderate** | **a. 21+ 32- (±)**  **b. 3+ (+)** | **d. 261** | **d. Moderate** | **d. 7+ 3- (±)** |

Text in red = outcomes ignored in summary result.

V = very good methodological quality; A = adequate methodological quality; D = doubtful methodological quality; I = inadequate methodological quality.

+ = sufficient; - = insufficient; ? = indeterminate; ± = inconsistent.

15D = 15-Dimensional Health-Related Quality of Life Instrument; CAT = Computerized Adaptive Testing; ES = effect size; EORTC QLQ-C30 = European Organization for Research and Treatment of Cancer Quality of Life Questionnaire Core 30; EQ-5D-5L = EuroQol 5-Dimension 5-Level questionnaire; MCS = mental component summary; MSTS87 = MusculoSkeletal Tumor Society 1987 score; MSTS93-LE = MusculoSkeletal Tumor Society 1993 score – Lower extremity; PCS = physical component summary; postop = postoperatively; preop = preoperatively; PROMIS = Patient-Reported Outcomes Measurement Information System; QoL = Quality of Life; SF-36 = 36-Item Short Form Survey; STS = soft tissue sarcoma; TESS-LE = Toronto Extremity Salvage Score -Lower extremity.

^a^ Language in which the instrument was evaluated.
